# Dynamics of Gut Microbiome in Giant Panda Cubs Reveal Transitional Microbes and Pathways in Early Life

**DOI:** 10.3389/fmicb.2018.03138

**Published:** 2018-12-18

**Authors:** Min Guo, Jianwei Chen, Qiongfang Li, Ying Fu, Guangyi Fan, Jinmin Ma, Ling Peng, Liyun Zeng, Jing Chen, Yayu Wang, Simon Ming-Yuen Lee

**Affiliations:** ^1^State Key Laboratory of Quality Research in Chinese Medicine, Institute of Chinese Medical Sciences, University of Macau, Macau, China; ^2^BGI-Qingdao, BGI-Shenzhen, Qingdao, China; ^3^BGI-Shenzhen, Shenzhen, China; ^4^Faculty of Science and Technology, University of Macau, Macau, China; ^5^Realbio Genomics Institute, Shanghai, China

**Keywords:** giant panda cubs, gut microbiota, co-abundance genes, important microbes, gut microbial gene functions and pathways

## Abstract

Adult giant pandas (*Ailuropoda melanoleuca*) express transitional characteristics in that they consume bamboos, despite their carnivore-like digestive tracts. Their genome contains no cellulolytic enzymes; therefore, understanding the development of the giant panda gut microbiome, especially in early life, is important for decoding the rules underlying gut microbial formation, inheritance and dietary transitions. With deep metagenomic sequencing, we investigated the gut microbiomes of two newborn giant panda brothers and their parents living in Macao, China, from 2016 to 2017. Both giant panda cubs exhibited progressive increases in gut microbial richness during growth, particularly from the 6th month after birth. Enterobacteriaceae dominated the gut microbial compositions in both adult giant pandas and cubs. A total of 583 co-abundance genes (CAGs) and about 79 metagenomic species (MGS) from bacteria or viruses displayed significant changes with age. Seven genera (*Shewanella, Oblitimonas, Helicobacter, Haemophilus, Aeromonas, Listeria*, and *Fusobacterium*) showed great importance with respect to gut microbial structural determination in the nursing stage of giant panda cubs. Furthermore, 10 orthologous gene functions and 44 pathways showed significant changes with age. Of the significant pathways, 16 from *Escherichia, Klebsiella, Propionibacterium, Lactobacillus*, and *Lactococcus* displayed marked differences between parents and their cubs at birth, while 29 pathways from *Escherichia, Campylobacter* and *Lactobacillus* exhibited significant increase in cubs from 6 to 9 months of age. In addition, oxidoreductases, transferases, and hydrolases dominated the significantly changed gut microbial enzymes during the growth of giant panda cubs, while few of them were involved in cellulose degradation. The findings indicated diet-stimulated gut microbiome transitions and the important role of Enterobacteriaceae in the guts of giant panda in early life.

## Introduction

The giant panda, a bamboo specialist, harbors hundreds of bacterial operational taxonomic units (OTUs) in its gut as an adult, mostly from Firmicutes or Proteobacteria, and dominated by *Escherichia, Shigella*, and *Streptococcus* (Xue et al., 2015). However, despite their high fiber diet, cellulolytic species typically present in herbivore guts, e.g., members of *Ruminococcaceae* or *Bacteroides* (Zhang et al., 2018), are conspicuously absent or present at extremely low abundances in the gut of giant panda. Instead, *Bacillus* (Zhou et al., 2015) and *Clostridia*, which have limited cellulolytic activity and thus cannot fully utilize bamboo (Zhu et al., 2011), are widely observed (Dierenfeld et al., 1982). Furthermore, the gut microbiota compositions of adult giant pandas exhibited extensive seasonal variation (Xue et al., 2015; Wu et al., 2017). This variation may indicate disturbance, or it may be a normal feature of the healthy adult gut microbiome of giant pandas. Disorder of gut microbiota structure can be triggered by an abnormal increase in internal enteropathogens or invasion by exogenous pathogens (Zhao et al., 2017a; Zhang et al., 2017). In contrast, biofilm (Hall-Stoodley et al., 2004) or surfactin (Zhou et al., 2018) secreted by internal microbes of giant panda can stabilize the gut microbial structure.

Study of gut microbiome transitions in the giant panda cub during the nursing stage can be of high utility to decipher the mechanism shaping the adult gut microbiome. Regarding giant panda cub, the nursing stage is approximately 18 months (Xue et al., 2015). For the first 2 months after birth, cubs rely on breast milk feeding; they obtain essential nutrients, such as oligosaccharides, microorganisms and exosomal microRNAs (Ma et al., 2017), from maternal milk, as distinct from subsequent formula milk (Zhang et al., 2015); this prepares them for rapid postnatal development. Cessation of exclusive breast feeding drives the maturation of cub gut microbiota into adult-like microbiota (Backhed et al., 2015). During the first 6 months after birth, giant panda cubs display a stable and gender-independent growth rate with age; rapid growth begin at 7 ~ 20 days (Zhang et al., 2015), and the inheritable maximum growth rate occurs at 75 ~ 120 days (Che et al., 2015). A recent study of the gut microbiomes of captive-born giant pandas (S1 group: <2-month-old cubs with breast milk diet; S2 group: 3 ~ 12-month-old cubs with formula milk diet; S3 group: >6-month-old juveniles with bamboos stem or leaf diet; S4: >6-month-old juveniles with bamboo shoot diet) indicated substantial increases in microbial diversity and gene abundance with age: 51 16S-rDNA OTUs (from Clostridiaceae, Veillonellaceae, Peptostreptococcaceae, Lachnospiraceae) and 26 metagenome linking groups (MLGs) (from *Microbacterium, Clostridium, Citrobacter, Delftia, Enterobacter, Klebsiella, Raoultella, Serratia, Enterococcus, Lactobacillus*, and *Staphylococcus*) showed significant differences between the S1 and S3/S4 groups and among S1, S2, S3, and S4, respectively, none of which belong to potentially cellulolytic species. Furthermore, abundant genes related to hemicellulose degradation were detected (Guo et al., 2018; Zhang et al., 2018). In total, the abundance of 81 KEGG orthologous groups (KOs), which are involved in flagellar assembly, ABC transporters, a two-component system, the secretion system, transcription factors, phenylalanine metabolism, purine metabolism, pentose and glucuronate interconversion, pyruvate metabolism, methane metabolism and glycerolipid metabolism, changed significantly with age. Specifically, certain genes related to phenylalanine metabolism, fatty acid biosynthesis, purine metabolism, glutathione metabolism, antibiotic resistance and streptomycin biosynthesis were abundant in groups S3 and S4 (Zhang et al., 2018).

Studies of the gut microbiome of giant panda cub are quite rare, and no study has yet explored gut microbiome transitions of giant panda cub during the growth process. Answers to questions relating to the giant panda nursing period, for instance the relevance of inheritance of the gut microbiome from parents, transitions in gene functions, and significantly changed microbial pathways, remain to be clarified. A detailed investigation of the gut microbiome of giant panda cub during the growth period after birth is urgently needed. In this study, we concentrated on the gut microbiome of newborn giant panda cubs in the nursing stage (samples were grouped by cub age, C0: 0 ~ 1.5-month-old cub fecal samples; C1: 1.5 ~ 6-month-old cub fecal samples; C2: 6 ~ 9-month-old cub fecal samples) and parents (F: father fecal samples; M: mother fecal samples), to demonstrate their gut microbiome transitions in the 1st year after birth.

## Materials and Methods

### Samples and Microbial DNA Extraction

Presently, a giant panda family with four members is living in Macao Giant Panda Pavilion (Father: ‘Kai kai’, born in July, 2007, designated code P1; mother: ‘Xin xin’, born in August, 2008, designated code P2; elder brother: ‘Jian jian’, born in June 26th, 2016, designated code P3; Younger brother: ‘Kang kang’, born in June 26th, 2016, designated code P4). The two cubs were born 3 h apart. They were fed with breast milk until 1.5 months after birth, and then fed mainly with formula milk powder (mixtures of dog and cat milk powders). Due to the specificity of species and sampling times for the study, we obtained a total of 22 fecal samples from the two giant panda cubs in the 1st year of the nursing stage, and we also collected 11 fecal samples from the parents after the cubs were born.

**Table:** 

Individual	Family relationship	Age (by August, 2017)	Sampling period	Sample count
P1	Father	~10 years old	February, 2017 ~ August, 2017	5
P2	Mother	~9 years old	March, 2017 ~ August, 2017	6
P3	Child	~1 year old	0 ~ 9 month(s) old	11
P4	Child	~1 year old	0 ~ 9 month(s) old	11

After fecal samples were collected, we immediately extracted microbial DNA; all experimental steps were performed in a biosafety cabinet where possible. Owing to the huge differences in fecal size and components between cub feces and adult feces, we adopted different strategies to extract the microbial DNA. Approximately 1.0 g cub fecal samples were weighed and subjected to microbial DNA extraction, as described in the manual of the Power water DNA isolation kit (QIAGEN, Germany). Approximately 20 g adult giant panda samples were weighed into a sterilized BagFilter partitioned by a 280 μm pore size membrane. Then, we homogenized the BagFilter for approximately 3 min in InterScience BagMixer 400SW (INTERSCIENCE, France). Following that, the filtrates were guided into a sterilized rapid flow Biofilter with a 0.22 μm pore size filter membrane to collect bacteria, fungi and other organisms or materials with a size larger than 0.22 μm. Via the two-level cascaded filtrations, we enriched fecal microbes on the 0.22 μm pore size membrane, and then extracted microbial DNAs on the membrane using the Power water DNA isolation kit for subsequent metagenomic sequencing. We added the sample collection time to individually assigned codes to yield DNA sample names.

### Library Construction and Sequencing

Before sequencing, the extracted microbial DNAs were fragmented to 250 ~ 500 base pairs (bps) using Covaris E220 and AMPure XP beads (AGENCOURT). Next, the DNA fragments were repaired to generate a blunt end and we further modified its 3′end to obtain a dATP sticky end. Both ends of the DNA fragments were ligated with dTTP tailed adapter sequences and we then amplified these clones for eight PCR cycles. A single-strand circularization process was subsequently performed to generate a single-strand circular DNA library. Afterward, libraries were sequenced on the BGISEQ-500 platform in a paired-end model to obtain a read 100 bp in length.

### Metagenomic Assembly and Taxonomical Annotation

The raw reads were subjected to quality control by SOAPnuke (1.5.6) (Chen et al., 2018), to remove low-quality and adapter contaminated reads. In addition, reads from giant panda genome or bamboo genome were filtered with SNAP (v1.0beta.23) (Zaharia et al., 2011). The remaining high-quality microbial reads of each fecal sample were assembled using IDBA-UD (v1.1.3) (Peng et al., 2012). MetaGeneMark (v3.38) (Zhu et al., 2010) was used to search coding sequences (CDSs) on the assembled contigs longer than 200 bp. Additionally, all 25 gene sets were merged and then clustered with 95% identity by Cd-hit (v4.6.4) (Li and Godzik, 2006), to construct a non-redundant gene set (Forslund et al., 2015). Taxonomic identification of the non-redundant gene set was executed with Blast+ (v2.2.31) (Camacho et al., 2009) by aligning these genes against the Nucleotide Sequence Database (Nt database) (v20170519) at *e*-value ≤ 1e-5. Here, when the gene length coverage mapped by the Nt database was less than 50%, the gene was assigned “Unclassified” status. For each sample, taxonomic sources of genes were annotated by aligning high quality microbial reads to the non-redundant gene set using Bowtie2 (v2.2.5) (Langmead and Salzberg, 2012), and gene abundance profiling was then calculated by Pathoscope2 (v2.0.6) (Hong et al., 2014). Species abundance was determined by the sum of assigned gene counts (Forslund et al., 2015; Jie et al., 2017). Microbes with relative abundance below 1e-8 were removed, and a taxonomic tree of microbes was built according to the NCBI taxonomy. The Shannon–Wiener index (Shannon, 2001) was used to assess the compositional homogeneity of the microbial communities in each sample, and the Pielou homogeneity index (Pielou, 1969) was used to assess the evenness of the microbial communities in each sample. Abundance heatmaps and weighted principal coordinates analysis (PCOA) were executed using the R (v3.1.1) ‘gplots’ package (Warnes et al., 2014) and “ape” package (Dray and Dufour, 2007), respectively.

### CAGs Clustering and Biomarker CAGs Discovery

We used Pearson’s correlation coefficients (PCCs) to define the distance from a randomly picked ‘seed’ gene and other genes correlated with the seed gene; such genes were considered as the “canopy” (Nielsen et al., 2014). Canopies with median abundance correlations at a PCC distance larger than 0.97 were merged as a CAG. Metagenomic species (MGS) were determined by CAGs with more than 20 strongly correlated genes. In cases where less than 50% of the obtained genes were annotated to genomes from the same species, the CAGs were marked as ‘group.’ To identify the significant CAGs among age groups, we first used a Wilcoxon test to identify genes with significantly different abundances among age groups. The canopy algorithm assigned and classified these genes into their original CAGs. Thus, the involved CAGs were collapsed, and only contained significantly changed genes. The abundance of each significant CAG was determined by the median abundance of the genes it contained. We next used LEfSe program (Segata et al., 2011) to discover significantly changed CAGs among age groups. Here, we set the LDA effect size to 2.0 for screening of representative CAGs (microbial species) among samples.

### Structurally Important Microbes

We used two approaches to discover structurally important microbes. The first approach focused on microbial genera with more than 70% occurrence frequency in all investigated samples. Correlations between the ‘stable’ genera were measured by their abundance profiles in samples. We then removed non-significant correlations (*t*-test, *p* > 0.05). Finally, the significant spearman correlation network of microbial genera was displayed in Cytoscape (v3.5.1) (Shannon et al., 2003). Secondly, we built random forest (RF) models for the age groups through random permutation of genus-level abundance profiles (*n* = 500 permutations). The out-of-bag (OOB) error rate was used to assess model accuracy. Furthermore, microbial importance was measured by the mean decrease in the Gini index at each node of the constructed model, where a higher value for the microbe means that it is more important in determining the gut microbial structure of giant panda.

### Gene Function and Pathway Annotations

The non-redundant gene set was annotated by blasting translated proteins of the non-redundant genes against the Orthologous Groups of proteins (COG) database (v20090331) (Galperin et al., 2015) and Kyoto Encyclopedia of Genes and Genomes (KEGG) database (v81) (Kanehisa et al., 2016) with BLAST (v2.2.23) (Altschul et al., 1990). COG-annotated gene functions of samples in each age group were visualized using boxplots [‘Lattice’ R package (Zuur et al., 2009)]. Significantly changed gene functions and pathways between age groups were detected using the Wilcoxon test [adjusted *p* < 0.05, by Holm method (Fu et al., 2014)].

### Enzymatic Annotation and Genes Related to Bamboo Degradation

We obtained enzyme commission numbers (ECs) of genes by KEGG annotation. The abundance of ECs was determined by the sum of assigned gene abundances, and significantly changed ECs between age groups were then detected by Wilcoxon test (adjusted *p* < 0.05, by Holm method). The network of significant ECs was displayed in Cytoscape (Shannon et al., 2003). We further annotated non-redundant genes by blasting against the Carbohydrate-Active enZYmes (CAZy) database. For further confirmation, we aligned the hit genes in the CAZy database against the Pfam database. Abundance profiles of genes relating to bamboo polysaccharides digestion were visualized with the R ‘image.plot’ function.

## Results

### Sequencing, Assembly and Gene Prediction

**FIGURE 1:**
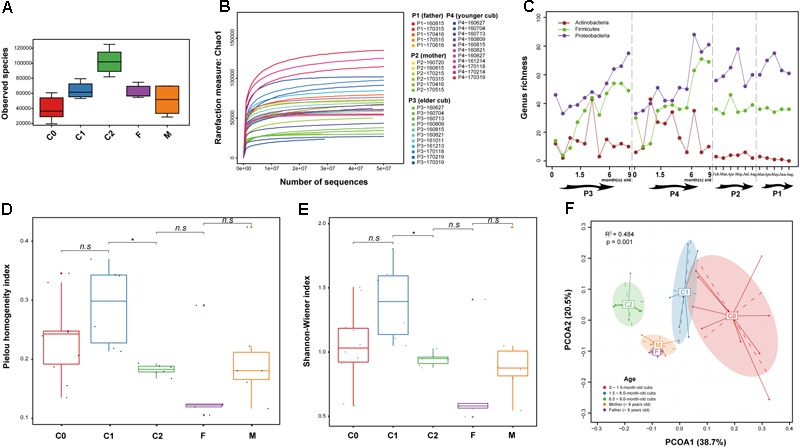
Richness, diversity and correlations of samples. **(A)** Observed microbial species richness in samples. **(B)** Changes in the Chao 1 index (richness) in sequences (reads) rarefaction. **(C)** Genus-level microbial richness of samples. **(D)** Pielou homogeneity index of the microbial communities in each sample. The significance of differences between groups was evaluated by Wilcoxon test (^∗^*p* ≤ 0.05; *^n.s^p* > 0.05). **(E)** Shannon diversity index, which pertains to the microbial diversity of samples; the larger the index, the greater microbial diversity the sample will possess. The significance of diversity between groups was evaluated by Wilcoxon test (^∗^*p* ≤ 0.05; *^n.s^p* > 0.05). **(F)** Weighted principal coordinates analysis (weighted PCOA, Whittaker index matrix) by age. The distance between samples indicated the compositional correlations between them; a closer distance means a stronger correlation between two samples. Significance differences between age groups were assessed by the Adonis method.

The deep sequencing yielded an average of approximately 15.5 Gb raw data for each DNA sample. We first removed reads of low quality, reads polluted by adapter sequences, and reads from the giant panda or bamboo genomes, revealing a high concentration of microbial DNA fragments in extracted total DNAs (Supplementary Table S1). The remaining reads assigned to each sample (average of 14.1 Gb each) are sufficient to depict the microbial richness of the sample (Figures 1A,B). After assembling the microbial reads of each sample, the total length of generated scaffolds ranged from 6,450,400 bp to 364,942,569 bp, and the N50s of the scaffolds of samples ranged from 918 bp to 24,219 bp (average: 3466 bp; median: 1872 bp) (Supplementary Table S2). We then predicted genes on these scaffolds. About 12,952 genes assigned to Metazoa or Viridiplantae were stripped out from the non-redundant microbial gene set. The final non-redundant microbial gene set contained 1,040,648 microbial (or bacterial and viral) genes. The microbial gene set of each sample is described in Supplementary Table S3.

### Gut Microbial Composition During Growth of the Cubs and Their Parents

After filtering microbes with relative abundances below 1e-8, 1,429 microbial species belonging to 328 genera and 134 families were discovered in fecal samples. The 328 microbial genera were phylogenetically clustered by NCBI taxonomy (Supplementary Figure S1). In contrast to the relatively stable fluctuations of microbial genera-level richness in parental samples, the compositional changes of these genera were characterized by progressive increases of gut microbial richness after birth, particularly in 6 ~ 9-month-old cubs (Supplementary Figure S1 and Figures 1A–C). Specifically, Firmicutes rapidly propagated after birth. The majority of Proteobacteria occurred a little later (Supplementary Figure S1); however, genera of Proteobacteria substantially expanded the gut microbial richness (Figure 1C). Furthermore, compositional evenness and diversity of the gut microbiota of 6 ~ 9-month-old cubs significantly differed from that of 1.5 ~ 6.0-month-old cubs (Wilcoxon test, *p* ≤ 0.05): gut microbial evenness and diversity increased as the cubs developed, yet reverted back to the level of their parents after 6 months of age (Figures 1D,E). This suggests that the environment significantly drives microbial colonization of the gut immediately after birth. However, the dramatic expansion of gut microbiota in the early life of giant panda was initially chaotic and sporadic, for example in the case of Actinobacteria (Figure 1C), suggesting that as cubs and their gut microbiota mature, diversity converges toward adult profiles.

Genera of family Enterobacteriaceae, for instance *Escherichia, Klebsiella, Shigella, Salmonella, Citrobacter*, were particularly dominant from birth (Supplementary Figure S1). In addition, we noticed that some genera from other bacterial families, for instance *Oblitimonas, Pseudomonas, Clostridioides, Paeniclostridium, Clostridium, Lactococcus, Enterococcus, Streptococcus, Staphylococcus, Cronobacter, Vibrio, Raoultella, Pectobacterium* (pectin degradation), *Leclercia, Pantoea, Campylobacter* and *Fusobacterium*, and several genera in viral families Podoviridae (*P22virus*), Siphoviridae (*Lambdavirus*), Myoviridae (*P1virus, P2virus*, and *T4virus*) were also in high abundance from birth or enriched soon after birth and stayed in high abundance in parental samples. As a complement to reported prime eukaryotic viral families, for instance Genomoviridae, Picornaviridae, Picornavirales, and Picobirnavirus-like, in the gut of adult giant panda (Zhang et al., 2017), the genera from three prokaryotic double-stranded DNA viral families dominated the gut of giant pandas, and were implicated in the infection of numerous bacteria and archaea (Ackermann, 2003). The major hosts of bacteriophages in samples were *Enterobacteria*, followed by *Escherichia, Salmonella, Klebsiella, Lactococcus*, and *Streptococcus*.

Notably, certain gut microbial (opportunistic) pathogens, such as *Campylobacter, Staphylococcus, Streptococcus, Escherichia, Bacillus, Clostridium, Salmonella, Vibrio*, and *Shigella*, emerged in high abundance in the gut of giant panda cubs soon after birth. Resisting the propagation of these microbes is of great importance for keeping cubs healthy. Apart from certain members of Bacillaceae that are able to secrete antipathogenic surfactin (Zhou et al., 2018), both *Lactobacillus* and *Bifidobacterium* show antimicrobial activities (Liu et al., 2017), and aid milk digestion by converting lactose into lactic acid. The abundance of *Lactobacillus* and *Bifidobacterium* increased quickly after the birth of the cubs, but declined rapidly in adults, particularly *Bifidobacterium*. More curiously, in the gut of cubs, the timings of their rapid increases were different. Rapid growth of *Lactobacillus* appeared within the 1st month after birth, when the cubs were under exclusive breast feeding; *Bifidobacterium* quickly showed enrichment from the 2nd month after birth, when the cubs began to feed mainly on formula milk. As is well known, breast milk is rich in oligosaccharides, whereas formula milk is rich in lactose (Zhang et al., 2015). During the diet transition (from breast milk feeding to formula feeding), we found that some genera from other bacterial or viral families emerged only when formula feeding was started, including a viral family Caliciviridae and a few bacterial families including Hydrogenothermaceae, Nitrosomonadaceae, Comamonadaceae, Oxalobacteraceae, Helicobacteraceae, Desulfuromonadaceae, Cellvibrionaceae, Oceanospirillaceae, Psychromonadaceae, Thermoanaerobacteraceae, and Clostridiales Family XIII. Incertae Sedis, and several other families, were in low abundance at birth, but quickly enriched when the cubs were fed with formula milk, for instance Clostridiaceae, Aeromonadaceae, and Campylobacteraceae.

Rapid expansion of the cub microbiome began at around 10 days after birth, and the greatest increase in membership was seen at about 70 and 100 days, respectively (Supplementary Figure S2). However, the most significant phylogenetic divergence of gut microbial composition from their parents was seen from 180 days of age: in the weighted PCOA (Whittaker index matrix), samples from 0 ~ 1.5-month-old cubs (C0), 1.5 ~ 6-month-old cubs (C1), 6 ~ 9-month-old cubs (C2) and parents (F and M) clustered separately (*R*^2^ = 0.484, *p* = 0.001) (Figure 1F). Like the F and M groups, the C0 and C1 groups were closer, yet both of them showed a distinct distance from the C2 group. This reflected that the gut microbiome of newborn giant panda diverged quickly from that of their parents after birth, and within the nursing stage, particularly at 6 months old, their gut microbial compositions were strongly influenced by the environment (Rothschild et al., 2018), and gradually diverged from the original gut microbial compositions.

### Significantly Changed Gut Microbes in the Growth of Giant Panda Cubs

**FIGURE 2:**
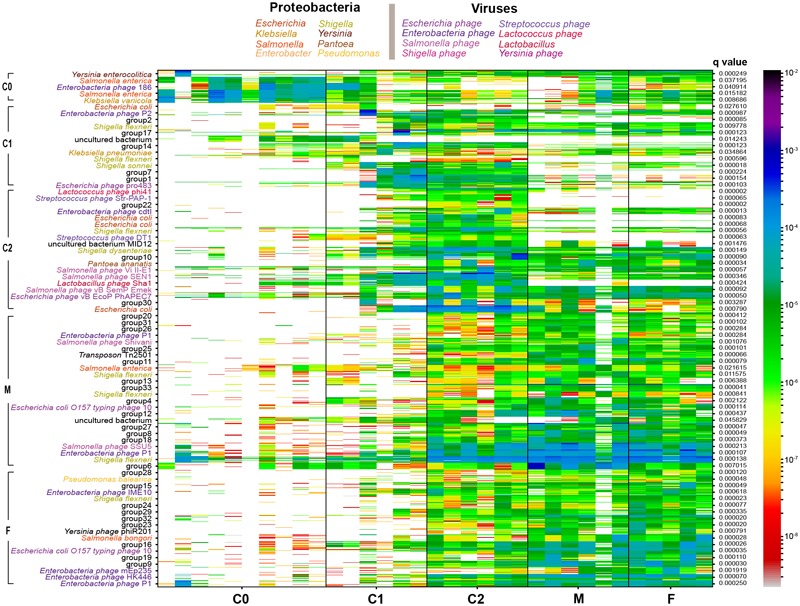
Co-abundance genes (CAG) analysis of genes in samples. The heatmap profile shows significantly changed abundance of related genes [Pearson’s correlation coefficients (PCC) distance > 0.97] in CAG. The ‘group’ label means lower than 50% contained genes of same-species origin. The *q*-value shows the significance of CAGs by Wilcoxon test (*q* < 0.05). Species from the same genus were colored as in the figure legend.

It was difficult to distinguish representative gut microorganisms between 0 ~ 1.5-month-old (C0) and 1.5 ~ 6-month-old (C1) cubs. Nevertheless, when comparing the gut microbial compositions between C0/C1 cubs and 6 ~ 9-month-old cubs (C2) (with LDA score > 4.0) at the microbial family level, we discovered that Enterobacteriaceae is the representative family of C0/C1 cubs, and Lactobacillaceae and Campylobacteraceae are the representative families of C2 cubs. Remarkably, the representative gut microbiota families of C2 cubs are consistent when compared with either C0/C1 or F/M. In addition, compared with the gut microbiota of C2 group cubs, the representative families of paternal and maternal gut microbiota are Enterobacteriaceae and Clostridiaceae, respectively (Supplementary Figure S3). At the species level, co-abundance gene (CAG) analysis yielded 583 CAGs, and indicated that about 79 MGS represented all significantly changed microbes among age groups (Wilcoxon test, adjusted *p* < 0.05; LDA score > 2.0) (Figure 2). Except for unclear species (‘group’), the rest of these species belong to Proteobacteria and viruses. The bacterial CAGs that changed significantly were mostly from Enterobacteriaceae (*Escherichia, Klebsiella, Shigella, Salmonella*, and *Enterobacter*). *Pantoea ananatis*, which shows quorum sensing and drives gene expression, was significantly enriched in C2 groups, and bacteriophages linked with Lactobacillales (*Streptococcus phage, Lactobacillus phage*, and *Lactococcus phage*) were also significantly increased in the C2 group, yet they dramatically decreased in parental groups.

A number of microbes were detected in the gut of giant pandas at birth (P3: 85 genera; P4: 84 genera) and most of them occurred at low abundance (Supplementary Table S4). In spite of this, many of these pioneer families showed rapid enrichment after birth. Among the pioneer genera, *Escherichia, Klebsiella*, and *Shigella* showed inborn high abundance, and the two brother cubs shared 53 bacterial or viral genera. We noticed that some genera stably emerged in all investigated samples, and an overwhelming majority of the pioneer genera were also included among these genera. This reflected that these stable microbial communities probably play general and important roles in the gut microbial structure formation, food utilization and physiology of giant panda. In view of this, we refined microbial genera showing an occurrence frequency of higher than 70% in all investigated samples, and 112 genera were finally screened out.

**FIGURE 3:**
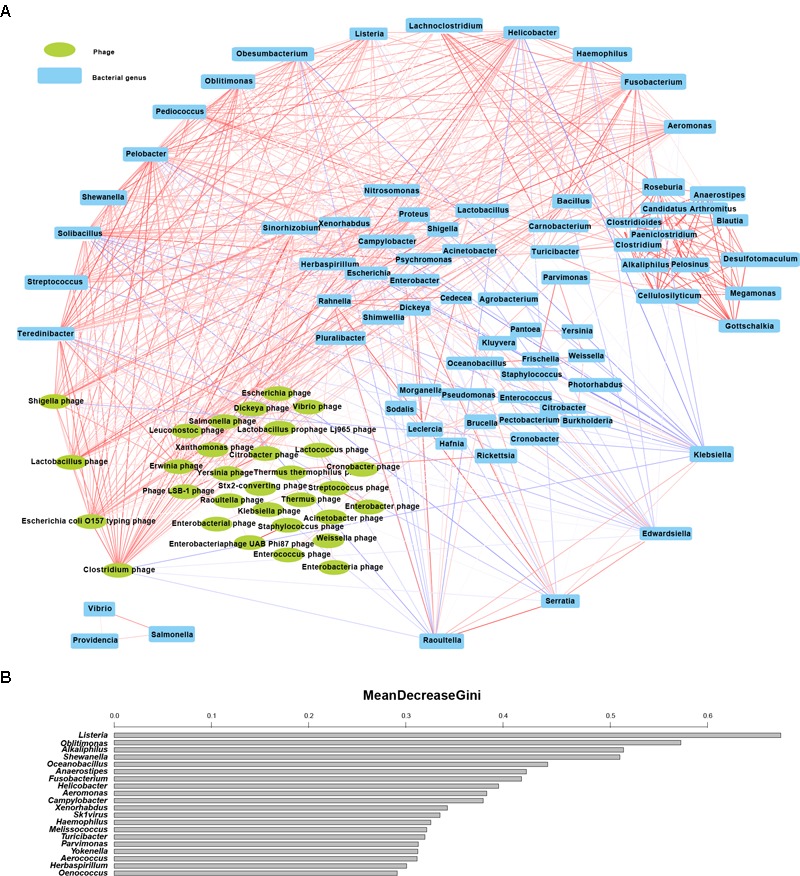
Discovery of important structural genera of gut microbiota with age. **(A)** Significant correlations (*t*-test, *p* < 0.05) in 112 genera of 52 stable microbial families (families with a presence rate above 70% in all samples) during cub development. The red and blue lines denote positive and negative correlations of the related genera, respectively; the thicker the line, the stronger are the correlations between paired genera. The uppermost genera have more than 30 links with other genera; genera at the bottom are those with numerous negative correlations with other genera; genera at the bottom left are bacteriophage groups; the rightmost area shows relatively independent groups with strong positive correlations. **(B)** The top 20 most important genera by random forest (RF) analysis (by age). The bar length shows the ability of microbes to decrease node impurity in the RF model. A large value means that the genus is very important for determining microbiota structure.

We further evaluated correlations among the 112 genera, and then removed non-significant correlations (*t*-test, *p* > 0.05). As shown in Figure 3A, four opportunistic pathogenic bacterial genera (*Klebsiella, Edwardsiella, Serratia*, and *Raoultella*), belonging to the order Enterobacterales, were negatively correlated with many other stable genera, and their abundances gradually decreased during the growth of giant panda cubs. Notably, 18 genera (at the upper outermost of Figure 3A, including 14 bacterial genera and 4 phage genera) have over 30 connections with other stable genera, indicating that they are located at the key ecological niches of the gut microbiota architecture of giant panda. Furthermore, by RF modeling (OOB error rate: 21.21%, the confusion errors occurred between C0 and C1 groups, or F and M groups), we obtained the top 20 most important microbial genera for determination of microbiota structure with age (Figure 3B). The integrated results of the two methods revealed that seven shared microbial genera are vital for determining microbiota structure (Table 1).

**Table 1:** Important microbial genera in the gut microbiota structure of giant panda in the 1st year life (the same genera are marked in bold).

Phylum	The 18 stable structural genera	The top 20 most important structural genera predicted by random forest model
Proteobacteria	***Shewanella*** ***Oblitimonas*** ***Helicobacter*** ***Haemophilus*** ***Aeromonas*** *Teredinibacter* *Pelobacter* *Obesumbacterium*	***Shewanella*** ***Oblitimonas*** ***Helicobacter*** ***Haemophilus*** ***Aeromonas*** *Campylobacter* *Xenorhabdus* *Yokenella* *Herbaspirillum*
Firmicutes	***Listeria*** *Streptococcus* *Solibacillus* *Pediococcus* *Lachnoclostridium*	***Listeria*** *Alkaliphilus* *Oceanobacillus* *Anaerostipes* *Melissococcus* *Turicibacter* *Parvimonas* *Aerococcus* *Oenococcus*
Fusobacteria	***Fusobacterium***	***Fusobacterium***
Viruses	*Clostridium phage* *Escherichia coli O157 typing phage* *Lactobacillus phage* *Shigella phage*	*Sk1virus* (*Lactococcus* virus)

### Gene Functions and Pathways of Intestinal Microbiome During the Growth of Cubs

**FIGURE 4:**
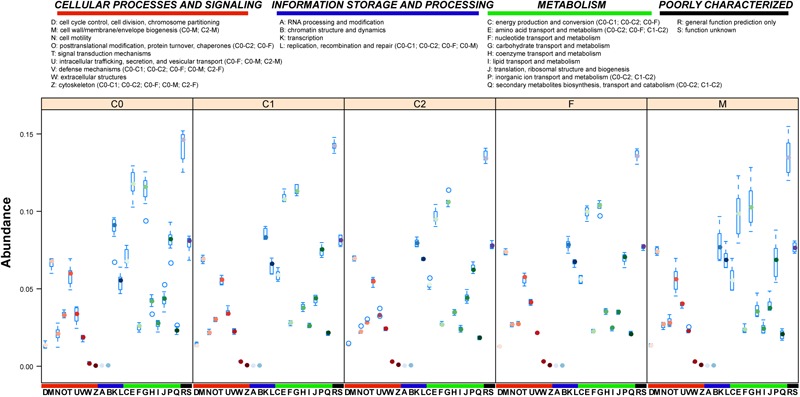
COG annotation of sample genes. COG functional annotation of gut microbial genes of investigated giant panda samples. The boxplot shows the gene abundance in COG functional classifications of samples by age. Significantly altered (Wilcoxon test, adjusted *p* < 0.05 by Holm method) COG classifications between age groups are shown in parentheses.

A total of 655,993 (63.04%) and 357,909 (34.39%) non-redundant genes were annotated in COG database and KEGG database, respectively. Only 10 orthologous functions showed significant changes (Wilcoxon test, adjusted *p* < 0.05) between age groups (Figure 4), in which nine belong to ‘cellular process and signaling’ or ‘metabolism,’ and the remaining one belongs to ‘information storage and processing.’ The four significant functions in ‘metabolism’ were seen mostly in the C0/C1 and C2/F/M groups. The five significant functions in ‘cellular process and signaling,’ and the one significant function in ‘information storage and processing,’ expressed more specific abundance profiles in C0 group samples than in other groups.

**FIGURE 5:**
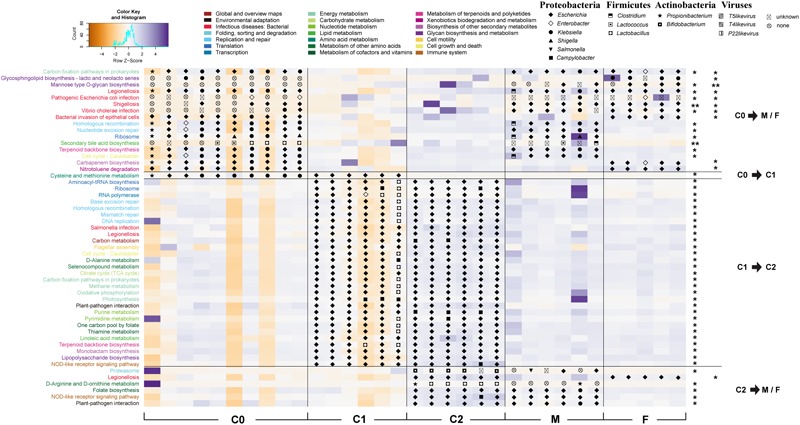
Significant gut microbial pathways of giant pandas with age. The heatmap profile shows scaled abundance (by row *z*-score) of significantly changed pathways in the gut of giant panda by age. Hosts of the pathways are marked on abundance patches. Pathways are colored according to KEGG pathway classifications, as in the figure legend. The significance of these pathways was evaluated by Wilcoxon test (*p*-values were adjusted by Holm method. ^∗^0.01 < *p* < 0.05; ^∗∗^*p* < 0.01).

A total of 167 pathways were detected in all samples by KEGG annotation, of which 44 showed significant changes (Wilcoxon test, adjusted *p* < 0.05) between age groups (Figure 5). Of the significantly changed pathways, 16 were seen in the transition from the C0 group to F/M groups: 5 infectious disease (in low abundance) pathways in the gut microbiota of cubs from *Escherichia, Enterobacter, Klebsiella*, or *Propionibacterium* were enriched in adult gut microbiota by *Escherichia, Enterobacter, Klebsiella*, or *Clostridium*; 3 genetic information-processing pathways, from *Escherichia, Enterobacter, Klebsiella*, or *Propionibacterium*, and *shigella* in the gut microbiota of cubs were enhanced in adult gut microbiota by *Escherichia, Klebsiella, Clostridium*, or *shigella*. In addition, we noticed that a ‘secondary bile acid biosynthesis’ pathway, involved in lipid metabolism and contributed to by *Lactobacillus* or *Lactococcus*, in low abundance in C0 group, was enriched in the F/M group by *Lactococcus* or *Clostridium*. Regarding C2 group, 29 pathways from *Escherichia, Campylobacter, Enterobacter*, or *Lactobacillus* were significantly enriched in the transition from the C1 to C2 group. Differing from the rare and relatively low abundant ‘energy metabolism and conversion’ pathways in C0 and C1 groups, 12 ‘energy metabolism and conversion’ pathways were significantly enriched in the C2 group, and this enrichment was accomplished mainly by *Escherichia*, followed by *Campylobacter*. Only six significantly changed pathways were detected between the C2 and F/M groups, and the abundance of these pathways was decreased in the F/M groups. Regarding the pathways ‘proteasome’ and ‘D-Arginine and D-ornithine metabolism,’ the hosts were changed from *Bifidobacterium* and *Lactobacillus* or *Propionibacterium* in the C2 group to *Salmonella* or *Escherichia* and *Propionibacterium* in the F/M groups, respectively.

### Diet-Related Enzymatic Transitions With Cub Age

To best explore the diet digestion ability of cubs, we investigated enzymatic changes in the gut microbiota of cubs with age. We annotated 2405 ECs in all samples, in which 603 ECs were significantly changed with age (Wilcoxon test, adjusted *p* < 0.05 by Holm method) (Supplementary Figures S4A and Table S5). Oxidoreductases, transferases and hydrolases showed the most significantly changed ECs between age groups (Supplementary Figure S4B). Consistent with earlier findings (Figures 1F, 5 and Supplementary Figure S3), very few significantly changed ECs between the C0 and C1 groups or M and F groups were detected. Furthermore, transferases harbored the most significantly changed ECs in the age groups, and abundant marked ECs occurred between the C0 and C2 groups (Supplementary Figure S4B).

**FIGURE 6:**
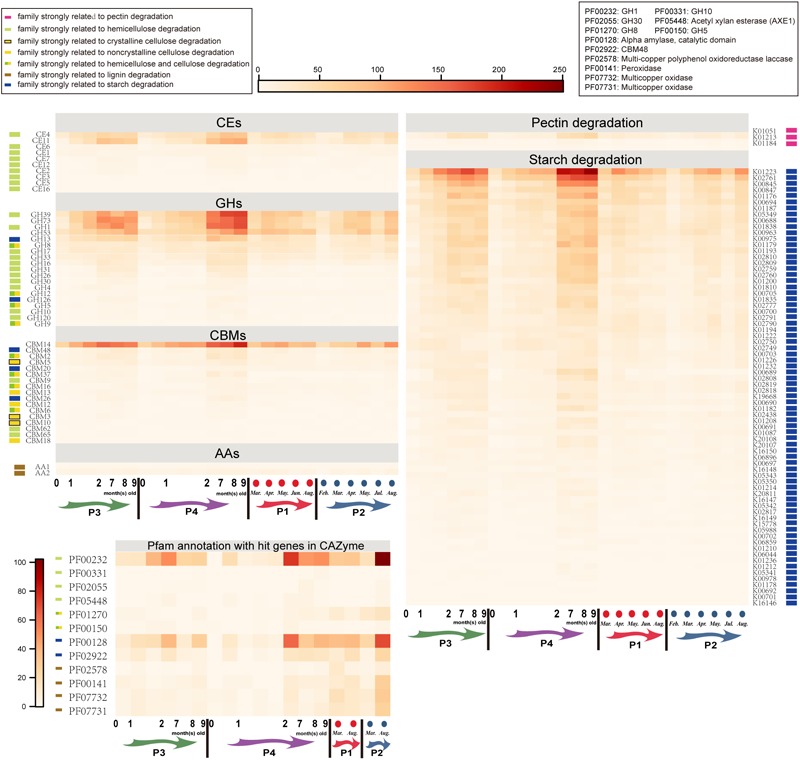
Abundance of microbial genes involved in bamboo digestion in the gut of cubs and their parents. Carbohydrate esterases (CEs), glycoside hydrolases (GHs), carbohydrate-binding modules (CBMs) and auxiliary activity (AAs) genes were annotated using the CAZy database; pectin degradation genes and starch degradation genes were annotated using the KEGG database. The genes matched in the CAZy database were further annotated in the Pfam database, to confirm the gene function, and only the abundances of bamboo-digesting genes were shown in the Pfam annotation results. P1: father; P2: mother; P3: elder cub; P4: younger cub.

Regarding the samples having the most paired reads (14 cub samples and 11 parental samples), we detected extensive increases of abundance of KOs relating to starch degradation from 2 months after birth, particularly at 6 ~ 9 months of age (Figure 6). Abundances of some starch-degrading genes in gut microbiota of 6 ~ 9-month-old cubs were even higher than in their parents (e.g., K01223: 6-phospho-beta-glucosidase; K02761: PTS system, cellobiose-specific IIC component). We also sought microbial genes related to the degradation of cellulose, hemicellulose, pectin and lignin in samples via KEGG and CAZy annotations. Results indicated that many of the detected genes also showed increases from 2 months after birth, and reached peak values at 6 ~ 9 months of age. To further confirm the profile, we re-annotated the hit genes in CAZy databases with pfam database annotation (Sukharnikov et al., 2011). Despite many hydrolases relating to the digestion of cellulose, hemicellulose (without xylosidase), pectin and lignin were missing or in very low abundance; pfam annotation supported the abundance in cubs profiled by CAZy annotation, but indicated higher abundance of hemicellulose/lignin-degrading genes in parents than in cubs. Regarding cellulase complex genes, we did not detect sequences of classic exo/endo-beta-1,4-glucanase (EC3.2.1.91 or EC3.2.1.4), and the abundances of cellulase glycoside hydrolases (GHs), such as GH5, GH8, GH9, GH12, were extremely low (Nguyen et al., 2018); xylanase (e.g., GH10, GH26, and GH30) and carbohydrate esterases (e.g., CE1, CE4, and CE6) that can digest the huge side chain of hemicellulose to expose the xylan backbone for xylanase hydrolysis were mostly present in low abundance (Nguyen et al., 2018). Interestingly, GH1 and GH39 accommodating many xylosidase were abundant, particularly in 6 ~ 9-month-old cubs. Furthermore, we monitored the abundance of carbohydrate-binding modules (CBMs) that have no enzymatic activity, but can guide neighbor enzymatic domain(s) to targeted carbohydrate substrates and disturb the stable surface of the carbohydrates, to promote carbohydrate degradation (Boraston et al., 2004). We detected involvement of CBM2, CBM3, CBM5, and CBM10 in crystalline cellulose degradation; CBM12, CBM13, CBM18 were involved in non-crystalline cellulose degradation; CBM2, CBM6, CBM16, and CBM37 were involved in Hemicellulose and non-crystalline cellulose degradation; CBM9, CBM62, CBM65 were involved in hemicellulose degradation; and CBM48, CBM20, CBM26 were involved in starch degradation (Boraston et al., 2004). However, similar to many GH families, these CBMs were consistently in very low abundance.

## Discussion

Although increasing numbers of studies of the significant gut microbes or pathways in giant panda have been reported in recent years (Xue et al., 2015; Wu et al., 2017; Guo et al., 2018; Yang et al., 2018; Zhang et al., 2018), few focused on giant panda cubs (Xue et al., 2015; Zhang et al., 2018) or established linkages between the significant gut microbes and pathways in cubs. Moreover, the two representative studies of cub gut microbiome omitted the significant microbiome changes in 6 ~ 9 months old of age during the nursing stage: one (Xue et al., 2015) focused on the gut microbiome of cubs (<6 months old), juvenile (2 ~ 5 years old) and adults (6 ~ 22 years old); the other one (Zhang et al., 2018) focused on cubs < 2 months old, cubs 3 ~ 12 months old, and juveniles (mostly >1 year old with bamboo diet). In addition, the samples allocated to each group were collected from different individuals. Individual differences could affect the gut microbiome of giant pandas, even in the same season (Xue et al., 2015). Thus, a consecutive and close study minimizing the factors except diet to trace gut microbiome transitions within the same giant panda cub after birth is urgently needed. Our study was based on the two newborn giant panda cubs and their parents living in Macao, China, to explore significant gut microbes and pathways, and their relevance in the growth of cubs and adults. We enriched microbes on a 0.22 μm membrane, then extracted their DNAs; obtained DNAs were deep sequenced with an average of 14.1 Gb clean data for each sample [~5.25 Gb each in Zhang et al. (2018), ~6 Gb each in Wu et al. (2017), and ~9.6 Gb in Yang et al. (2018)], and yielded a non-redundant gene set with 1,040,648 microbial (or bacterial and viral) genes [681,167 unique genes in Zhang et al. (2018)].

We detected an increase of gut bacterial richness with age, as per previous studies (Xue et al., 2015; Zhang et al., 2018). However, compared to 0 ~ 6-month-old cubs, the diversity decreased in the gut microbiota of 6 ~ 9-month-old cubs. One possible reason for this is that the dramatic expansion (Supplementary Figure S1 and Figures 1A,C) of gut microbiota after birth [particularly on cessation of breast milk (Backhed et al., 2015)] gave rise to increasing unevenness (Figure 1D) of gut microbiota. We detected dominant bacterial genera rarely reported in cubs or adults (Xue et al., 2015; Wu et al., 2017; Yang et al., 2018; Zhang et al., 2018), such as *Erwinia, Providencia, Edwardsiella, Pectobacterium*, and *Serratia*. Worthy of mention is *Pectobacterium*, a group of bacteria that produce pectolytic enzymes to hydrolyze pectins of bamboos. Meanwhile, the reported abundant genera *Leuconostoc* and *Stenotrophomonas* (Yang et al., 2018) were in low abundance in adults and all investigated samples, respectively. In addition, rapid enrichment of *Pediococcus, Lactobacillus*, and *Clostridium* in 3 ~ 12-month-old cubs (Zhang et al., 2018), and reductions of *Klebsiella* (Xue et al., 2015), *Campylobacter, Pediococcus*, and *Lactobacillus* (Zhang et al., 2018) in adults, were also observed in our study. We found that *Lactobacillus, Pediococcus*, and *Clostridium* started to become enriched from the 1st month after birth, and *Campylobacter* reached its highest abundance in 6 ~ 9-month-old cubs. These changes in *Pediococcus, Lactobacillus*, and *Campylobacter* made Lactobacillaceae and Campylobacteraceae the most significant changed microbial communities in the gut microbiota of 6 ~ 9-month-old cubs compared to both 0 ~ 1.5-month-old cubs and adults (Supplementary Figure S3). Besides, *Lactobacillus* and *Campylobacter* played significant metabolic roles in 6 ~ 9-month-old cubs: *Lactobacillus* largely promoted the ‘amino acid metabolism’ in 6 ~ 9-month-old cubs, especially ‘D-Arginine and D-ornithine metabolism.’ It also contributed to ‘secondary bile acid biosynthesis’ in 0 ~ 1.5-month-old cubs, and the low abundance of this pathway is a potential cause of the high content of undigested lipids seen during the DNA extractions. Fortunately, this pathway was later enriched by *Lactococcus* in adults (whereas *Lactobacillus* was largely decreased); *Campylobacter* significantly enriched ‘purine metabolism and pyrimidine metabolism,’ ‘carbon metabolism,’ ‘RNA polymerase,’ and the NOD-like receptor signaling pathway in 6 ~ 9-month-old cubs, which facilitated cell cycle and strengthened the environmental interplays of gut microbiota. Here, I stress that *Lactobacillus* and *Campylobacter* also enriched in gut microbiota of 1.5 ~ 6-month-old cubs, so they were excluded from the list of microbes that could discriminate the gut microbiota architecture among 0 ~ 1.5-month-old cubs (C0), 1.5 ~ 6-month-old cubs (C1), 6 ~ 9-month-old cubs (C2) and parents. However, Campylobacteraceae was reported to be scarce in >4-month-old captive pandas (Zhang et al., 2018). This can be understood by taking into consideration the different living conditions of (e.g., diet; region; captive, or wild) (Wu et al., 2017; Yang et al., 2018), and individual differences among (Xue et al., 2015), giant pandas.

In our study, the gut microbiota of 0 ~ 1.5-month-old cubs and 1.5 ~ 6-month-old cubs were strongly correlated (Figure 1F), while the maximum growth rate was seen at about 70 ~ 100 days, implying that the fast growth of cubs negatively affected the gut microbiota composition, and that longer interaction with the environment (e.g., diet) is needed for maturity of gut microbiota in the giant panda (Backhed et al., 2015). Here, we do not intend to deny the inheritability of entire gut microbial communities; a small group of microorganisms, especially pioneer species at birth, likely possess high inheritability potential (van Opstal and Bordenstein, 2015). However, the maturity of gut microbiota was largely accelerated after birth, when environmental factors (e.g., diet) intervened (Backhed et al., 2015); for example, when fed with solid food, a shift in gut microbial flora of cubs from *Lactobacillus* and *Bifidobacterium* to Enterobactereacea genera was seen (Hirayama et al., 2010). The ‘two-component system,’ ‘Protein export’ and ‘Bacterial secretion system’ pathways strongly relate to environmental interaction (Wu et al., 2017). We detected enrichment of these three pathways in the gut microbiome of 6 ~ 9-month-old cubs and adults, but they were in relatively lower abundance in 1.5 ~ 6-month-old cubs when compared to other stages, suggesting strengthened ability of environmental community with age. Similar abundance profiles were also observed in ‘Pyruvate metabolism,’ ‘Fatty acid metabolism,’ ‘Lysine metabolism,’ ‘Valine, leucine and isoleucine metabolism,’ ‘D-Glutamine and D-glutamate metabolism’ and ‘Alanine, aspartate and glutamate metabolism,’ regardless of their low abundances. These pathways showed higher abundances of genes catalyzing the degradation reactions than biosynthesis in the gut microbiome of adult giant panda (Guo et al., 2018). Moreover, we detected slightly higher abundances of ‘Fatty acid degradation’ than ‘Fatty acid biosynthesis,’ but lower abundance of genes taking part in the degradation of the amino acids, than biosynthesis with age.

In a previous study, 51 OTUs from *Megasphaera, Veillonella*, and *Clostridium*, and 26 MLGs from *Microbacterium, Clostridium, Citrobacter, Delftia, Enterobacter, Klebsiella, Raoultella, Serratia, Enterococcus, Lactobacillus*, and *Staphylococcus*, were identified as key variables for differentiation the microbial structure between <2-month-old cubs with breast milk diet and >1-year-old cubs with bamboo diet, and among <2-month-old cubs with breast milk diet, 3 ~ 12-month-old cubs with formula milk diet and 6-month-old juveniles with bamboo diet, respectively (Zhang et al., 2018). We found seven important genera that can best discriminate the gut microbial profiles of 0 ~ 1.5-months-old cubs, 1.5 ~ 6-month-old cubs, 6 ~ 9-month-old cubs and adults: *Shewanella, Helicobacter, Oblitimonas, Haemophilus, Aeromonas, Listeria*, and *Fusobacterium*. Many species of *Shewanella* have been reported to reduce metals and toxins (Harris et al., 2017); the other six genera *Helicobacter* (Fischbach and Malfertheiner, 2018), *Oblitimonas* (Drobish et al., 2016), *Haemophilus* (Bakaletz and Novotny, 2018), *Aeromonas* (Cardoso et al., 2018), *Listeria* (Salama et al., 2018), and *Fusobacterium* (Guven and Dizdar, 2018) have been implicated in human diseases. *Helicobacter* was even expressed in higher abundance in 6 ~ 9-month-old cubs than in adults, suggesting that giant panda would be more susceptible to digestive diseases in this stage. *Listeria* (*Listeria ivanovii, L*. *monocytogenes, L*. *welshimeri*, and *L*. *seeligeri*), rarely reported in previous studies (Xue et al., 2015; Wu et al., 2017; Zhang et al., 2018), was the only important genera with low abundance in the gut microbiome of cubs and adults. The *Listeria* species detected in age groups except 6 ~ 9 months old were non-pathogenic *L*. *welshimeri* and *L*. *seeligeri*, rather than pathogenic *L*. *ivanovii* and *L*. *monocytogenes*. The finding that pathogenic *L*. *ivanovii* and *L*. *monocytogenes* appeared in the gut of 6 ~ 9-month-old cubs reflects the disadvantages of dramatic gut biomass expansion after birth. Therefore, subsequent adjustments of early life gut microbiome along with adult gut metabiome are crucial for giant panda health. At 1 year of age, the gut microbiome of giant panda cub shows many similar functional attributes to that of adults (Zhang et al., 2018); 2-year-old juveniles exhibited similar gut microbiota diversity to adults (Xue et al., 2015). The significances of these (opportunistic) pathogens suggest that they play important ecological role beyond invasion in the gut ecosystem of giant panda in evolution: in our study, we detected that these pathogens were widely involved in ‘cell cycle,’ ‘metabolism,’ and ‘environmental information processing,’ despite little significances; in another metagenomic study (Yang et al., 2018), *Serratia, Bacillus*, and *Pseudomonas* were found participating cellulose degradation. However, significant increases of many pathogens enriched infectious pathway abundance (Figure 5) with age. We found that five infectious pathways implicated in invasions of *Escherichia, Shigella*, or *Vibrio* were significantly enriched in adults. Legionellosis (contributed mainly by *Escherichia coli*) was the only infectious pathway that significantly changed with age. Taken together with the enrichment of (opportunistic) pathogens and infectious pathways and large decreases of anti-pathogenic *Bifidobacterium* and *Lactobacillus* (Liu et al., 2017), it is intuitive that adult giant panda experiences digestive diseases (e.g., diarrhea) every year.

*Escherichia* and *Klebsiella*, but especially *Escherichia*, contributed most to the metabolic pathways in the age groups in our study. *Klebsiella* participated in many metabolic pathways in 0 ~ 1.5-month-old cubs, before being replaced by *Escherichia*. We detected no enrichment of pyruvate or glycerolipid metabolism in 0 ~ 2-month-old cubs compared with adults (Zhang et al., 2018). Instead, 16 pathways in the gut microbiome of 0 ~ 1.5-month-old cubs were significantly enriched in adults, most of them involved in infection, the cell cycle and glycan metabolism. Moreover, enrichment of the purine biosynthesis pathway and genes involved in cellular processes was observed in 6 ~ 9-month-old cubs when compared with 0 ~ 2-month-old cubs. Enrichment was also found in >1-year old cubs (Zhang et al., 2018).

Bamboo diet selects for fiber-digesting microbes (McKenney et al., 2018). In this study, we noted a similar phenomenon in the early life of giant panda under breast or formula feeding. *Lactobacillus* and *Bifidobacterium* were enriched during breast or formula feeding, yet sharply decreased in adults. *Bifidobacterium* significantly promoted the pathway ‘proteasome’ in 6 ~ 9-month-old cubs, aiding proteolytic degradation and maintaining stability of cells (Wang and Maldonado, 2006), which expanded the gut microbiota defense system of giant panda (Hall-Stoodley et al., 2004; Zhou et al., 2018). Besides, *Bifidobacterium* was reported to take part in cellulose degradation (Yang et al., 2018). The dramatic decreases of *Lactobacillus* and *Bifidobacterium* in adult giant panda would likely impair the nutrition conversion and stability of the gut microbiota; *Clostridium* was regarded to be the only genera relating to giant panda age (Zhang et al., 2018) and was involved in cellulose and hemicellulose degradation (Xue et al., 2015). *Clostridium* was enriched in the gut microbiota of 6 ~ 9-month-old cubs, when the cubs were under formula feeding, suggesting that it has diverse roles: in a previous study (Yang et al., 2018), the major contributors to cellulase were the bacterial genera *Bacillus, Thermoanaerobacter*, and *Pseudomonas* or the fungal genera *Aspergillus* and *Bipolaris*, rather than *Clostridium* or Bacteroidetes genera; in our study, *Clostridium* significantly increased the abundance of the ‘Legionellosis,’ ‘Homologous recombination,’ ‘Ribosome,’ and ‘Cell cycle - Caulobacter’ pathways in adults.

Regarding the bamboo digestion, low abundances of genes relating to the degradation of cellulose, hemicellulose xylan backbone, pectin and lignin were seen in our samples, as reported in similar studies (Zhu et al., 2011; Guo et al., 2018; McKenney et al., 2018; Zhang et al., 2018). However, a recent study of gut microbiome of captive, semiwild and wild giant pandas (adult) detected 1739 genes homologous to cellulase, β-glucosidase and cellulose 1,4-β-cellobiosidase (Yang et al., 2018), demonstrating a high potential of giant panda gut microbiome with respect to cellulose digestion. Furthermore, another study detected a higher abundance of hemicellulose-degrading genes (e.g., CEs and GH4, 8, 31) in juvenile giant pandas (>1 year old) than in giant panda cubs (0 ~ 12 months old), omnivores and carnivores. In contrast, low abundance of cellulose and hemicellulose enzymes (e.g., GH5, 28, 9, 10, 26) was reported elsewhere (Zhu et al., 2011; Guo et al., 2018). And, an experimental assay of cellulase and xylanase activity in fecal samples demonstrated low-level activity of these enzymes as carnivores (Guo et al., 2018). In our study, we detected low abundance of genes of GH4, 8, 31, 5, 9, 10, 26.

We noticed that 6 ~ 9-month-old cubs possessed a higher abundance of GH families. This may also have been caused by the dramatic explosion of gut biomass after birth (particularly at 6 months after birth), which led to a fast increase of gut microbial richness and abundance (Figure 1A and Supplementary Figure S1). Despite the fact that proteobacteria (e.g., Enterobacteriaceae) started to reduce, while Firmicutes (e.g., *Clostridium*) was enriched, after 12 months old of age (Zhang et al., 2018), the biomass in the gut of 6 ~ 9-month-old cubs may already be present at high levels (particularly Firmicutes and Enterobacteriaceae). The huge biomass and high richness would contribute to higher abundance of GH families in 6 ~ 9-month-old cubs than in other developmental phases. Besides, we found abundant genes of GH1 and GH39 that accommodate many xylosidases in 6 ~ 9-month-old cubs and adults. This was further supported by Pfam annotation (abundant α-amylase and GH1) and similar study (most abundant GH families: GH13, GH23, GH3, GH1, and GH2) (Yang et al., 2018). This suggests that xylosidase acts positively in bamboo (hemicellulose) digestion. Apart from low abundance of many digestive enzymes, huge spatial barriers between enzymes and substrates also restrict the digestion of bamboos. In the bamboo cell wall, hemicellulose, cellulose and lignin were joined by covalent bonds (hemicellulose and lignin) or hydrogen bonds (intracellulose, cellulose and lignin, hemicellulose and lignin). Thus, massive lignins, which are much harder to degrade in the gut of giant panda, firmly embed celluloses and hemicelluloses, which greatly impedes the approach of carbohydrate enzymes to cellulose or hemicellulose substrates. Fortunately, 12 significantly promoted energy metabolism pathways which were mainly contributed to by *Escherichia* and abundant pectolytic *Pectobacterium*, would aid low utilization of bamboo celluloses in the gut microbiome of adult giant panda.

Last but by no means least, bacteriophages closely relate to bacterial microbiome in gut ecosystems in early life (Lim et al., 2015). We harvested the bacteriophages inside bacteria on a 0.22 μm membrane, which would miss many bacteriophages with a smaller size. However, we discovered those bacteriophages closely related to bacteria. Consistent with a study of bacteriophage diversity in the gut microbiota of adult giant pandas (Yang et al., 2018), we found that Caudovirales were dominant, indicating that tailed bacteriophages play important roles in regulating the gut bacterial microbiome of giant panda. Further, we detected Caudovirales genera *P22virus, Lambdavirus, P1virus, P2virus*, and *T4virus* in high abundance in cubs and adult giant pandas, whereas some abundant genera, namely *Epsilon15 virus, Nona33 virus, Rtp virus, G7c virus*, and *Tl2011 virus* (Yang et al., 2018), were not detected in our study. Meanwhile, we observed that *Muvirus, Sk1virus, Sfi21dt1virus*, and *Sfi11virus* were enriched after cubs began formula feeding. Members of *Muvirus* mainly infect *Escherichia*; *Shigella*; *Sk1virus, Sfi21dt1virus*, and *Sfi11virus* infect *Lactococcus* or *Streptococcus*. Besides, the major hosts of bacteriophages were *Escherichia*, followed by *Enterobacteria, Salmonella, Shigella*, and *Lactococcus* in the gut microbiota of adult giant pandas in a previous study (Yang et al., 2018). By contrast, in our study, the major hosts in adults were *Enterobacteria*, followed by *Salmonella, Escherichia, Klebsiella, Lactococcus*; in cubs were *Enterobacteria*, followed by *Escherichia, Salmonella, Lactococcus*, and *Streptococcus*. All involved bacteria were dominant in samples. A study on Canine distemper virus, an ssRNA virus of genus *Morbillivirus*, demonstrated that it induced death in giant panda by reducing the richness of intestinal *Escherichia* and *Clostridium*, and increasing the diversity of gut microbes, to finally break the structure of intestinal microbes of giant panda (Zhao et al., 2017b). Thus, a detailed study on the relationship between virome and bacterial microbiome in the gut of giant panda is urgently needed in future.

## Conclusion

Gut microbial transitions, inheritability and diet are important issues in policies for protecting giant panda. This study showed gradually increased richness, but reduced inheritance, of gut microbiota during the growth of giant panda cubs after birth, and the vital role of Enterobacteriaceae members in the gut microbiota of giant panda cubs. Cubs at 6 months old enter a significant period, during which a comprehensive and substantial increase of microbiome occurs. In addition, rapid enrichment of diet-related bacteria or phages was observed in the gut microbiota of cubs under breast and formula feeding, yet rapidly decreased in their parents, which indicated diet-stimulated gut microbiome transitions in the gut of giant panda in early life.

## Availability of Data and Materials

All raw reads of samples are available in National Center for Biotechnology Information (NCBI) database, and have been deposited under BioProject ID: PRJNA477424. The SRA accession number is SRP154209. The data reported in this study are also available in the CNGB Nucleotide Sequence Archive (CNSA: https://db.cngb.org/cnsa; accession number CNP0000135).

## Ethics Statement

The sample collection and all the experiments were performed in a manner to minimize risk to the giant pandas and the environment. All experimental protocols of this study were approved by Institute of Chinese Medical Sciences - Animal Ethics Committee (ICMS-AEC) of the University of Macau.

## Author Contributions

MG and SL designed the research. MG and JwC performed the research. MG, JwC, JM, YW, and GF contributed to analytic tools. MG, JC, YF, QL, LP, and LZ analyzed data. MG and SL wrote the paper. All authors read and approved the final manuscript.

## Conflict of Interest Statement

LZ and JC were employed by company Realbio Technology Co., Ltd. The remaining authors declare that the research was conducted in the absence of any commercial or financial relationships that could be construed as a potential conflict of interest.

## Supplementary Material

FIGURE S1 Gut microbial transitions of giant panda cubs and parents. The figure demonstrates the gut microbial family compositions of giant pandas. The phylogenetic tree on the left shows the evolutionary relationships of these families, and the colors on the heatmap indicate the relative abundance of the assigned genes. The microbial profiles of each cub were arranged by age, and the microbial profiles of parents by month. P1: father; P2: mother; P3: the elder cub; P4: the younger cub.

FIGURE S2 Daily weight of the two cubs from birth to 300 days old. The flag (at 48 days old) shows the diet transition time. Green and purple dots indicate the daily weights of elder cub (P3) and younger cub, respectively. P3: elder cub; P4: younger cub.

FIGURE S3 Gut microbial biomarkers during the development of giant panda cubs and parents. Microbial taxa with an LDA score higher than 4.0 were thought to be the representative communities of significantly changed (in abundance) microbial communities between the two microbiota groups. Circular phylogenetic trees show the relationships among biomarker taxa. **(A)** 0 ~ 1.5-month-old cubs versus 1.5 ~ 6-month-old cubs. **(B)** 1.5 ~ 6-month-old cubs versus 6 ~ 9-month-old cubs. **(C)** 6 ~ 9-month-old cubs versus father. **(D)** 6 ~ 9-month-old cubs versus mother.

FIGURE S4 Enzymatic transitions in gut microbiota of giant pandas with age. **(A)** Significant (Wilcoxon test, adjusted *P* < 0.05 by Holm method) gut microbial ECs between age groups of giant pandas. All 603 ECs were clustered by the six enzymatic classifications and colored accordingly. The circle size of the node indicates the sum of significant ECs between the labeled two age groups. **(B)** Significantly different gut microbial EC counts between age groups of giant pandas.

TABLE S1 Filtration of reads with low sequencing quality, reads polluted by adapter sequences and reads from giant panda or bamboo genome.

TABLE S2 Assembly of high-quality microbial reads of samples.

TABLE S3 Statistics of genes in non-redundant gene sets and samples.

TABLE S4 Abundance of gut microbial genera at the birth (~1 day) of giant panda cubs.

TABLE S5 Significantly changed ECs with age (C0: 0 ~ 1.5-month-old cubs; C1: 1.5 ~ 6-month-old cubs; C2: 6 ~ 9-month-old cubs; F, father; M, mother).

## References

[B1] Ackermann H. W. (2003). Bacteriophage observations and evolution. *Res. Microbiol.* 154 245–251. 10.1016/S0923-2508(03)00067-6 12798228

[B2] Altschul S. F. Gish W. Miller W. Myers E. W. Lipman D. J. (1990). Basic local alignment search tool. *J. Mol. Biol.* 215 403–410. 10.1016/S0022-2836(05)80360-2 2231712

[B3] Backhed F. Roswall J. Peng Y. Feng Q. Jia H. Kovatcheva-Datchary P. (2015). Dynamics and stabilization of the human gut microbiome during the first year of life. *Cell Host Microbe* 17 690–703. 10.1016/j.chom.2015.04.004 25974306

[B4] Bakaletz L. O. Novotny L. A. (2018). Nontypeable *Haemophilus influenzae* (NTHi). *Trends Microbiol.* 26 727–728. 10.1016/j.tim.2018.05.001 29793827

[B5] Boraston A. B. Bolam D. N. Gilbert H. J. Davies G. J. (2004). Carbohydrate-binding modules: fine-tuning polysaccharide recognition. *Biochem. J.* 382(Pt 3) 769–781. 10.1042/BJ20040892 15214846 PMC1133952

[B6] Camacho C. Coulouris G. Avagyan V. Ma N. Papadopoulos J. Bealer K. (2009). BLAST+: architecture and applications. *BMC Bioinformatics* 10:421. 10.1186/1471-2105-10-421 20003500 PMC2803857

[B7] Cardoso M. D. Lemos L. S. Roges E. M. de Moura J. F. Tavares D. C. Matias C. A. R. (2018). A comprehensive survey of *Aeromonas* sp. and *Vibrio* sp. in seabirds from Southeastern Brazil: outcomes for public health. *J. Appl. Microbiol.* 124 1283–1293. 10.1111/jam.13705 29356247

[B8] Che T. D. Wang C. D. Jin L. Wei M. Wu K. Zhang Y. H. (2015). Estimation of the growth curve and heritability of the growth rate for giant panda (*Ailuropoda melanoleuca*) cubs. *Genet. Mol. Res.* 14 2322–2330. 10.4238/2015.March.27.17 25867378

[B9] Chen Y. Chen Y. Shi C. Huang Z. Zhang Y. Li S. (2018). SOAPnuke: a MapReduce acceleration-supported software for integrated quality control and preprocessing of high-throughput sequencing data. *Gigascience* 7 1–6. 10.1093/gigascience/gix120 29220494 PMC5788068

[B10] Dierenfeld E. S. Hintz H. F. Robertson J. B. Van Soest P. J. Oftedal O. T. (1982). Utilization of bamboo by the giant panda. *J. Nutr.* 112 636–641. 10.1093/jn/112.4.636 6279804

[B11] Dray S. Dufour A.-B. (2007). The ade4 Package: implementing the duality diagram for ecologists. *J. Stat. Softw.* 22:20 10.18637/jss.v022.i04

[B12] Drobish A. M. Emery B. D. Whitney A. M. Lauer A. C. Metcalfe M. G. Mcquiston J. R. (2016). *Oblitimonas alkaliphila* gen. nov., sp. nov., in the family Pseudomonadaceae, recovered from a historical collection of previously unidentified clinical strains. *Int. J. Syst. Evol. Microbiol.* 66 3063–3070. 10.1099/ijsem.0.001147 27169721 PMC5653923

[B13] Fischbach W. Malfertheiner P. (2018). *Helicobacter pylori* infection. *Dtsch. Arztebl. Int.* 115 429–436. 10.3238/arztebl.2018.0429 29999489 PMC6056709

[B14] Forslund K. Hildebrand F. Nielsen T. Falony G. Le Chatelier E. Sunagawa S. (2015). Disentangling type 2 diabetes and metformin treatment signatures in the human gut microbiota. *Nature* 528 262–266. 10.1038/nature15766 26633628 PMC4681099

[B15] Fu G. Saunders G. Stevens J. (2014). Holm multiple correction for large-scale gene-shape association mapping. *BMC Genet.* 15(Suppl. 1):S5. 10.1186/1471-2156-15-S1-S5 25079623 PMC4118635

[B16] Galperin M. Y. Makarova K. S. Wolf Y. I. Koonin E. V. (2015). Expanded microbial genome coverage and improved protein family annotation in the COG database. *Nucleic Acids Res.* 43 D261–D269. 10.1093/nar/gku1223 25428365 PMC4383993

[B17] Guo W. Mishra S. Zhao J. Tang J. Zeng B. Kong F. (2018). Metagenomic study suggests that the gut microbiota of the giant panda (*Ailuropoda melanoleuca*) may not be specialized for fiber fermentation. *Front. Microbiol.* 9:229. 10.3389/fmicb.2018.00229 29503636 PMC5820910

[B18] Guven D. C. Dizdar O. (2018). *Fusobacterium* and colorectal carcinogenesis. *Carcinogenesis* 39:84 10.1093/carcin/bgx092 28968715

[B19] Hall-Stoodley L. Costerton J. W. Stoodley P. (2004). Bacterial biofilms: from the natural environment to infectious diseases. *Nat. Rev. Microbiol.* 2 95–108. 10.1038/nrmicro821 15040259

[B20] Harris H. W. Sanchez-Andrea I. McLean J. S. Salas E. C. Tran W. El-Naggar M. Y. (2017). Redox sensing within the genus *Shewanella*. *Front. Microbiol.* 8:2568 10.3389/fmicb.2017.02568 PMC5789149 29422884

[B21] Hirayama K. Kawamura S. Mitsuoka T. Tashiro K. (2010). The faecal flora of the giant panda (*Ailuropoda melanoleuca*). *J. Appl. Bacteriol.* 67 411–415. 10.1111/j.1365-2672.1989.tb02511.x 2584169

[B22] Hong C. Manimaran S. Shen Y. Perez-Rogers J. F. Byrd A. L. Castro-Nallar E. (2014). PathoScope 2.0: a complete computational framework for strain identification in environmental or clinical sequencing samples. *Microbiome* 2:33. 10.1186/2049-2618-2-33 25225611 PMC4164323

[B23] Jie Z. Xia H. Zhong S. L. Feng Q. Li S. Liang S. (2017). The gut microbiome in atherosclerotic cardiovascular disease. *Nat. Commun.* 8:845. 10.1038/s41467-017-00900-1 29018189 PMC5635030

[B24] Kanehisa M. Sato Y. Kawashima M. Furumichi M. Tanabe M. (2016). KEGG as a reference resource for gene and protein annotation. *Nucleic Acids Res.* 44 D457–D462. 10.1093/nar/gkv1070 26476454 PMC4702792

[B25] Langmead B. Salzberg S. L. (2012). Fast gapped-read alignment with Bowtie 2. *Nat. Methods* 9 357–359. 10.1038/nmeth.1923 22388286 PMC3322381

[B26] Li W. Godzik A. (2006). Cd-hit: a fast program for clustering and comparing large sets of protein or nucleotide sequences. *Bioinformatics* 22 1658–1659. 10.1093/bioinformatics/btl158 16731699

[B27] Lim E. S. Zhou Y. Zhao G. Bauer I. K. Droit L. Ndao I. M. (2015). Early life dynamics of the human gut virome and bacterial microbiome in infants. *Nat. Med.* 21 1228–1234. 10.1038/nm.3950 26366711 PMC4710368

[B28] Liu Q. Ni X. Wang Q. Peng Z. Niu L. Wang H. (2017). *Lactobacillus plantarum* BSGP201683 isolated from giant panda feces attenuated inflammation and improved gut microflora in mice challenged with enterotoxigenic *Escherichia coli*. *Front. Microbiol.* 8:1885. 10.3389/fmicb.2017.01885 29018435 PMC5623042

[B29] Ma J. Wang C. Long K. Zhang H. Zhang J. Jin L. (2017). Exosomal microRNAs in giant panda (*Ailuropoda melanoleuca*) breast milk: potential maternal regulators for the development of newborn cubs. *Sci. Rep.* 7:3507. 10.1038/s41598-017-03707-8 28615713 PMC5471263

[B30] McKenney E. A. Maslanka M. Rodrigo A. Yoder A. D. (2018). Bamboo specialists from two mammalian orders (Primates, Carnivora) share a high number of low-abundance gut microbes. *Microb. Ecol.* 76 272–284. 10.1007/s00248-017-1114-8 29188302

[B31] Nguyen S. T. C. Freund H. L. Kasanjian J. Berlemont R. (2018). Function, distribution, and annotation of characterized cellulases, xylanases, and chitinases from CAZy. *Appl. Microbiol. Biotechnol.* 102 1629–1637. 10.1007/s00253-018-8778-y 29359269 PMC5806127

[B32] Nielsen H. B. Almeida M. Juncker A. S. Rasmussen S. Li J. Sunagawa S. (2014). Identification and assembly of genomes and genetic elements in complex metagenomic samples without using reference genomes. *Nat. Biotechnol.* 32 822–828. 10.1038/nbt.2939 24997787

[B33] Peng Y. Leung H. C. Yiu S. M. Chin F. Y. (2012). IDBA-UD: a de novo assembler for single-cell and metagenomic sequencing data with highly uneven depth. *Bioinformatics* 28 1420–1428. 10.1093/bioinformatics/bts174 22495754

[B34] Pielou E. C. (1969). An introduction to mathematical ecology. *Bioscience* 24 7–12.

[B35] Rothschild D. Weissbrod O. Barkan E. Kurilshikov A. Korem T. Zeevi D. (2018). Environment dominates over host genetics in shaping human gut microbiota. *Nature* 555 210–215. 10.1038/nature25973 29489753

[B36] Salama P. J. Embarek P. K. B. Bagaria J. Fall I. S. (2018). Learning from listeria: safer food for all. *Lancet* 391 2305–2306. 10.1016/S0140-6736(18)31206-6 29900862

[B37] Segata N. Izard J. Waldron L. Gevers D. Miropolsky L. Garrett W. S. (2011). Metagenomic biomarker discovery and explanation. *Genome Biol.* 12:R60. 10.1186/gb-2011-12-6-r60 21702898 PMC3218848

[B38] Shannon C. E. A. (2001). A mathematical theory of communication. AT&T Tech J. *ACM Sigmobile Mobile Comput. Commun. Rev.* 5 3–55. 10.1145/584091.584093

[B39] Shannon P. Markiel A. Ozier O. Baliga N. S. Wang J. T. Ramage D. (2003). Cytoscape: a software environment for integrated models of biomolecular interaction networks. *Genome Res.* 13 2498–2504. 10.1101/gr.1239303 14597658 PMC403769

[B40] Sukharnikov L. O. Cantwell B. J. Podar M. Zhulin I. B. (2011). Cellulases: ambiguous nonhomologous enzymes in a genomic perspective. *Trends Biotechnol.* 29 473–479. 10.1016/j.tibtech.2011.04.008 21683463 PMC4313881

[B41] van Opstal E. J. Bordenstein S. R. (2015). Microbiome. Rethinking heritability of the microbiome. *Science* 349 1172–1173. 10.1126/science.aab3958 26359393

[B42] Wang J. Maldonado M. A. (2006). The ubiquitin-proteasome system and its role in inflammatory and autoimmune diseases. *Cell Mol. Immunol.* 3 255–261. 16978533

[B43] Warnes G. R. Bolker B. Bonebakker L. Gentleman R. Huber W. Liaw A. (2014). *gplots: Various R Programming Tools for Plotting Data. R Package Version 2.17.0.*

[B44] Wu Q. Wang X. Ding Y. Hu Y. Nie Y. Wei W. (2017). Seasonal variation in nutrient utilization shapes gut microbiome structure and function in wild giant pandas. *Proc. Biol. Sci.* 284:1862. 10.1098/rspb.2017.0955 28904137 PMC5597826

[B45] Xue Z. Zhang W. Wang L. Hou R. Zhang M. Fei L. (2015). The bamboo-eating giant panda harbors a carnivore-like gut microbiota, with excessive seasonal variations. *mBio* 6:e00022-15. 10.1128/mBio.00022-15 25991678 PMC4442137

[B46] Yang S. Gao X. Meng J. Zhang A. Zhou Y. Long M. (2018). Metagenomic analysis of bacteria, fungi, bacteriophages, and helminths in the gut of giant pandas. *Front. Microbiol.* 9:1717. 10.3389/fmicb.2018.01717 30108570 PMC6080571

[B47] Zaharia M. Bolosky W. J. Curtis K. Fox A. Patterson D. Shenker S. (2011). Faster and more accurate sequence alignment with SNAP. arXiv:1111.5572. Available at: http://snap.cs.berkeley.edu/

[B48] Zhang T. Zhang R. Zhang L. Zhang Z. Hou R. Wang H. (2015). Changes in the milk metabolome of the giant panda (*Ailuropoda melanoleuca*) with time after birth–three phases in early lactation and progressive individual differences. *PLoS One* 10:e0143417. 10.1371/journal.pone.0143417 26630345 PMC4668050

[B49] Zhang W. Liu W. Hou R. Zhang L. Schmitz-Esser S. Sun H. (2018). Age-associated microbiome shows the giant panda lives on hemicelluloses, not on cellulose. *ISME J.* 12 1319–1328. 10.1038/s41396-018-0051-y 29391488 PMC5931968

[B50] Zhang W. Yang S. Shan T. Hou R. Liu Z. Li W. (2017). Virome comparisons in wild-diseased and healthy captive giant pandas. *Microbiome* 5:90. 10.1186/s40168-017-0308-0 28780905 PMC5545856

[B51] Zhao N. Li M. Luo J. Wang S. Liu S. Lyu W. (2017a). Impacts of canine distemper virus infection on the giant panda population from the perspective of gut microbiota. *Sci. Rep.* 7:39954. 10.1038/srep39954 28051146 PMC5209704

[B52] Zhao N. Li M. Luo J. Wang S. Liu S. Wang S. (2017b). Impacts of canine distemper virus infection on the giant panda population from the perspective of gut microbiota. *Sci. Rep.* 7:39954. 10.1038/srep39954 28051146 PMC5209704

[B53] Zhou Z. Liu F. Zhang X. Zhou X. Zhong Z. Su H. (2018). Cellulose-dependent expression and antibacterial characteristics of surfactin from *Bacillus subtilis* HH2 isolated from the giant panda. *PLoS One* 13:e0191991. 10.1371/journal.pone.0191991 29385201 PMC5791997

[B54] Zhou Z. Zhou X. Li J. Zhong Z. Li W. Liu X. (2015). Transcriptional regulation and adaptation to a high-fiber environment in *Bacillus subtilis* HH2 isolated from feces of the giant panda. *PLoS One* 10:e0116935. 10.1371/journal.pone.0116935 25658435 PMC4319723

[B55] Zhu L. Wu Q. Dai J. Zhang S. Wei F. (2011). Evidence of cellulose metabolism by the giant panda gut microbiome. *Proc. Natl. Acad. Sci. U.S.A.* 108 17714–17719. 10.1073/pnas.1017956108 22006317 PMC3203778

[B56] Zhu W. Lomsadze A. Borodovsky M. (2010). Ab initio gene identification in metagenomic sequences. *Nucleic Acids Res.* 38:e132. 10.1093/nar/gkq275 20403810 PMC2896542

[B57] Zuur A. F. Ieno E. N. Meesters E. (2009). *An Introduction to the Lattice Package.* Berlin: Springer International Publishing 10.1007/978-0-387-93837-0_8

