# Supplementary figures and images for: Dynamics of Gut Microbiome in Giant Panda Cubs Reveal Transitional Microbes and Pathways in Early Life
Source: Front Microbiol. 2018 Dec 18;9:3138. doi: 10.3389/fmicb.2018.03138 (PMC6305432; doi:10.3389/fmicb.2018.03138)

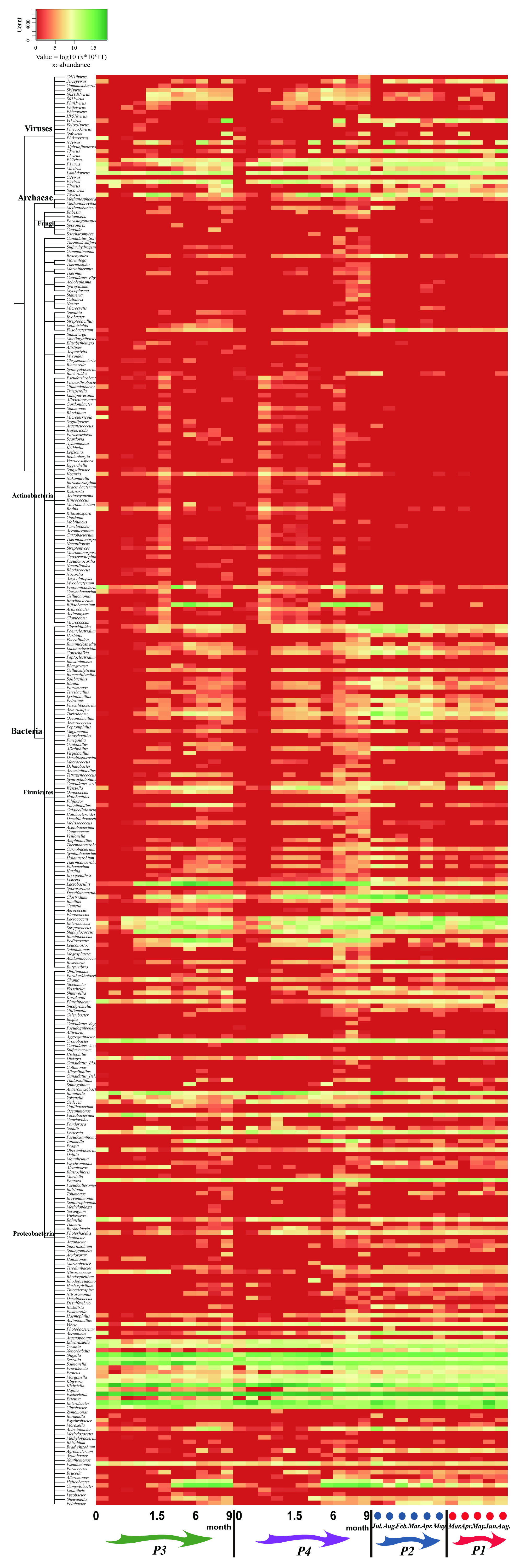

Supplement: FIGURE S1 — Gut microbial transitions of giant panda cubs and parents. The figure demonstrates the gut microbial family compositions of giant pandas. The phylogenetic tree on the left shows the evolutionary relationships of these families, and the colors on the heatmap indicate the relative abundance of the assigned genes. The microbial profiles of each cub were arranged by age, and the microbial profiles of parents by month. P1: father; P2: mother; P3: the elder cub; P4: the younger cub. [file Image_1.TIF]

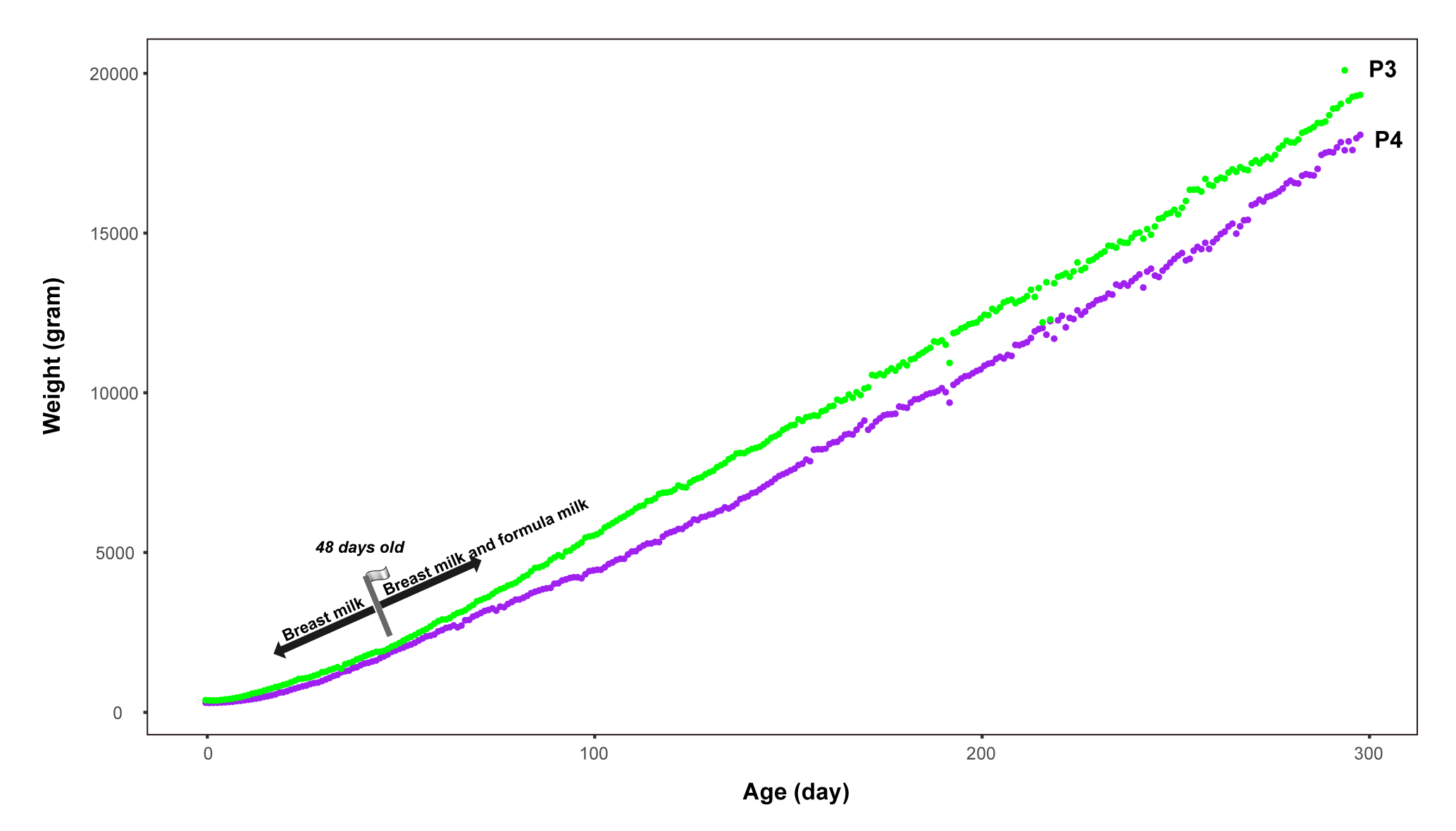

Supplement: FIGURE S2 — Daily weight of the two cubs from birth to 300 days old. The flag (at 48 days old) shows the diet transition time. Green and purple dots indicate the daily weights of elder cub (P3) and younger cub, respectively. P3: elder cub; P4: younger cub. [file Image_2.TIF]

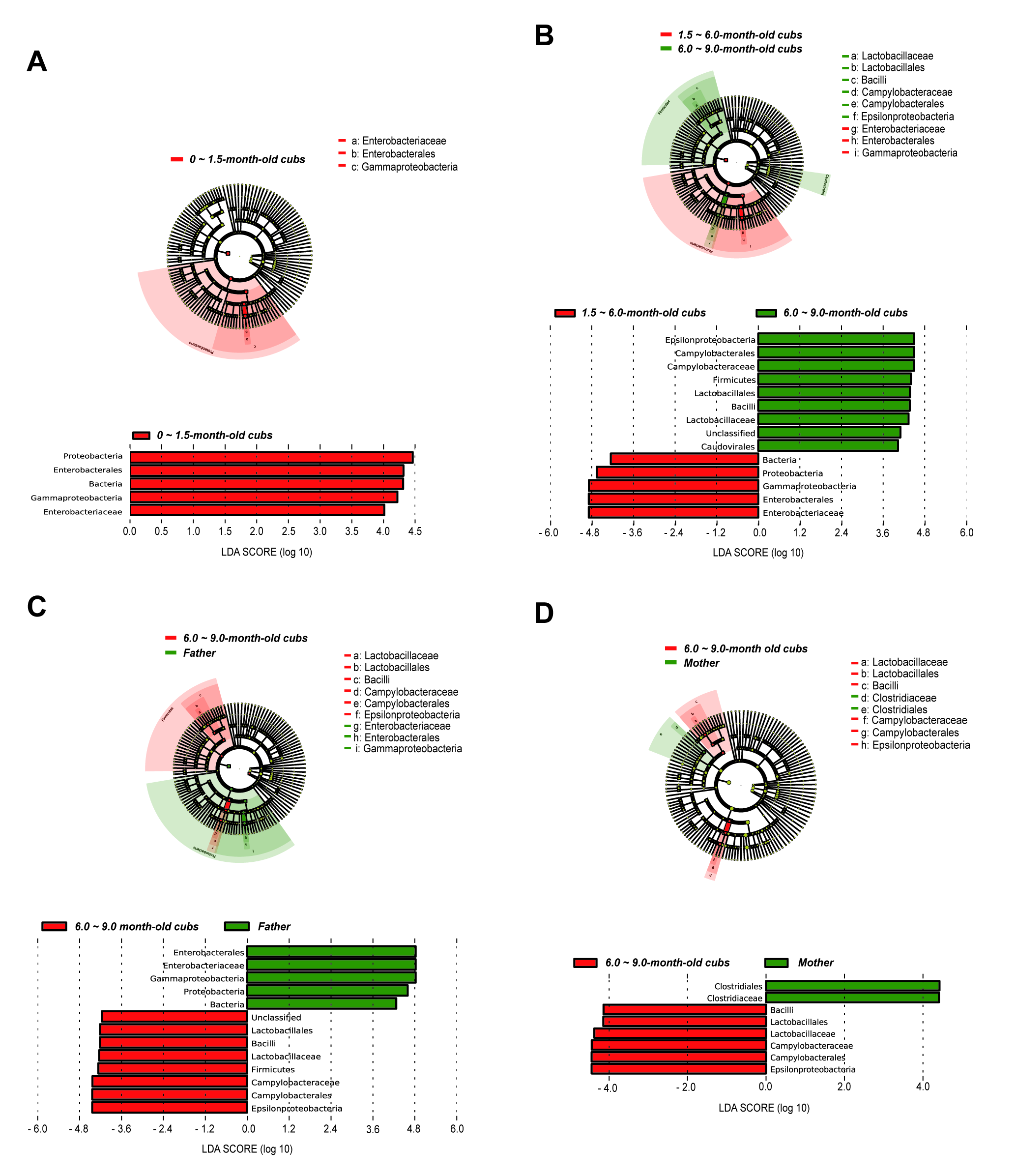

Supplement: FIGURE S3 — Gut microbial biomarkers during the development of giant panda cubs and parents. Microbial taxa with an LDA score higher than 4.0 were thought to be the representative communities of significantly changed (in abundance) microbial communities between the two microbiota groups. Circular phylogenetic trees show the relationships among biomarker taxa. (A) 0 ~ 1.5-month-old cubs versus 1.5 ~ 6-month-old cubs. (B) 1.5 ~ 6-month-old cubs versus 6 ~ 9-month-old cubs. (C) 6 ~ 9-month-old cubs versus father. (D) 6 ~ 9-month-old cubs versus mother. [file Image_3.TIF]

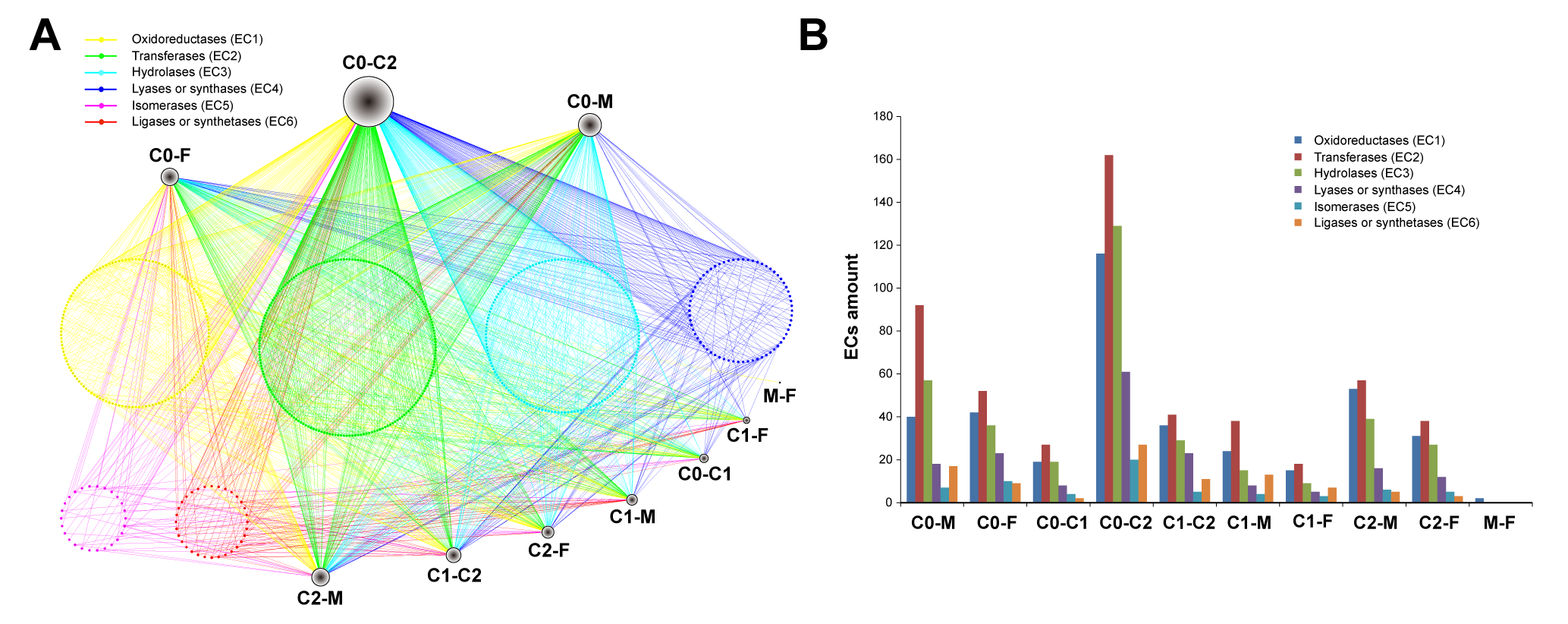

Supplement: FIGURE S4 — Enzymatic transitions in gut microbiota of giant pandas with age. (A) Significant (Wilcoxon test, adjusted P < 0.05 by Holm method) gut microbial ECs between age groups of giant pandas. All 603 ECs were clustered by the six enzymatic classifications and colored accordingly. The circle size of the node indicates the sum of significant ECs between the labeled two age groups. (B) Significantly different gut microbial EC counts between age groups of giant pandas. [file Image_4.TIF]
