# Supplementary material for: Dynamics of Gut Microbiome in Giant Panda Cubs Reveal Transitional Microbes and Pathways in Early Life
Source: Front Microbiol. 2018 Dec 18;9:3138. doi: 10.3389/fmicb.2018.03138 (PMC6305432; doi:10.3389/fmicb.2018.03138)
Supplement: TABLE S1 — Filtration of reads with low sequencing quality, reads polluted by adapter sequences and reads from giant panda or bamboo genome. [file Table_1.DOCX]

**Table S1 Filtrations of reads with low sequencing quality, reads polluted by adapter sequences and reads from giant panda or bamboo genome**

| **Sample** | **Raw data** | **Clean data** | **Clean data rate(%)** | **Panda genomic reads** | **bamboo genomic reads** | **Left** | **Left rate(%)** |
| --- | --- | --- | --- | --- | --- | --- | --- |
| P1-170315 | 144784264 | 144506757 | 99.8083 | 9979 | 819938 | 143676840 | 99.2351 |
| P1-170416 | 175089005 | 174374093 | 99.5917 | 13292 | 967632 | 173393169 | 99.0314 |
| P1-170515 | 168178632 | 167827120 | 99.791 | 3168 | 320091 | 167503861 | 99.5988 |
| P1-170616 | 180541421 | 180181023 | 99.8004 | 19829 | 479619 | 179681575 | 99.5237 |
| P1-170815 | 141734637 | 140549576 | 99.1639 | 8495 | 621155 | 139919926 | 98.7196 |
| P2-170215 | 132796204 | 132430193 | 99.7244 | 1384207 | 1293426 | 129752560 | 97.708 |
| P2-170315 | 133599201 | 133385130 | 99.8398 | 3375 | 509831 | 132871924 | 99.4556 |
| P2-170416 | 189469265 | 188417210 | 99.4447 | 241283 | 3657527 | 184518400 | 97.387 |
| P2-170515 | 153398050 | 152744286 | 99.5738 | 4490 | 506235 | 152233561 | 99.2409 |
| P2-170720 | 109076126 | 108354483 | 99.3384 | 143161 | 94060 | 108117262 | 99.1209 |
| P2-170815 | 174827553 | 173974177 | 99.5119 | 47387 | 851187 | 173075603 | 98.9979 |
| P3-160627 | 170758698 | 161604908 | 94.63934189 | 936693 | 436108 | 160232107 | 93.83539982 |
| P3-160704 | 127492449 | 127092414 | 99.6862 | 131608 | 321496 | 126639310 | 99.3308 |
| P3-160713 | 169043569 | 94999444 | 56.1982006 | 175182 | 46870 | 94777392 | 56.06684274 |
| P3-160809 | 161092138 | 85795302 | 53.25852836 | 12113417 | 60275 | 73621610 | 45.70155373 |
| P3-160815 | 114946209 | 114300028 | 99.4378 | 88535606 | 81164 | 25683258 | 22.3437 |
| P3-160821 | 236693057 | 231983324 | 98.0102 | 3422551 | 963559 | 227597214 | 96.1571 |
| P3-161011 | 147069904 | 86969060 | 59.13450518 | 4333281 | 197771 | 82438008 | 56.05362196 |
| P3-161213 | 186483193 | 153237559 | 82.17231619 | 45290566 | 99483 | 107847510 | 57.83229484 |
| P3-170118 | 133315156 | 132886320 | 99.6783 | 3470779 | 575545 | 128839996 | 96.6432 |
| P3-170219 | 121888612 | 121533712 | 99.7088 | 2969096 | 396801 | 118167815 | 96.9474 |
| P3-170319 | 174489782 | 173331704 | 99.3363 | 3817568 | 742541 | 168771595 | 96.7229 |
| P4-160627 | 114932138 | 55915325 | 48.65073075 | 199686 | 3185 | 55712454 | 48.47421702 |
| P4-160704 | 136092382 | 135515618 | 99.5762 | 1025973 | 513890 | 133975755 | 98.4447 |
| P4-160713 | 139127600 | 137733178 | 98.9977 | 414091 | 579291 | 136739796 | 98.2837 |
| P4-160809 | 175872485 | 175134407 | 99.5803 | 91338131 | 110222 | 83686054 | 47.5834 |
| P4-160815 | 92086519 | 91623075 | 99.4967 | 5633846 | 332178 | 85657051 | 93.018 |
| P4-160821 | 171085514 | 169286958 | 98.9487 | 20241355 | 818705 | 148226898 | 86.6391 |
| P4-160827 | 162810496 | 119013227 | 73.09923495 | 93483 | 338690 | 118581054 | 72.83378954 |
| P4-161214 | 172880031 | 98160567 | 56.77958665 | 6130593 | 109195 | 91920779 | 53.17026985 |
| P4-170118 | 157506304 | 156625335 | 99.4407 | 10707892 | 744629 | 145172814 | 92.1695 |
| P4-170214 | 155784952 | 155046288 | 99.5258 | 1830813 | 672821 | 152542654 | 97.9187 |
| P4-170319 | 179131022 | 177674645 | 99.187 | 7473949 | 785659 | 169415037 | 94.576 |
