# Supplementary material for: Dynamics of Gut Microbiome in Giant Panda Cubs Reveal Transitional Microbes and Pathways in Early Life
Source: Front Microbiol. 2018 Dec 18;9:3138. doi: 10.3389/fmicb.2018.03138 (PMC6305432; doi:10.3389/fmicb.2018.03138)
Supplement: TABLE S2 — Assembly of high-quality microbial reads of samples. [file Table_2.DOCX]

**Table S2 Assembly of high quality microbial reads of samples**

| **Sample** | **Total length (bp)** | **N50 (bp)** | **N90 (bp)** |
| --- | --- | --- | --- |
| P1-170315 | 129405606 | 1480 | 327 |
| P1-170416 | 175330809 | 1387 | 331 |
| P1-170515 | 364942569 | 1037 | 295 |
| P1-170616 | 161280376 | 1275 | 312 |
| P1-170815 | 118185071 | 918 | 302 |
| P2-170215 | 79916712 | 1503 | 345 |
| P2-170315 | 111761843 | 1023 | 295 |
| P2-170416 | 101044673 | 1114 | 323 |
| P2-170515 | 290508502 | 1233 | 299 |
| P2-170720 | 234991098 | 1872 | 369 |
| P2-170815 | 210929332 | 1351 | 320 |
| P3-160627 | 13900710 | 1680 | 315 |
| P3-160704 | 28321274 | 1291 | 341 |
| P3-160713 | 10978906 | 3171 | 579 |
| P3-160809 | 29825137 | 4871 | 574 |
| P3-160815 | 34712692 | 3100 | 417 |
| P3-160821 | 59324845 | 1719 | 371 |
| P3-161011 | 34233915 | 2990 | 433 |
| P3-161213 | 61866879 | 5817 | 376 |
| P3-170118 | 69171479 | 2284 | 357 |
| P3-170219 | 59464847 | 6325 | 413 |
| P3-170319 | 101004219 | 1451 | 332 |
| P4-160627 | 6450400 | 1109 | 370 |
| P4-160704 | 17251656 | 20430 | 741 |
| P4-160713 | 29287963 | 24219 | 2224 |
| P4-160809 | 41440959 | 2609 | 417 |
| P4-160815 | 42570773 | 2987 | 415 |
| P4-160821 | 47550797 | 2327 | 383 |
| P4-160827 | 64309498 | 1614 | 291 |
| P4-161214 | 48667471 | 2310 | 303 |
| P4-170118 | 105083160 | 3781 | 334 |
| P4-170214 | 84828751 | 2011 | 341 |
| P4-170319 | 110797263 | 2091 | 332 |
