# Supplementary material for: Dynamics of Gut Microbiome in Giant Panda Cubs Reveal Transitional Microbes and Pathways in Early Life
Source: Front Microbiol. 2018 Dec 18;9:3138. doi: 10.3389/fmicb.2018.03138 (PMC6305432; doi:10.3389/fmicb.2018.03138)
Supplement: TABLE S3 — Statistics of genes in non-redundant gene sets and samples. [file Table_3.docx]

**Table S3 Statistics of genes in non-redundant gene sets and samples**

| **Sample** | **Total Number** | **Total length (bp)** | **Average Length (bp)** | **N50 Length (bp)** | **GC** | **reads mapped non-redundant gene set** |
| --- | --- | --- | --- | --- | --- | --- |
| P1-170315 | 241021 | 103702830 | 430.26 | 666 | 51.69% | 55.42% |
| P1-170416 | 330284 | 140139186 | 424.3 | 645 | 49.86% | 58.83% |
| P1-170515 | 752748 | 292813956 | 388.99 | 570 | 52.97% | 49.69% |
| P1-170616 | 305976 | 123898035 | 404.93 | 618 | 45.06% | 49.93% |
| P1-170815 | 251685 | 93496974 | 371.48 | 525 | 51.74% | 60.01% |
| P2-170215 | 138841 | 59361768 | 427.55 | 669 | 42.94% | 54.22% |
| P2-170315 | 231325 | 88521819 | 382.67 | 549 | 52.22% | 53.56% |
| P2-170416 | 165862 | 66714726 | 402.23 | 645 | 40.37% | 58.19% |
| P2-170515 | 573455 | 231393438 | 403.51 | 615 | 50.59% | 49.47% |
| P2-170720 | 403039 | 190353516 | 472.3 | 729 | 53.01% | 46.95% |
| P2-170815 | 401890 | 168429279 | 419.09 | 636 | 50.53% | 56.95% |
| P3-160627 | 26433 | 10870851 | 411.26 | 624 | 53.41% | 79.97% |
| P3-160704 | 63287 | 24166809 | 381.86 | 621 | 55.62% | 56.80% |
| P3-160713 | 16519 | 9134334 | 552.96 | 855 | 54.95% | 65.56% |
| P3-160809 | 40848 | 24467223 | 598.98 | 909 | 53.21% | 70.78% |
| P3-160815 | 50801 | 26332449 | 518.35 | 903 | 54.38% | 59.49% |
| P3-160821 | 124993 | 50612607 | 404.92 | 687 | 51.60% | 67.99% |
| P3-161011 | 54048 | 27238815 | 503.97 | 798 | 47.98% | 67.80% |
| P3-161213 | 93472 | 49017402 | 524.41 | 861 | 46.41% | 73.83% |
| P3-170118 | 153414 | 59214798 | 385.98 | 696 | 45.76% | 62.14% |
| P3-170219 | 100292 | 49814319 | 496.69 | 852 | 45.57% | 59.37% |
| P3-170319 | 324644 | 93760284 | 288.81 | 522 | 48.12% | 62.87% |
| P4-160627 | 13095 | 5247573 | 400.73 | 579 | 56.62% | 57.53% |
| P4-160704 | 22106 | 14962665 | 676.86 | 1026 | 55.38% | 58.60% |
| P4-160713 | 32649 | 25556148 | 782.75 | 1089 | 52.94% | 58.86% |
| P4-160809 | 61787 | 30955359 | 501 | 867 | 52.18% | 57.44% |
| P4-160815 | 77367 | 36661203 | 473.86 | 819 | 51.66% | 62.23% |
| P4-160821 | 87883 | 39276198 | 446.91 | 750 | 50.21% | 64.85% |
| P4-160827 | 123963 | 51089166 | 412.13 | 657 | 48.39% | 66.24% |
| P4-161214 | 88739 | 38387388 | 432.59 | 708 | 48.29% | 70.02% |
| P4-170118 | 387006 | 107462424 | 277.68 | 576 | 48.82% | 63.41% |
| P4-170214 | 228175 | 76115310 | 333.58 | 633 | 43.33% | 62.89% |
| P4-170319 | 385193 | 108749706 | 282.33 | 549 | 45.51% | 64.63% |
| **cdhit (non-redundant gene set)** | **1053600** | **569646306** | **540.67** | **819** | **49.75%** | - |
