# Supplementary material for: Dynamics of Gut Microbiome in Giant Panda Cubs Reveal Transitional Microbes and Pathways in Early Life
Source: Front Microbiol. 2018 Dec 18;9:3138. doi: 10.3389/fmicb.2018.03138 (PMC6305432; doi:10.3389/fmicb.2018.03138)
Supplement: TABLE S4 — Abundance of gut microbial genera at the birth (~1 day) of giant panda cubs. [file Table_4.DOCX]

**Table S4**  **Abundance of gut microbial genera at the birth ( ~ 1 day) of giant panda cubs**

| **Genus** | **P3-160627** | **P4-160627** |
| --- | --- | --- |
| *Jerseyvirus* | 3.43469E-06 | 3.76834E-08 |
| *Vi1virus* | 5.78723E-07 | 0 |
| *Phieco32virus* | 0 | 1.61276E-08 |
| *Phikmvvirus* | 0.000117648 | 3.61168E-06 |
| *N4virus* | 0 | 5.67758E-09 |
| *T5virus* | 2.04818E-06 | 2.71416E-08 |
| *P22virus* | 4.54071E-06 | 0 |
| *P1virus* | 0.000396567 | 6.57825E-08 |
| *Muvirus* | 5.05717E-05 | 0 |
| *Lambdavirus* | 0.000230995 | 0 |
| *P2virus* | 4.62315E-05 | 0 |
| *T4virus* | 2.31202E-05 | 3.59492E-07 |
| *Methanosphaera* | 0 | 1.42631E-07 |
| *Methanobacterium* | 0 | 5.39542E-08 |
| *Brachyspira* | 0 | 1.17446E-07 |
| *Sneathia* | 0 | 4.81213E-08 |
| *Fusobacterium* | 6.53536E-08 | 4.98966E-07 |
| *Mucilaginibacter* | 1.33018E-08 | 0 |
| *Alistipes* | 0 | 1.14134E-07 |
| *Sphingobacterium* | 6.52507E-08 | 6.5363E-09 |
| *Bacteroides* | 0 | 3.27296E-08 |
| *Pseudarthrobacter* | 1.83512E-09 | 3.81424E-09 |
| *Glutamicibacter* | 1.10556E-06 | 0 |
| *Sinomonas* | 6.26418E-06 | 0 |
| *Microterricola* | 7.59818E-05 | 0 |
| *Beutenbergia* | 8.16301E-07 | 0 |
| *Kocuria* | 4.4378E-06 | 1.39756E-07 |
| *Microbacterium* | 5.35397E-07 | 2.69771E-08 |
| *Rothia* | 1.8054E-05 | 0 |
| *Streptomyces* | 0 | 1.93531E-07 |
| *Propionibacterium* | 0.00107033 | 8.46631E-06 |
| *Corynebacterium* | 9.29782E-06 | 0 |
| *Arthrobacter* | 1.19774E-06 | 1.27178E-08 |
| *Micrococcus* | 5.26769E-08 | 0 |
| *Clostridioides* | 0 | 2.52295E-06 |
| *Paeniclostridium* | 7.87084E-08 | 3.27887E-06 |
| *Lachnoclostridium* | 0 | 3.69703E-08 |
| *Gottschalkia* | 6.7558E-09 | 8.95075E-07 |
| *Cellulosilyticum* | 0 | 7.36203E-07 |
| *Blautia* | 0 | 1.75884E-07 |
| *Parvimonas* | 0 | 2.29444E-08 |
| *Pelosinus* | 0 | 1.27961E-07 |
| *Anaerostipes* | 0 | 1.71201E-07 |
| *Turicibacter* | 4.02554E-09 | 2.72814E-07 |
| *Oceanobacillus* | 0 | 3.95664E-07 |
| *Megamonas* | 0 | 4.48336E-07 |
| *Alkaliphilus* | 0 | 1.95658E-07 |
| *Candidatus_Arthromitus* | 0 | 1.52738E-07 |
| *Weissella* | 4.09783E-06 | 0 |
| *Paenibacillus* | 1.72191E-07 | 1.52635E-07 |
| *Melissococcus* | 0 | 2.19922E-07 |
| *Amphibacillus* | 0 | 4.78626E-08 |
| *Carnobacterium* | 0 | 3.64854E-08 |
| *Halanaerobium* | 0 | 1.22858E-07 |
| *Eubacterium* | 0 | 3.11107E-07 |
| *Lactobacillus* | 1.03366E-07 | 1.51531E-07 |
| *Desulfotomaculum* | 0 | 8.10951E-08 |
| *Clostridium* | 1.34367E-06 | 0.000140662 |
| *Bacillus* | 8.70565E-09 | 1.5757E-07 |
| *Lactococcus* | 8.05841E-08 | 8.60261E-08 |
| *Enterococcus* | 2.53909E-05 | 1.33752E-06 |
| *Streptococcus* | 1.836E-06 | 4.07956E-07 |
| *Staphylococcus* | 4.60282E-07 | 1.19092E-06 |
| *Leuconostoc* | 8.90269E-08 | 0 |
| *Selenomonas* | 0 | 1.20303E-07 |
| *Roseburia* | 8.914E-09 | 3.28862E-07 |
| *Chania* | 2.26019E-06 | 0 |
| *Frischella* | 0 | 8.86305E-08 |
| *Pluralibacter* | 0.000851683 | 4.73001E-06 |
| *Gilliamella* | 0 | 1.20554E-07 |
| *Pseudogulbenkiania* | 0 | 8.06381E-09 |
| *Cronobacter* | 0.00065904 | 2.41182E-05 |
| *Histophilus* | 1.07618E-08 | 0 |
| *Dickeya* | 1.65877E-05 | 1.29528E-07 |
| *Candidatus_Pelagibacter* | 3.43754E-09 | 0 |
| *Sphingobium* | 6.83486E-08 | 0 |
| *Raoultella* | 0.000189519 | 0.000439421 |
| *Yokenella* | 0.000145344 | 1.45425E-05 |
| *Cedecea* | 6.81864E-07 | 3.27296E-08 |
| *Pectobacterium* | 9.75376E-05 | 7.34975E-06 |
| *Leclercia* | 4.88853E-05 | 1.073E-05 |
| *Obesumbacterium* | 0.000110774 | 7.71753E-06 |
| *Alcanivorax* | 8.98086E-07 | 0 |
| *Pantoea* | 0.000267124 | 2.55993E-05 |
| *Rahnella* | 0.000608105 | 2.87176E-08 |
| *Photorhabdus* | 1.84933E-06 | 0 |
| *Halomonas* | 2.99822E-07 | 0 |
| *Nitrosococcus* | 1.2783E-05 | 3.53467E-08 |
| *Haemophilus* | 1.24092E-06 | 0 |
| *Vibrio* | 0.000227731 | 2.28418E-05 |
| *Aeromonas* | 0.000285055 | 4.54089E-06 |
| *Edwardsiella* | 0.000473249 | 2.06609E-05 |
| *Yersinia* | 0.002140887 | 2.61544E-05 |
| *Xenorhabdus* | 1.04715E-07 | 0 |
| *Shigella* | 0.061991464 | 0.000798895 |
| *Serratia* | 0.001002273 | 0.010980031 |
| *Salmonella* | 0.024604606 | 0.004699366 |
| *Providencia* | 4.10881E-06 | 0 |
| *Proteus* | 2.80846E-06 | 5.55306E-07 |
| *Morganella* | 1.94473E-06 | 2.35178E-05 |
| *Kluyvera* | 0.000149242 | 3.19703E-06 |
| *Klebsiella* | 0.00731354 | 0.362761611 |
| *Hafnia* | 3.47595E-05 | 1.45169E-06 |
| *Escherichia* | 0.814654727 | 0.146912022 |
| *Erwinia* | 6.09827E-07 | 0 |
| *Enterobacter* | 0.01853931 | 0.083615135 |
| *Citrobacter* | 0.001593827 | 0.000623517 |
| *Psychrobacter* | 4.6075E-06 | 0 |
| *Moraxella* | 1.48866E-07 | 0 |
| *Acinetobacter* | 6.79097E-06 | 1.02907E-07 |
| *Xanthomonas* | 1.00872E-07 | 0 |
| *Pseudomonas* | 0.000285852 | 4.38707E-05 |
| *Alteromonas* | 3.47947E-07 | 0 |
| *Campylobacter* | 5.47642E-07 | 1.27557E-06 |
| *Shewanella* | 1.48867E-07 | 0 |
