# Supplementary material for: Dynamics of Gut Microbiome in Giant Panda Cubs Reveal Transitional Microbes and Pathways in Early Life
Source: Front Microbiol. 2018 Dec 18;9:3138. doi: 10.3389/fmicb.2018.03138 (PMC6305432; doi:10.3389/fmicb.2018.03138)
Supplement: TABLE S5 — Significantly changed ECs with age (C0: 0 ~ 1.5-month-old cubs; C1: 1.5 ~ 6-month-old cubs; C2: 6 ~ 9-month-old cubs; F, father; M, mother). [file Table_5.DOCX]

**Table S5**  **Significantly changed ECs with age (C0: 0~1.5-month-old cubs; C1: 1.5~6.0-month-old cubs; C2: 6.0~9.0-month-old cubs; F: father; M: mother)**

| **EC no.** | **Group transition** | **Adjusted p value** |
| --- | --- | --- |
| EC1.1.1.173 | C0C1 | 0.039985 |
| EC1.1.1.29 | C0C1 | 0.0399474 |
| EC1.1.1.377 | C0C1 | 0.039985 |
| EC1.1.1.378 | C0C1 | 0.039985 |
| EC1.1.1.51 | C0C1 | 0.00864225 |
| EC1.1.2.4 | C0C1 | 0.04912905 |
| EC1.11.1.1 | C0C1 | 0.04567381 |
| EC1.12.5.1 | C0C1 | 0.03498687 |
| EC1.14.13.9 | C0C1 | 0.01553232 |
| EC1.14.19.3 | C0C1 | 0.03552408 |
| EC1.20.4.3 | C0C1 | 0.04885626 |
| EC1.3.1.101 | C0C1 | 0.00422007 |
| EC1.3.1.14 | C0C1 | 0.03153061 |
| EC1.3.3.5 | C0C1 | 0.04453 |
| EC1.3.4.1 | C0C1 | 0.01009557 |
| EC1.3.7.11 | C0C1 | 0.00422007 |
| EC1.4.1.16 | C0C1 | 0.04367027 |
| EC1.7.1.6 | C0C1 | 0.04453 |
| EC1.8.5.5 | C0C1 | 0.02904221 |
| EC2.1.1.148 | C0C1 | 0.04676606 |
| EC2.1.1.217 | C0C1 | 0.04429267 |
| EC2.1.1.219 | C0C1 | 0.03869648 |
| EC2.1.1.220 | C0C1 | 0.03869648 |
| EC2.3.1.201 | C0C1 | 0.0317289 |
| EC2.3.1.203 | C0C1 | 0.0486787 |
| EC2.3.1.81 | C0C1 | 0.03552408 |
| EC2.3.2.3 | C0C1 | 0.04429267 |
| EC2.4.1.153 | C0C1 | 0.0407943 |
| EC2.4.1.250 | C0C1 | 0.04697436 |
| EC2.4.1.278 | C0C1 | 0.01298002 |
| EC2.4.1.292 | C0C1 | 0.03026517 |
| EC2.4.1.293 | C0C1 | 0.03552408 |
| EC2.4.1.56 | C0C1 | 0.01009557 |
| EC2.4.1.58 | C0C1 | 0.04892399 |
| EC2.4.2.28 | C0C1 | 0.0306729 |
| EC2.4.99.19 | C0C1 | 0.04059894 |
| EC2.5.1.63 | C0C1 | 0.0215541 |
| EC2.6.1.21 | C0C1 | 0.01974457 |
| EC2.6.1.34 | C0C1 | 0.04059894 |
| EC2.7.1.144 | C0C1 | 0.04263401 |
| EC2.7.1.204 | C0C1 | 0.03821148 |
| EC2.7.1.207 | C0C1 | 0.02124849 |
| EC2.7.1.66 | C0C1 | 0.03619648 |
| EC2.7.10.1 | C0C1 | 0.02218168 |
| EC2.7.6.2 | C0C1 | 0.04429267 |
| EC2.7.7.40 | C0C1 | 0.04886889 |
| EC3.1.26.8 | C0C1 | 0.02220116 |
| EC3.1.3.41 | C0C1 | 0.03153061 |
| EC3.1.6.14 | C0C1 | 0.01477748 |
| EC3.1.6.6 | C0C1 | 0.01796365 |
| EC3.1.6.8 | C0C1 | 0.010008 |
| EC3.2.1.11 | C0C1 | 0.01230656 |
| EC3.2.1.46 | C0C1 | 0.02729392 |
| EC3.2.1.85 | C0C1 | 0.01971705 |
| EC3.4.14.11 | C0C1 | 0.04263401 |
| EC3.4.14.13 | C0C1 | 0.03619648 |
| EC3.4.14.5 | C0C1 | 0.02729392 |
| EC3.4.21.72 | C0C1 | 0.03174107 |
| EC3.4.21.96 | C0C1 | 0.02570673 |
| EC3.4.24.40 | C0C1 | 0.0091174 |
| EC3.5.1.119 | C0C1 | 0.02222744 |
| EC3.6.3.4 | C0C1 | 0.03815969 |
| EC3.6.3.6 | C0C1 | 0.03845878 |
| EC3.6.3.9 | C0C1 | 0.01371065 |
| EC3.6.4.6 | C0C1 | 0.04498312 |
| EC4.1.1.4 | C0C1 | 0.04433025 |
| EC4.1.1.70 | C0C1 | 0.0281995 |
| EC4.1.1.96 | C0C1 | 0.03498687 |
| EC4.1.2.43 | C0C1 | 0.02656062 |
| EC4.2.1.53 | C0C1 | 0.03217465 |
| EC4.2.2.2 | C0C1 | 0.00526602 |
| EC4.2.2.9 | C0C1 | 0.0023623 |
| EC4.2.99.21 | C0C1 | 0.01353078 |
| EC5.1.1.21 | C0C1 | 0.03845878 |
| EC5.1.2.2 | C0C1 | 0.04367027 |
| EC5.3.1.26 | C0C1 | 0.04429267 |
| EC5.3.1.27 | C0C1 | 0.04567381 |
| EC6.3.1.12 | C0C1 | 0.0399474 |
| EC6.4.1.3 | C0C1 | 0.02425559 |
| EC1.1.1.136 | C0C2 | 0.01375591 |
| EC1.1.1.137 | C0C2 | 0.01503421 |
| EC1.1.1.140 | C0C2 | 0.02856304 |
| EC1.1.1.159 | C0C2 | 0.01375591 |
| EC1.1.1.173 | C0C2 | 0.0023623 |
| EC1.1.1.205 | C0C2 | 0.01375591 |
| EC1.1.1.206 | C0C2 | 0.00863613 |
| EC1.1.1.21 | C0C2 | 0.02856304 |
| EC1.1.1.220 | C0C2 | 0.0023623 |
| EC1.1.1.23 | C0C2 | 0.01375591 |
| EC1.1.1.261 | C0C2 | 0.00837129 |
| EC1.1.1.264 | C0C2 | 0.02139101 |
| EC1.1.1.27 | C0C2 | 0.01375591 |
| EC1.1.1.271 | C0C2 | 0.01375591 |
| EC1.1.1.272 | C0C2 | 0.01255673 |
| EC1.1.1.287 | C0C2 | 0.00165361 |
| EC1.1.1.29 | C0C2 | 0.01162834 |
| EC1.1.1.302 | C0C2 | 0.04512558 |
| EC1.1.1.316 | C0C2 | 0.03027481 |
| EC1.1.1.325 | C0C2 | 0.00165361 |
| EC1.1.1.337 | C0C2 | 0.01018407 |
| EC1.1.1.363 | C0C2 | 0.01375591 |
| EC1.1.1.367 | C0C2 | 0.01843984 |
| EC1.1.1.370 | C0C2 | 0.00212607 |
| EC1.1.1.377 | C0C2 | 0.0023623 |
| EC1.1.1.378 | C0C2 | 0.0023623 |
| EC1.1.1.385 | C0C2 | 0.02464631 |
| EC1.1.1.39 | C0C2 | 0.00165361 |
| EC1.1.1.398 | C0C2 | 0.04912905 |
| EC1.1.1.403 | C0C2 | 0.03527886 |
| EC1.1.1.42 | C0C2 | 0.01375591 |
| EC1.1.1.49 | C0C2 | 0.01375591 |
| EC1.1.1.51 | C0C2 | 0.00631345 |
| EC1.1.1.85 | C0C2 | 0.04048875 |
| EC1.1.1.88 | C0C2 | 0.0126628 |
| EC1.1.1.9 | C0C2 | 0.00165361 |
| EC1.1.2.4 | C0C2 | 0.01233733 |
| EC1.1.2.6 | C0C2 | 0.00863613 |
| EC1.1.3.21 | C0C2 | 0.01331156 |
| EC1.1.3.46 | C0C2 | 0.00165361 |
| EC1.10.2.2 | C0C2 | 0.0023623 |
| EC1.11.1.1 | C0C2 | 0.0126628 |
| EC1.11.1.5 | C0C2 | 0.01375591 |
| EC1.12.5.1 | C0C2 | 0.0023623 |
| EC1.12.98.1 | C0C2 | 0.04982245 |
| EC1.12.99.6 | C0C2 | 0.04048875 |
| EC1.13.11.49 | C0C2 | 0.01233733 |
| EC1.13.11.53 | C0C2 | 0.02856304 |
| EC1.13.11.54 | C0C2 | 0.02856304 |
| EC1.13.12.- | C0C2 | 0.0126628 |
| EC1.14.13.8 | C0C2 | 0.0023623 |
| EC1.14.13.9 | C0C2 | 0.01018407 |
| EC1.14.18.2 | C0C2 | 0.03821148 |
| EC1.14.19.1 | C0C2 | 0.02183513 |
| EC1.14.19.3 | C0C2 | 0.0023623 |
| EC1.15.1.2 | C0C2 | 0.0023623 |
| EC1.16.1.1 | C0C2 | 0.01375591 |
| EC1.17.1.8 | C0C2 | 0.01375591 |
| EC1.17.4.1 | C0C2 | 0.01375591 |
| EC1.18.1.1 | C0C2 | 0.02183513 |
| EC1.2.1.21 | C0C2 | 0.01375591 |
| EC1.2.1.22 | C0C2 | 0.01375591 |
| EC1.2.1.41 | C0C2 | 0.03643988 |
| EC1.2.1.58 | C0C2 | 0.00165361 |
| EC1.2.1.76 | C0C2 | 0.00165361 |
| EC1.2.1.9 | C0C2 | 0.00631345 |
| EC1.2.3.3 | C0C2 | 0.0126628 |
| EC1.2.4.4 | C0C2 | 0.02656062 |
| EC1.2.7.- | C0C2 | 0.01375591 |
| EC1.2.7.1 | C0C2 | 0.01375591 |
| EC1.2.7.11 | C0C2 | 0.0126628 |
| EC1.2.7.3 | C0C2 | 0.0126628 |
| EC1.2.7.5 | C0C2 | 0.01375591 |
| EC1.2.7.8 | C0C2 | 0.0023623 |
| EC1.20.4.3 | C0C2 | 0.02500547 |
| EC1.21.4.1 | C0C2 | 0.0023623 |
| EC1.21.4.2 | C0C2 | 0.01503421 |
| EC1.21.4.3 | C0C2 | 0.00592428 |
| EC1.21.4.4 | C0C2 | 0.00592428 |
| EC1.21.98.1 | C0C2 | 0.0023623 |
| EC1.21.98.3 | C0C2 | 0.03404832 |
| EC1.3.1.1 | C0C2 | 0.01375591 |
| EC1.3.1.10 | C0C2 | 0.01375591 |
| EC1.3.1.101 | C0C2 | 0.00422007 |
| EC1.3.1.14 | C0C2 | 0.01331156 |
| EC1.3.1.33 | C0C2 | 0.01517865 |
| EC1.3.1.44 | C0C2 | 0.0023623 |
| EC1.3.1.74 | C0C2 | 0.01230656 |
| EC1.3.3.5 | C0C2 | 0.01018407 |
| EC1.3.4.1 | C0C2 | 0.00631345 |
| EC1.3.5.4 | C0C2 | 0.02856304 |
| EC1.3.7.11 | C0C2 | 0.00422007 |
| EC1.3.98.1 | C0C2 | 0.01331156 |
| EC1.3.99.24 | C0C2 | 0.00422007 |
| EC1.3.99.32 | C0C2 | 0.0023623 |
| EC1.3.99.33 | C0C2 | 0.02193841 |
| EC1.4.1.1 | C0C2 | 0.01932652 |
| EC1.4.1.16 | C0C2 | 0.00212607 |
| EC1.4.7.1 | C0C2 | 0.02193841 |
| EC1.5.1.39 | C0C2 | 0.03941326 |
| EC1.5.1.5 | C0C2 | 0.01375591 |
| EC1.5.1.7 | C0C2 | 0.00837129 |
| EC1.5.3.22 | C0C2 | 0.00165361 |
| EC1.5.98.2 | C0C2 | 0.00165361 |
| EC1.5.99.6 | C0C2 | 0.0023623 |
| EC1.6.3.4 | C0C2 | 0.01331156 |
| EC1.7.1.6 | C0C2 | 0.01018407 |
| EC1.7.2.2 | C0C2 | 0.01375591 |
| EC1.7.7.1 | C0C2 | 0.03164558 |
| EC1.8.1.14 | C0C2 | 0.0126628 |
| EC1.8.1.7 | C0C2 | 0.03643988 |
| EC1.8.1.9 | C0C2 | 0.01375591 |
| EC1.8.5.5 | C0C2 | 0.00422007 |
| EC1.8.98.1 | C0C2 | 0.0111036 |
| EC1.8.99.2 | C0C2 | 0.00165361 |
| EC1.9.3.1 | C0C2 | 0.01018407 |
| EC2.1.1.113 | C0C2 | 0.03164558 |
| EC2.1.1.148 | C0C2 | 0.00837129 |
| EC2.1.1.156 | C0C2 | 0.0126628 |
| EC2.1.1.157 | C0C2 | 0.01018407 |
| EC2.1.1.185 | C0C2 | 0.04048875 |
| EC2.1.1.213 | C0C2 | 0.04993425 |
| EC2.1.1.217 | C0C2 | 0.01331156 |
| EC2.1.1.219 | C0C2 | 0.0104655 |
| EC2.1.1.220 | C0C2 | 0.0104655 |
| EC2.1.1.226 | C0C2 | 0.01331156 |
| EC2.1.1.227 | C0C2 | 0.01331156 |
| EC2.1.1.234 | C0C2 | 0.04433025 |
| EC2.1.1.328 | C0C2 | 0.00165361 |
| EC2.1.1.74 | C0C2 | 0.0126628 |
| EC2.1.2.9 | C0C2 | 0.01375591 |
| EC2.1.3.2 | C0C2 | 0.03643988 |
| EC2.10.1.1 | C0C2 | 0.01993097 |
| EC2.3.1.157 | C0C2 | 0.04048875 |
| EC2.3.1.168 | C0C2 | 0.01503421 |
| EC2.3.1.180 | C0C2 | 0.01375591 |
| EC2.3.1.19 | C0C2 | 0.01844177 |
| EC2.3.1.201 | C0C2 | 0.0115378 |
| EC2.3.1.202 | C0C2 | 0.00837129 |
| EC2.3.1.203 | C0C2 | 0.00422007 |
| EC2.3.1.207 | C0C2 | 0.03821148 |
| EC2.3.1.227 | C0C2 | 0.04367027 |
| EC2.3.1.35 | C0C2 | 0.01375591 |
| EC2.3.1.48 | C0C2 | 0.03527886 |
| EC2.3.1.81 | C0C2 | 0.0023623 |
| EC2.3.1.89 | C0C2 | 0.01018407 |
| EC2.3.2.- | C0C2 | 0.02856304 |
| EC2.3.2.10 | C0C2 | 0.0126628 |
| EC2.3.2.3 | C0C2 | 0.01331156 |
| EC2.3.2.8 | C0C2 | 0.0023623 |
| EC2.3.3.10 | C0C2 | 0.0126628 |
| EC2.4.-.- | C0C2 | 0.01375591 |
| EC2.4.1.153 | C0C2 | 0.0096025 |
| EC2.4.1.161 | C0C2 | 0.00165361 |
| EC2.4.1.166 | C0C2 | 0.02656062 |
| EC2.4.1.208 | C0C2 | 0.03404832 |
| EC2.4.1.211 | C0C2 | 0.03527886 |
| EC2.4.1.212 | C0C2 | 0.0023623 |
| EC2.4.1.230 | C0C2 | 0.00094492 |
| EC2.4.1.250 | C0C2 | 0.02403561 |
| EC2.4.1.252 | C0C2 | 0.03733999 |
| EC2.4.1.278 | C0C2 | 0.00631345 |
| EC2.4.1.287 | C0C2 | 0.01018407 |
| EC2.4.1.292 | C0C2 | 0.0126628 |
| EC2.4.1.293 | C0C2 | 0.0023623 |
| EC2.4.1.336 | C0C2 | 0.0126628 |
| EC2.4.1.337 | C0C2 | 0.04048875 |
| EC2.4.1.341 | C0C2 | 0.0023623 |
| EC2.4.1.4 | C0C2 | 0.00916566 |
| EC2.4.1.44 | C0C2 | 0.00631345 |
| EC2.4.1.5 | C0C2 | 0.01331156 |
| EC2.4.1.52 | C0C2 | 0.01631992 |
| EC2.4.1.56 | C0C2 | 0.00631345 |
| EC2.4.1.57 | C0C2 | 0.01162834 |
| EC2.4.1.58 | C0C2 | 0.01162834 |
| EC2.4.1.64 | C0C2 | 0.03527886 |
| EC2.4.1.7 | C0C2 | 0.02856304 |
| EC2.4.1.8 | C0C2 | 0.01932652 |
| EC2.4.1.9 | C0C2 | 0.0126628 |
| EC2.4.2.- | C0C2 | 0.01375591 |
| EC2.4.2.17 | C0C2 | 0.01375591 |
| EC2.4.2.2 | C0C2 | 0.01331156 |
| EC2.4.2.28 | C0C2 | 0.00422007 |
| EC2.4.2.45 | C0C2 | 0.00165361 |
| EC2.4.2.6 | C0C2 | 0.01162834 |
| EC2.4.2.7 | C0C2 | 0.01375591 |
| EC2.4.99.19 | C0C2 | 0.0023623 |
| EC2.5.1.101 | C0C2 | 0.00631345 |
| EC2.5.1.120 | C0C2 | 0.0023623 |
| EC2.5.1.26 | C0C2 | 0.01375591 |
| EC2.5.1.29 | C0C2 | 0.0126628 |
| EC2.5.1.3 | C0C2 | 0.01375591 |
| EC2.5.1.30 | C0C2 | 0.0126628 |
| EC2.5.1.32 | C0C2 | 0.04993425 |
| EC2.5.1.49 | C0C2 | 0.01364393 |
| EC2.5.1.55 | C0C2 | 0.03643988 |
| EC2.5.1.63 | C0C2 | 0.01375591 |
| EC2.5.1.97 | C0C2 | 0.0023623 |
| EC2.5.1.99 | C0C2 | 0.04993425 |
| EC2.6.1.102 | C0C2 | 0.00631345 |
| EC2.6.1.106 | C0C2 | 0.01251 |
| EC2.6.1.108 | C0C2 | 0.00165361 |
| EC2.6.1.18 | C0C2 | 0.02183513 |
| EC2.6.1.21 | C0C2 | 0.01018407 |
| EC2.6.1.33 | C0C2 | 0.01257534 |
| EC2.6.1.34 | C0C2 | 0.0023623 |
| EC2.6.1.52 | C0C2 | 0.01375591 |
| EC2.6.1.83 | C0C2 | 0.00212607 |
| EC2.6.1.89 | C0C2 | 0.01251 |
| EC2.6.1.9 | C0C2 | 0.01375591 |
| EC2.6.1.92 | C0C2 | 0.00422007 |
| EC2.6.1.98 | C0C2 | 0.00837129 |
| EC2.7.1.12 | C0C2 | 0.01375591 |
| EC2.7.1.144 | C0C2 | 0.0126628 |
| EC2.7.1.162 | C0C2 | 0.01018407 |
| EC2.7.1.163 | C0C2 | 0.00631345 |
| EC2.7.1.164 | C0C2 | 0.02183513 |
| EC2.7.1.168 | C0C2 | 0.0023623 |
| EC2.7.1.204 | C0C2 | 0.0023623 |
| EC2.7.1.206 | C0C2 | 0.03941326 |
| EC2.7.1.207 | C0C2 | 0.0126628 |
| EC2.7.1.23 | C0C2 | 0.01375591 |
| EC2.7.1.36 | C0C2 | 0.0126628 |
| EC2.7.1.63 | C0C2 | 0.01847185 |
| EC2.7.1.66 | C0C2 | 0.01364393 |
| EC2.7.1.76 | C0C2 | 0.0126628 |
| EC2.7.1.8 | C0C2 | 0.01702519 |
| EC2.7.1.83 | C0C2 | 0.01375591 |
| EC2.7.1.87 | C0C2 | 0.00631345 |
| EC2.7.1.90 | C0C2 | 0.04494083 |
| EC2.7.10.1 | C0C2 | 0.01162834 |
| EC2.7.10.2 | C0C2 | 0.0126628 |
| EC2.7.11.- | C0C2 | 0.01331156 |
| EC2.7.2.1 | C0C2 | 0.01375591 |
| EC2.7.2.11 | C0C2 | 0.01375591 |
| EC2.7.2.7 | C0C2 | 0.02775145 |
| EC2.7.2.8 | C0C2 | 0.01993097 |
| EC2.7.4.- | C0C2 | 0.01331156 |
| EC2.7.4.2 | C0C2 | 0.0126628 |
| EC2.7.4.22 | C0C2 | 0.01375591 |
| EC2.7.4.6 | C0C2 | 0.01375591 |
| EC2.7.6.1 | C0C2 | 0.01375591 |
| EC2.7.6.2 | C0C2 | 0.01331156 |
| EC2.7.7.- | C0C2 | 0.01375591 |
| EC2.7.7.18 | C0C2 | 0.01375591 |
| EC2.7.7.23 | C0C2 | 0.04048875 |
| EC2.7.7.3 | C0C2 | 0.01375591 |
| EC2.7.7.33 | C0C2 | 0.00493493 |
| EC2.7.7.39 | C0C2 | 0.01162834 |
| EC2.7.7.40 | C0C2 | 0.03158791 |
| EC2.7.7.49 | C0C2 | 0.02570673 |
| EC2.7.7.53 | C0C2 | 0.0126628 |
| EC2.7.7.71 | C0C2 | 0.0023623 |
| EC2.7.7.74 | C0C2 | 0.045327 |
| EC2.7.7.81 | C0C2 | 0.0023623 |
| EC2.7.7.82 | C0C2 | 0.00422007 |
| EC2.7.7.85 | C0C2 | 0.01375591 |
| EC2.7.7.87 | C0C2 | 0.01375591 |
| EC2.7.7.9 | C0C2 | 0.01375591 |
| EC2.7.8.12 | C0C2 | 0.02856304 |
| EC2.7.8.20 | C0C2 | 0.04048875 |
| EC2.7.8.34 | C0C2 | 0.045327 |
| EC2.7.8.36 | C0C2 | 0.00837129 |
| EC2.7.8.40 | C0C2 | 0.01702519 |
| EC2.7.8.41 | C0C2 | 0.00379807 |
| EC2.7.8.44 | C0C2 | 0.02193841 |
| EC2.7.9.1 | C0C2 | 0.02497631 |
| EC2.7.9.3 | C0C2 | 0.01375591 |
| EC2.8.1.13 | C0C2 | 0.01375591 |
| EC2.8.1.6 | C0C2 | 0.04048875 |
| EC2.8.2.22 | C0C2 | 0.02835881 |
| EC2.8.3.1 | C0C2 | 0.02835881 |
| EC2.8.3.16 | C0C2 | 0.01375591 |
| EC2.8.3.19 | C0C2 | 0.02856304 |
| EC2.8.3.8 | C0C2 | 0.02856304 |
| EC2.8.3.9 | C0C2 | 0.01331156 |
| EC2.8.4.4 | C0C2 | 0.01375591 |
| EC2.8.4.5 | C0C2 | 0.01162834 |
| EC3.1.-.- | C0C2 | 0.01375591 |
| EC3.1.1.29 | C0C2 | 0.01375591 |
| EC3.1.1.41 | C0C2 | 0.01162834 |
| EC3.1.1.53 | C0C2 | 0.01702519 |
| EC3.1.1.57 | C0C2 | 0.0023623 |
| EC3.1.1.83 | C0C2 | 0.01162834 |
| EC3.1.1.92 | C0C2 | 0.0023623 |
| EC3.1.12.1 | C0C2 | 0.01331156 |
| EC3.1.13.3 | C0C2 | 0.01375591 |
| EC3.1.2.20 | C0C2 | 0.0126628 |
| EC3.1.2.21 | C0C2 | 0.01331156 |
| EC3.1.21.3 | C0C2 | 0.01375591 |
| EC3.1.22.4 | C0C2 | 0.01993097 |
| EC3.1.26.3 | C0C2 | 0.04567381 |
| EC3.1.26.8 | C0C2 | 0.01331156 |
| EC3.1.3.15 | C0C2 | 0.01375591 |
| EC3.1.3.41 | C0C2 | 0.01331156 |
| EC3.1.3.7 | C0C2 | 0.04048875 |
| EC3.1.3.71 | C0C2 | 0.00212607 |
| EC3.1.3.77 | C0C2 | 0.02856304 |
| EC3.1.3.96 | C0C2 | 0.02035271 |
| EC3.1.4.37 | C0C2 | 0.00232942 |
| EC3.1.4.57 | C0C2 | 0.0023623 |
| EC3.1.6.- | C0C2 | 0.01993097 |
| EC3.1.6.14 | C0C2 | 0.02719889 |
| EC3.1.6.6 | C0C2 | 0.01162834 |
| EC3.1.6.8 | C0C2 | 0.00837129 |
| EC3.11.1.2 | C0C2 | 0.01018407 |
| EC3.2.1.10 | C0C2 | 0.01375591 |
| EC3.2.1.11 | C0C2 | 0.00379807 |
| EC3.2.1.132 | C0C2 | 0.0397828 |
| EC3.2.1.133 | C0C2 | 0.01331156 |
| EC3.2.1.135 | C0C2 | 0.01331156 |
| EC3.2.1.166 | C0C2 | 0.0023623 |
| EC3.2.1.17 | C0C2 | 0.01375591 |
| EC3.2.1.178 | C0C2 | 0.00863613 |
| EC3.2.1.184 | C0C2 | 0.00422007 |
| EC3.2.1.185 | C0C2 | 0.00165361 |
| EC3.2.1.35 | C0C2 | 0.03527886 |
| EC3.2.1.45 | C0C2 | 0.00837129 |
| EC3.2.1.46 | C0C2 | 0.00165361 |
| EC3.2.1.54 | C0C2 | 0.01331156 |
| EC3.2.1.55 | C0C2 | 0.01844177 |
| EC3.2.1.58 | C0C2 | 0.00379807 |
| EC3.2.1.65 | C0C2 | 0.03821148 |
| EC3.2.1.70 | C0C2 | 0.01364393 |
| EC3.2.1.73 | C0C2 | 0.00863613 |
| EC3.2.1.8 | C0C2 | 0.04048875 |
| EC3.2.1.82 | C0C2 | 0.0023623 |
| EC3.2.1.85 | C0C2 | 0.01162834 |
| EC3.2.1.91 | C0C2 | 0.01993097 |
| EC3.2.1.96 | C0C2 | 0.01364393 |
| EC3.2.2.1 | C0C2 | 0.01364393 |
| EC3.2.2.27 | C0C2 | 0.04048875 |
| EC3.2.2.30 | C0C2 | 0.0023623 |
| EC3.2.2.8 | C0C2 | 0.02856304 |
| EC3.4.11.19 | C0C2 | 0.00212607 |
| EC3.4.11.21 | C0C2 | 0.02390456 |
| EC3.4.11.5 | C0C2 | 0.01162834 |
| EC3.4.11.7 | C0C2 | 0.01375591 |
| EC3.4.14.11 | C0C2 | 0.0126628 |
| EC3.4.14.13 | C0C2 | 0.01364393 |
| EC3.4.14.5 | C0C2 | 0.00165361 |
| EC3.4.16.- | C0C2 | 0.01251 |
| EC3.4.17.11 | C0C2 | 0.0023623 |
| EC3.4.19.1 | C0C2 | 0.01162834 |
| EC3.4.19.11 | C0C2 | 0.01331156 |
| EC3.4.21.107 | C0C2 | 0.01375591 |
| EC3.4.21.116 | C0C2 | 0.03164558 |
| EC3.4.21.72 | C0C2 | 0.01364393 |
| EC3.4.21.88 | C0C2 | 0.02856304 |
| EC3.4.21.89 | C0C2 | 0.04048875 |
| EC3.4.21.96 | C0C2 | 0.01375591 |
| EC3.4.22.40 | C0C2 | 0.01255673 |
| EC3.4.22.70 | C0C2 | 0.01375591 |
| EC3.4.22.8 | C0C2 | 0.00863613 |
| EC3.4.23.- | C0C2 | 0.01375591 |
| EC3.4.23.36 | C0C2 | 0.01993097 |
| EC3.4.23.43 | C0C2 | 0.01375591 |
| EC3.4.24.- | C0C2 | 0.01375591 |
| EC3.4.24.3 | C0C2 | 0.00212607 |
| EC3.4.24.40 | C0C2 | 0.00658253 |
| EC3.4.24.71 | C0C2 | 0.04912905 |
| EC3.4.24.75 | C0C2 | 0.01331156 |
| EC3.4.24.84 | C0C2 | 0.0023623 |
| EC3.5.1.1 | C0C2 | 0.01993097 |
| EC3.5.1.106 | C0C2 | 0.00165361 |
| EC3.5.1.11 | C0C2 | 0.01233733 |
| EC3.5.1.119 | C0C2 | 0.00379807 |
| EC3.5.1.24 | C0C2 | 0.01364393 |
| EC3.5.1.26 | C0C2 | 0.01162834 |
| EC3.5.1.41 | C0C2 | 0.01977875 |
| EC3.5.1.44 | C0C2 | 0.00493493 |
| EC3.5.1.47 | C0C2 | 0.0126628 |
| EC3.5.1.53 | C0C2 | 0.00837129 |
| EC3.5.1.59 | C0C2 | 0.00493493 |
| EC3.5.1.77 | C0C2 | 0.034081 |
| EC3.5.1.88 | C0C2 | 0.01375591 |
| EC3.5.1.9 | C0C2 | 0.02775145 |
| EC3.5.2.9 | C0C2 | 0.00094492 |
| EC3.5.3.12 | C0C2 | 0.0126628 |
| EC3.5.3.18 | C0C2 | 0.00417012 |
| EC3.5.3.26 | C0C2 | 0.01375591 |
| EC3.5.4.16 | C0C2 | 0.01375591 |
| EC3.5.4.25 | C0C2 | 0.01375591 |
| EC3.5.4.40 | C0C2 | 0.0023623 |
| EC3.5.4.9 | C0C2 | 0.01375591 |
| EC3.5.5.7 | C0C2 | 0.0111036 |
| EC3.5.99.7 | C0C2 | 0.0023623 |
| EC3.6.1.55 | C0C2 | 0.01375591 |
| EC3.6.1.57 | C0C2 | 0.0023623 |
| EC3.6.1.66 | C0C2 | 0.01375591 |
| EC3.6.3.10 | C0C2 | 0.00863613 |
| EC3.6.3.12 | C0C2 | 0.01375591 |
| EC3.6.3.35 | C0C2 | 0.01844177 |
| EC3.6.3.36 | C0C2 | 0.02856304 |
| EC3.6.3.38 | C0C2 | 0.01162834 |
| EC3.6.3.4 | C0C2 | 0.01375591 |
| EC3.6.3.40 | C0C2 | 0.0126628 |
| EC3.6.3.44 | C0C2 | 0.01364393 |
| EC3.6.3.55 | C0C2 | 0.0023623 |
| EC3.6.3.6 | C0C2 | 0.01702519 |
| EC3.6.3.9 | C0C2 | 0.00422007 |
| EC3.6.4.12 | C0C2 | 0.01993097 |
| EC3.6.4.6 | C0C2 | 0.0023623 |
| EC3.6.5.- | C0C2 | 0.01375591 |
| EC3.6.5.4 | C0C2 | 0.01375591 |
| EC3.7.1.9 | C0C2 | 0.0497285 |
| EC3.8.1.2 | C0C2 | 0.01331156 |
| EC4.1.1.12 | C0C2 | 0.01331156 |
| EC4.1.1.19 | C0C2 | 0.04000143 |
| EC4.1.1.2 | C0C2 | 0.00863613 |
| EC4.1.1.23 | C0C2 | 0.01375591 |
| EC4.1.1.25 | C0C2 | 0.00863613 |
| EC4.1.1.33 | C0C2 | 0.0126628 |
| EC4.1.1.35 | C0C2 | 0.0091174 |
| EC4.1.1.4 | C0C2 | 0.01503421 |
| EC4.1.1.45 | C0C2 | 0.01162834 |
| EC4.1.1.48 | C0C2 | 0.01375591 |
| EC4.1.1.70 | C0C2 | 0.0096025 |
| EC4.1.1.82 | C0C2 | 0.00493493 |
| EC4.1.1.96 | C0C2 | 0.0023623 |
| EC4.1.2.22 | C0C2 | 0.0126628 |
| EC4.1.2.29 | C0C2 | 0.00165361 |
| EC4.1.2.40 | C0C2 | 0.01993097 |
| EC4.1.2.43 | C0C2 | 0.02656062 |
| EC4.1.2.50 | C0C2 | 0.01375591 |
| EC4.1.2.53 | C0C2 | 0.01993097 |
| EC4.1.2.9 | C0C2 | 0.0126628 |
| EC4.1.3.- | C0C2 | 0.01375591 |
| EC4.1.3.32 | C0C2 | 0.00493493 |
| EC4.1.3.4 | C0C2 | 0.00212607 |
| EC4.1.3.42 | C0C2 | 0.01375591 |
| EC4.1.99.1 | C0C2 | 0.04048875 |
| EC4.2.1.103 | C0C2 | 0.00863613 |
| EC4.2.1.108 | C0C2 | 0.0327527 |
| EC4.2.1.11 | C0C2 | 0.01375591 |
| EC4.2.1.115 | C0C2 | 0.0023623 |
| EC4.2.1.12 | C0C2 | 0.01375591 |
| EC4.2.1.120 | C0C2 | 0.02183513 |
| EC4.2.1.135 | C0C2 | 0.00631345 |
| EC4.2.1.149 | C0C2 | 0.01993097 |
| EC4.2.1.151 | C0C2 | 0.0023623 |
| EC4.2.1.19 | C0C2 | 0.01375591 |
| EC4.2.1.24 | C0C2 | 0.03643988 |
| EC4.2.1.33 | C0C2 | 0.01375591 |
| EC4.2.1.35 | C0C2 | 0.01375591 |
| EC4.2.1.45 | C0C2 | 0.00165361 |
| EC4.2.1.47 | C0C2 | 0.01375591 |
| EC4.2.1.53 | C0C2 | 0.01364393 |
| EC4.2.1.55 | C0C2 | 0.00837129 |
| EC4.2.1.70 | C0C2 | 0.01375591 |
| EC4.2.1.77 | C0C2 | 0.00165361 |
| EC4.2.1.90 | C0C2 | 0.04048875 |
| EC4.2.2.2 | C0C2 | 0.00422007 |
| EC4.2.2.23 | C0C2 | 0.0023623 |
| EC4.2.2.8 | C0C2 | 0.0023623 |
| EC4.2.2.9 | C0C2 | 0.0023623 |
| EC4.2.3.1 | C0C2 | 0.04048875 |
| EC4.2.3.12 | C0C2 | 0.01375591 |
| EC4.2.99.20 | C0C2 | 0.01375591 |
| EC4.2.99.21 | C0C2 | 0.01018407 |
| EC4.3.1.12 | C0C2 | 0.0126628 |
| EC4.3.1.17 | C0C2 | 0.01375591 |
| EC4.3.1.2 | C0C2 | 0.02848102 |
| EC4.3.2.2 | C0C2 | 0.01375591 |
| EC4.3.3.6 | C0C2 | 0.01844177 |
| EC4.4.1.21 | C0C2 | 0.01375591 |
| EC4.4.1.25 | C0C2 | 0.0023623 |
| EC4.6.1.12 | C0C2 | 0.01375591 |
| EC5.1.1.17 | C0C2 | 0.00165361 |
| EC5.1.1.21 | C0C2 | 0.03164558 |
| EC5.1.1.4 | C0C2 | 0.00165361 |
| EC5.1.2.2 | C0C2 | 0.0023623 |
| EC5.1.3.1 | C0C2 | 0.02856304 |
| EC5.1.3.20 | C0C2 | 0.04048875 |
| EC5.1.3.23 | C0C2 | 0.00165361 |
| EC5.1.3.24 | C0C2 | 0.01375591 |
| EC5.1.3.7 | C0C2 | 0.04912905 |
| EC5.1.99.4 | C0C2 | 0.02183513 |
| EC5.3.1.- | C0C2 | 0.01375591 |
| EC5.3.1.26 | C0C2 | 0.01331156 |
| EC5.3.1.27 | C0C2 | 0.03941326 |
| EC5.3.2.4 | C0C2 | 0.00422007 |
| EC5.3.3.3 | C0C2 | 0.02183513 |
| EC5.4.2.10 | C0C2 | 0.04048875 |
| EC5.4.2.11 | C0C2 | 0.01375591 |
| EC5.4.2.8 | C0C2 | 0.04048875 |
| EC5.4.99.1 | C0C2 | 0.0126628 |
| EC5.4.99.23 | C0C2 | 0.01375591 |
| EC6.1.1.1 | C0C2 | 0.01375591 |
| EC6.1.1.17 | C0C2 | 0.03643988 |
| EC6.1.1.20 | C0C2 | 0.04567381 |
| EC6.1.1.23 | C0C2 | 0.00212607 |
| EC6.1.1.24 | C0C2 | 0.01331156 |
| EC6.1.1.3 | C0C2 | 0.02570673 |
| EC6.1.1.4 | C0C2 | 0.03643988 |
| EC6.2.1.45 | C0C2 | 0.0111036 |
| EC6.3.1.12 | C0C2 | 0.01162834 |
| EC6.3.1.19 | C0C2 | 0.00916566 |
| EC6.3.2.13 | C0C2 | 0.04048875 |
| EC6.3.2.29 | C0C2 | 0.0165976 |
| EC6.3.2.30 | C0C2 | 0.0165976 |
| EC6.3.2.43 | C0C2 | 0.03821148 |
| EC6.3.2.6 | C0C2 | 0.01375591 |
| EC6.3.2.7 | C0C2 | 0.02390456 |
| EC6.3.3.1 | C0C2 | 0.01375591 |
| EC6.3.4.20 | C0C2 | 0.02856304 |
| EC6.3.4.21 | C0C2 | 0.01375591 |
| EC6.3.4.3 | C0C2 | 0.01331156 |
| EC6.3.5.6 | C0C2 | 0.01375591 |
| EC6.3.5.7 | C0C2 | 0.01375591 |
| EC6.4.1.1 | C0C2 | 0.01331156 |
| EC6.4.1.2 | C0C2 | 0.01993097 |
| EC6.4.1.3 | C0C2 | 0.00837129 |
| EC6.4.1.4 | C0C2 | 0.03821148 |
| EC6.5.1.4 | C0C2 | 0.01993097 |
| EC1.1.1.205 | C0F | 0.0215541 |
| EC1.1.1.23 | C0F | 0.02424836 |
| EC1.1.1.271 | C0F | 0.0215541 |
| EC1.1.1.316 | C0F | 0.03772391 |
| EC1.1.1.363 | C0F | 0.04685679 |
| EC1.1.1.367 | C0F | 0.01049686 |
| EC1.1.1.42 | C0F | 0.0215541 |
| EC1.1.1.49 | C0F | 0.04685679 |
| EC1.1.5.2 | C0F | 0.04000143 |
| EC1.11.1.5 | C0F | 0.02424836 |
| EC1.12.5.1 | C0F | 0.01741183 |
| EC1.13.11.49 | C0F | 0.0195883 |
| EC1.13.12.4 | C0F | 0.03675612 |
| EC1.14.13.8 | C0F | 0.00317881 |
| EC1.14.13.9 | C0F | 0.01553232 |
| EC1.14.19.3 | C0F | 0.00282561 |
| EC1.15.1.2 | C0F | 0.00317881 |
| EC1.16.1.1 | C0F | 0.02424836 |
| EC1.17.4.1 | C0F | 0.02424836 |
| EC1.2.1.41 | C0F | 0.02694262 |
| EC1.2.1.70 | C0F | 0.02694262 |
| EC1.2.1.88 | C0F | 0.02694262 |
| EC1.2.1.9 | C0F | 0.04524802 |
| EC1.2.7.- | C0F | 0.0215541 |
| EC1.2.7.1 | C0F | 0.0215541 |
| EC1.21.4.1 | C0F | 0.00282561 |
| EC1.21.98.1 | C0F | 0.01741183 |
| EC1.3.1.101 | C0F | 0.00497955 |
| EC1.3.1.44 | C0F | 0.00317881 |
| EC1.3.1.74 | C0F | 0.00640228 |
| EC1.3.4.1 | C0F | 0.00908306 |
| EC1.3.5.1 | C0F | 0.02694262 |
| EC1.3.7.11 | C0F | 0.00497955 |
| EC1.5.1.5 | C0F | 0.03200115 |
| EC1.5.5.2 | C0F | 0.02694262 |
| EC1.5.99.6 | C0F | 0.00317881 |
| EC1.7.99.1 | C0F | 0.02424836 |
| EC1.8.1.7 | C0F | 0.02694262 |
| EC1.8.1.9 | C0F | 0.0215541 |
| EC1.8.4.14 | C0F | 0.04000143 |
| EC1.8.5.5 | C0F | 0.00569091 |
| EC1.97.1.9 | C0F | 0.04000143 |
| EC2.1.1.148 | C0F | 0.04059894 |
| EC2.1.1.191 | C0F | 0.02424836 |
| EC2.1.2.2 | C0F | 0.02694262 |
| EC2.1.3.10 | C0F | 0.01166318 |
| EC2.3.1.180 | C0F | 0.03600129 |
| EC2.3.1.201 | C0F | 0.01021845 |
| EC2.3.1.203 | C0F | 0.00569091 |
| EC2.3.1.227 | C0F | 0.00353201 |
| EC2.3.1.81 | C0F | 0.00282561 |
| EC2.4.-.- | C0F | 0.0215541 |
| EC2.4.1.175 | C0F | 0.01617782 |
| EC2.4.1.212 | C0F | 0.00317881 |
| EC2.4.1.226 | C0F | 0.01617782 |
| EC2.4.1.250 | C0F | 0.02403561 |
| EC2.4.1.252 | C0F | 0.00711364 |
| EC2.4.1.292 | C0F | 0.03797926 |
| EC2.4.1.293 | C0F | 0.00282561 |
| EC2.4.1.341 | C0F | 0.00317881 |
| EC2.4.1.56 | C0F | 0.00908306 |
| EC2.4.1.57 | C0F | 0.01796365 |
| EC2.4.1.58 | C0F | 0.01796365 |
| EC2.4.1.7 | C0F | 0.03600129 |
| EC2.4.1.87 | C0F | 0.02799377 |
| EC2.4.2.- | C0F | 0.02424836 |
| EC2.4.2.17 | C0F | 0.04685679 |
| EC2.4.2.36 | C0F | 0.0195883 |
| EC2.5.1.54 | C0F | 0.02694262 |
| EC2.5.1.55 | C0F | 0.02694262 |
| EC2.5.1.63 | C0F | 0.0215541 |
| EC2.5.1.97 | C0F | 0.01741183 |
| EC2.6.1.102 | C0F | 0.04524802 |
| EC2.6.1.106 | C0F | 0.01405678 |
| EC2.6.1.33 | C0F | 0.01796365 |
| EC2.6.1.66 | C0F | 0.04000143 |
| EC2.6.1.89 | C0F | 0.01405678 |
| EC2.6.1.92 | C0F | 0.00569091 |
| EC2.6.1.98 | C0F | 0.01249492 |
| EC2.7.1.204 | C0F | 0.01741183 |
| EC2.7.1.23 | C0F | 0.02424836 |
| EC2.7.1.87 | C0F | 0.00908306 |
| EC2.7.1.89 | C0F | 0.02694262 |
| EC2.7.2.1 | C0F | 0.0215541 |
| EC2.7.2.11 | C0F | 0.02424836 |
| EC2.7.6.1 | C0F | 0.03200115 |
| EC2.7.7.12 | C0F | 0.02694262 |
| EC2.7.7.27 | C0F | 0.02424836 |
| EC2.7.7.49 | C0F | 0.04059894 |
| EC2.7.9.3 | C0F | 0.03200115 |
| EC2.8.1.13 | C0F | 0.0215541 |
| EC2.8.1.8 | C0F | 0.04000143 |
| EC2.8.3.9 | C0F | 0.0309682 |
| EC2.8.4.4 | C0F | 0.0215541 |
| EC3.1.1.29 | C0F | 0.04685679 |
| EC3.1.1.57 | C0F | 0.00282561 |
| EC3.1.1.88 | C0F | 0.00353201 |
| EC3.1.1.92 | C0F | 0.00282561 |
| EC3.1.13.1 | C0F | 0.02694262 |
| EC3.1.21.3 | C0F | 0.04685679 |
| EC3.1.26.3 | C0F | 0.0486787 |
| EC3.1.3.12 | C0F | 0.02694262 |
| EC3.1.3.15 | C0F | 0.03600129 |
| EC3.1.3.6 | C0F | 0.02694262 |
| EC3.1.4.16 | C0F | 0.02694262 |
| EC3.1.6.14 | C0F | 0.01405678 |
| EC3.1.6.6 | C0F | 0.01796365 |
| EC3.1.6.8 | C0F | 0.01093305 |
| EC3.2.1.133 | C0F | 0.02080374 |
| EC3.2.1.135 | C0F | 0.02080374 |
| EC3.2.1.184 | C0F | 0.04059894 |
| EC3.2.1.28 | C0F | 0.04000143 |
| EC3.2.1.54 | C0F | 0.02080374 |
| EC3.2.2.1 | C0F | 0.04650508 |
| EC3.4.17.11 | C0F | 0.00317881 |
| EC3.4.19.11 | C0F | 0.04194908 |
| EC3.4.21.62 | C0F | 0.04288692 |
| EC3.4.21.72 | C0F | 0.03174107 |
| EC3.4.24.- | C0F | 0.02424836 |
| EC3.4.24.30 | C0F | 0.0195883 |
| EC3.5.1.1 | C0F | 0.03600129 |
| EC3.5.1.28 | C0F | 0.04685679 |
| EC3.5.4.16 | C0F | 0.02424836 |
| EC3.5.4.9 | C0F | 0.03200115 |
| EC3.6.1.57 | C0F | 0.01741183 |
| EC3.6.3.33 | C0F | 0.02694262 |
| EC3.6.3.36 | C0F | 0.03600129 |
| EC3.6.3.38 | C0F | 0.01796365 |
| EC3.6.3.55 | C0F | 0.0195883 |
| EC3.6.4.6 | C0F | 0.04498312 |
| EC4.1.1.103 | C0F | 0.01135383 |
| EC4.1.1.19 | C0F | 0.04000143 |
| EC4.1.1.23 | C0F | 0.03200115 |
| EC4.1.1.48 | C0F | 0.02424836 |
| EC4.1.1.96 | C0F | 0.01741183 |
| EC4.1.3.- | C0F | 0.02424836 |
| EC4.1.3.27 | C0F | 0.02694262 |
| EC4.1.3.34 | C0F | 0.02694262 |
| EC4.1.3.36 | C0F | 0.04000143 |
| EC4.1.3.4 | C0F | 0.00247241 |
| EC4.1.3.42 | C0F | 0.02424836 |
| EC4.2.1.108 | C0F | 0.00317881 |
| EC4.2.1.115 | C0F | 0.00282561 |
| EC4.2.1.12 | C0F | 0.02424836 |
| EC4.2.1.19 | C0F | 0.04685679 |
| EC4.2.1.2 | C0F | 0.02694262 |
| EC4.2.1.47 | C0F | 0.02424836 |
| EC4.2.2.2 | C0F | 0.00526602 |
| EC4.2.2.8 | C0F | 0.0195883 |
| EC4.2.2.9 | C0F | 0.00282561 |
| EC4.2.99.21 | C0F | 0.01553232 |
| EC4.3.1.2 | C0F | 0.0194154 |
| EC4.4.1.25 | C0F | 0.00317881 |
| EC5.1.2.2 | C0F | 0.00317881 |
| EC5.1.3.7 | C0F | 0.02176478 |
| EC5.3.1.24 | C0F | 0.02694262 |
| EC5.3.1.28 | C0F | 0.02694262 |
| EC5.3.1.8 | C0F | 0.04000143 |
| EC5.3.2.4 | C0F | 0.00640228 |
| EC5.4.2.11 | C0F | 0.02424836 |
| EC5.4.99.1 | C0F | 0.01970856 |
| EC5.4.99.20 | C0F | 0.02694262 |
| EC5.4.99.5 | C0F | 0.04685679 |
| EC6.1.1.17 | C0F | 0.0486787 |
| EC6.1.1.22 | C0F | 0.03600129 |
| EC6.3.1.12 | C0F | 0.03041198 |
| EC6.3.2.- | C0F | 0.02694262 |
| EC6.3.2.29 | C0F | 0.01970856 |
| EC6.3.2.30 | C0F | 0.01970856 |
| EC6.3.3.3 | C0F | 0.02694262 |
| EC6.3.5.6 | C0F | 0.04685679 |
| EC6.3.5.7 | C0F | 0.04685679 |
| EC1.1.1.173 | C0M | 0.0111036 |
| EC1.1.1.2 | C0M | 0.01162834 |
| EC1.1.1.205 | C0M | 0.01375591 |
| EC1.1.1.220 | C0M | 0.04498312 |
| EC1.1.1.271 | C0M | 0.01793787 |
| EC1.1.1.29 | C0M | 0.02218168 |
| EC1.1.1.316 | C0M | 0.02425559 |
| EC1.1.1.367 | C0M | 0.00422007 |
| EC1.1.1.377 | C0M | 0.0111036 |
| EC1.1.1.378 | C0M | 0.0111036 |
| EC1.1.1.42 | C0M | 0.01375591 |
| EC1.1.1.88 | C0M | 0.0165976 |
| EC1.10.2.2 | C0M | 0.04498312 |
| EC1.12.5.1 | C0M | 0.0023623 |
| EC1.13.11.49 | C0M | 0.04367027 |
| EC1.14.13.8 | C0M | 0.00986987 |
| EC1.14.13.9 | C0M | 0.01018407 |
| EC1.14.19.3 | C0M | 0.0023623 |
| EC1.15.1.2 | C0M | 0.00986987 |
| EC1.17.1.8 | C0M | 0.03643988 |
| EC1.2.1.9 | C0M | 0.00864225 |
| EC1.2.7.- | C0M | 0.01375591 |
| EC1.2.7.1 | C0M | 0.01375591 |
| EC1.21.4.1 | C0M | 0.0023623 |
| EC1.21.98.1 | C0M | 0.0111036 |
| EC1.21.98.3 | C0M | 0.0126628 |
| EC1.3.1.101 | C0M | 0.00422007 |
| EC1.3.1.14 | C0M | 0.01739386 |
| EC1.3.1.31 | C0M | 0.01162834 |
| EC1.3.1.44 | C0M | 0.04367027 |
| EC1.3.1.74 | C0M | 0.00422007 |
| EC1.3.4.1 | C0M | 0.00864225 |
| EC1.3.7.11 | C0M | 0.00422007 |
| EC1.3.98.1 | C0M | 0.01331156 |
| EC1.5.1.5 | C0M | 0.01793787 |
| EC1.5.99.6 | C0M | 0.00986987 |
| EC1.6.3.4 | C0M | 0.01331156 |
| EC1.7.99.1 | C0M | 0.01375591 |
| EC1.8.1.9 | C0M | 0.01375591 |
| EC1.8.5.5 | C0M | 0.00422007 |
| EC2.1.1.148 | C0M | 0.00837129 |
| EC2.1.1.156 | C0M | 0.0165976 |
| EC2.1.1.191 | C0M | 0.01375591 |
| EC2.1.1.193 | C0M | 0.04048875 |
| EC2.1.1.217 | C0M | 0.01331156 |
| EC2.1.1.226 | C0M | 0.01331156 |
| EC2.1.1.227 | C0M | 0.01331156 |
| EC2.1.1.228 | C0M | 0.01375591 |
| EC2.1.1.45 | C0M | 0.01375591 |
| EC2.1.1.74 | C0M | 0.0165976 |
| EC2.1.3.2 | C0M | 0.01375591 |
| EC2.3.1.201 | C0M | 0.0096025 |
| EC2.3.1.203 | C0M | 0.00422007 |
| EC2.3.1.227 | C0M | 0.0111036 |
| EC2.3.1.234 | C0M | 0.01993097 |
| EC2.3.1.247 | C0M | 0.01162834 |
| EC2.3.1.35 | C0M | 0.01375591 |
| EC2.3.1.81 | C0M | 0.0023623 |
| EC2.3.2.10 | C0M | 0.0126628 |
| EC2.3.2.3 | C0M | 0.01739386 |
| EC2.3.3.10 | C0M | 0.01646718 |
| EC2.4.-.- | C0M | 0.01793787 |
| EC2.4.1.166 | C0M | 0.04796326 |
| EC2.4.1.175 | C0M | 0.00631345 |
| EC2.4.1.20 | C0M | 0.02464631 |
| EC2.4.1.208 | C0M | 0.0126628 |
| EC2.4.1.226 | C0M | 0.00631345 |
| EC2.4.1.250 | C0M | 0.01364393 |
| EC2.4.1.252 | C0M | 0.04885626 |
| EC2.4.1.292 | C0M | 0.0165976 |
| EC2.4.1.293 | C0M | 0.0023623 |
| EC2.4.1.315 | C0M | 0.01162834 |
| EC2.4.1.341 | C0M | 0.00986987 |
| EC2.4.1.44 | C0M | 0.0192519 |
| EC2.4.1.56 | C0M | 0.00631345 |
| EC2.4.1.57 | C0M | 0.01162834 |
| EC2.4.1.58 | C0M | 0.01162834 |
| EC2.4.1.7 | C0M | 0.04059894 |
| EC2.4.2.2 | C0M | 0.01331156 |
| EC2.4.2.29 | C0M | 0.04048875 |
| EC2.4.99.19 | C0M | 0.0023623 |
| EC2.5.1.120 | C0M | 0.04498312 |
| EC2.5.1.29 | C0M | 0.0126628 |
| EC2.5.1.30 | C0M | 0.04796326 |
| EC2.5.1.31 | C0M | 0.01993097 |
| EC2.5.1.54 | C0M | 0.02694262 |
| EC2.5.1.63 | C0M | 0.01375591 |
| EC2.5.1.97 | C0M | 0.0023623 |
| EC2.6.1.102 | C0M | 0.01298002 |
| EC2.6.1.106 | C0M | 0.01477748 |
| EC2.6.1.33 | C0M | 0.01162834 |
| EC2.6.1.34 | C0M | 0.0023623 |
| EC2.6.1.89 | C0M | 0.01477748 |
| EC2.6.1.92 | C0M | 0.00422007 |
| EC2.6.1.98 | C0M | 0.00837129 |
| EC2.7.1.144 | C0M | 0.0126628 |
| EC2.7.1.168 | C0M | 0.0111036 |
| EC2.7.1.204 | C0M | 0.0111036 |
| EC2.7.1.207 | C0M | 0.01979481 |
| EC2.7.1.23 | C0M | 0.02424836 |
| EC2.7.1.36 | C0M | 0.02390456 |
| EC2.7.1.40 | C0M | 0.01993097 |
| EC2.7.1.55 | C0M | 0.04021831 |
| EC2.7.1.76 | C0M | 0.02390456 |
| EC2.7.1.87 | C0M | 0.00631345 |
| EC2.7.1.90 | C0M | 0.02464631 |
| EC2.7.10.1 | C0M | 0.02218168 |
| EC2.7.11.- | C0M | 0.01331156 |
| EC2.7.2.1 | C0M | 0.01375591 |
| EC2.7.3.9 | C0M | 0.02856304 |
| EC2.7.4.- | C0M | 0.01331156 |
| EC2.7.4.2 | C0M | 0.0165976 |
| EC2.7.4.22 | C0M | 0.02570673 |
| EC2.7.4.3 | C0M | 0.01993097 |
| EC2.7.6.1 | C0M | 0.01375591 |
| EC2.7.6.2 | C0M | 0.01331156 |
| EC2.7.7.- | C0M | 0.02570673 |
| EC2.7.7.13 | C0M | 0.02856304 |
| EC2.7.7.27 | C0M | 0.01993097 |
| EC2.7.7.49 | C0M | 0.01375591 |
| EC2.7.7.77 | C0M | 0.04048875 |
| EC2.7.7.81 | C0M | 0.04498312 |
| EC2.7.7.82 | C0M | 0.00592428 |
| EC2.7.7.85 | C0M | 0.01375591 |
| EC2.7.7.9 | C0M | 0.01375591 |
| EC2.7.9.1 | C0M | 0.01331156 |
| EC2.7.9.3 | C0M | 0.02570673 |
| EC2.8.1.13 | C0M | 0.01375591 |
| EC2.8.1.7 | C0M | 0.01375591 |
| EC2.8.3.9 | C0M | 0.01739386 |
| EC2.8.4.4 | C0M | 0.01375591 |
| EC2.8.4.5 | C0M | 0.01162834 |
| EC3.1.-.- | C0M | 0.01375591 |
| EC3.1.1.29 | C0M | 0.02570673 |
| EC3.1.1.41 | C0M | 0.04494083 |
| EC3.1.1.57 | C0M | 0.0023623 |
| EC3.1.1.88 | C0M | 0.04912905 |
| EC3.1.1.92 | C0M | 0.0023623 |
| EC3.1.13.3 | C0M | 0.01375591 |
| EC3.1.2.21 | C0M | 0.01331156 |
| EC3.1.2.29 | C0M | 0.01503421 |
| EC3.1.21.2 | C0M | 0.02856304 |
| EC3.1.21.3 | C0M | 0.01375591 |
| EC3.1.26.3 | C0M | 0.01993097 |
| EC3.1.26.8 | C0M | 0.01331156 |
| EC3.1.3.41 | C0M | 0.01331156 |
| EC3.1.6.14 | C0M | 0.00837129 |
| EC3.1.6.6 | C0M | 0.01162834 |
| EC3.1.6.8 | C0M | 0.00837129 |
| EC3.2.1.133 | C0M | 0.01739386 |
| EC3.2.1.135 | C0M | 0.01739386 |
| EC3.2.1.17 | C0M | 0.02570673 |
| EC3.2.1.184 | C0M | 0.02500547 |
| EC3.2.1.54 | C0M | 0.01739386 |
| EC3.2.1.70 | C0M | 0.01364393 |
| EC3.2.1.85 | C0M | 0.01532267 |
| EC3.2.1.96 | C0M | 0.01364393 |
| EC3.2.2.1 | C0M | 0.01364393 |
| EC3.2.2.30 | C0M | 0.0111036 |
| EC3.4.11.21 | C0M | 0.0126628 |
| EC3.4.11.7 | C0M | 0.01375591 |
| EC3.4.14.11 | C0M | 0.0165976 |
| EC3.4.17.11 | C0M | 0.04367027 |
| EC3.4.21.107 | C0M | 0.04567381 |
| EC3.4.21.53 | C0M | 0.04048875 |
| EC3.4.21.72 | C0M | 0.01364393 |
| EC3.4.23.- | C0M | 0.01375591 |
| EC3.4.24.- | C0M | 0.02424836 |
| EC3.4.24.30 | C0M | 0.01233733 |
| EC3.4.24.78 | C0M | 0.01018407 |
| EC3.5.1.24 | C0M | 0.01364393 |
| EC3.5.1.28 | C0M | 0.02856304 |
| EC3.5.1.47 | C0M | 0.0165976 |
| EC3.5.1.9 | C0M | 0.02775145 |
| EC3.5.2.14 | C0M | 0.01844177 |
| EC3.5.4.40 | C0M | 0.0111036 |
| EC3.5.4.5 | C0M | 0.01375591 |
| EC3.5.4.9 | C0M | 0.01793787 |
| EC3.5.99.7 | C0M | 0.0429464 |
| EC3.6.1.57 | C0M | 0.0023623 |
| EC3.6.3.15 | C0M | 0.01331156 |
| EC3.6.3.31 | C0M | 0.04048875 |
| EC3.6.3.38 | C0M | 0.01162834 |
| EC3.6.3.4 | C0M | 0.03643988 |
| EC3.6.3.55 | C0M | 0.039985 |
| EC3.6.4.12 | C0M | 0.04567381 |
| EC3.6.4.6 | C0M | 0.04498312 |
| EC3.6.5.- | C0M | 0.02570673 |
| EC3.6.5.4 | C0M | 0.01375591 |
| EC4.1.1.23 | C0M | 0.01793787 |
| EC4.1.1.33 | C0M | 0.02390456 |
| EC4.1.1.70 | C0M | 0.0362616 |
| EC4.1.1.96 | C0M | 0.0023623 |
| EC4.1.3.4 | C0M | 0.00212607 |
| EC4.2.1.108 | C0M | 0.00986987 |
| EC4.2.1.11 | C0M | 0.03643988 |
| EC4.2.1.115 | C0M | 0.0023623 |
| EC4.2.1.151 | C0M | 0.0429464 |
| EC4.2.1.24 | C0M | 0.02856304 |
| EC4.2.1.47 | C0M | 0.04059894 |
| EC4.2.1.53 | C0M | 0.01780088 |
| EC4.2.2.2 | C0M | 0.00422007 |
| EC4.2.2.9 | C0M | 0.00863613 |
| EC4.2.99.21 | C0M | 0.0221519 |
| EC4.3.1.17 | C0M | 0.01375591 |
| EC4.3.2.2 | C0M | 0.02570673 |
| EC4.4.1.25 | C0M | 0.00986987 |
| EC5.1.2.2 | C0M | 0.04367027 |
| EC5.1.3.1 | C0M | 0.03643988 |
| EC5.3.1.26 | C0M | 0.02497631 |
| EC5.3.2.4 | C0M | 0.02222708 |
| EC5.4.99.1 | C0M | 0.0126628 |
| EC5.4.99.23 | C0M | 0.01375591 |
| EC5.4.99.5 | C0M | 0.04048875 |
| EC6.1.1.1 | C0M | 0.01375591 |
| EC6.1.1.10 | C0M | 0.02856304 |
| EC6.1.1.17 | C0M | 0.01993097 |
| EC6.1.1.20 | C0M | 0.04048875 |
| EC6.1.1.22 | C0M | 0.01375591 |
| EC6.1.1.24 | C0M | 0.01331156 |
| EC6.1.1.3 | C0M | 0.01375591 |
| EC6.1.1.4 | C0M | 0.01375591 |
| EC6.3.2.29 | C0M | 0.0126628 |
| EC6.3.2.30 | C0M | 0.0126628 |
| EC6.3.2.6 | C0M | 0.01793787 |
| EC6.3.2.7 | C0M | 0.0126628 |
| EC6.3.3.1 | C0M | 0.04567381 |
| EC6.3.4.3 | C0M | 0.01331156 |
| EC6.3.5.1 | C0M | 0.03941326 |
| EC6.3.5.6 | C0M | 0.01375591 |
| EC6.3.5.7 | C0M | 0.01375591 |
| EC1.1.1.159 | C1C2 | 0.04567381 |
| EC1.1.1.173 | C1C2 | 0.039985 |
| EC1.1.1.23 | C1C2 | 0.04059894 |
| EC1.1.1.271 | C1C2 | 0.03552408 |
| EC1.1.1.363 | C1C2 | 0.04567381 |
| EC1.1.1.377 | C1C2 | 0.039985 |
| EC1.1.1.378 | C1C2 | 0.039985 |
| EC1.1.1.42 | C1C2 | 0.03552408 |
| EC1.1.1.49 | C1C2 | 0.04567381 |
| EC1.1.1.85 | C1C2 | 0.04567381 |
| EC1.1.3.46 | C1C2 | 0.01667058 |
| EC1.11.1.5 | C1C2 | 0.04059894 |
| EC1.12.5.1 | C1C2 | 0.03498687 |
| EC1.12.98.1 | C1C2 | 0.04336913 |
| EC1.17.1.8 | C1C2 | 0.04059894 |
| EC1.2.1.58 | C1C2 | 0.01667058 |
| EC1.2.1.76 | C1C2 | 0.04974367 |
| EC1.2.1.88 | C1C2 | 0.04567381 |
| EC1.2.1.9 | C1C2 | 0.04059894 |
| EC1.2.7.- | C1C2 | 0.03552408 |
| EC1.2.7.1 | C1C2 | 0.03552408 |
| EC1.2.7.11 | C1C2 | 0.04567381 |
| EC1.2.7.3 | C1C2 | 0.04567381 |
| EC1.2.7.5 | C1C2 | 0.04567381 |
| EC1.21.98.1 | C1C2 | 0.03340275 |
| EC1.3.1.1 | C1C2 | 0.04567381 |
| EC1.3.1.10 | C1C2 | 0.04567381 |
| EC1.3.5.4 | C1C2 | 0.04567381 |
| EC1.3.99.24 | C1C2 | 0.03298093 |
| EC1.5.1.5 | C1C2 | 0.03552408 |
| EC1.5.3.22 | C1C2 | 0.01667058 |
| EC1.5.5.2 | C1C2 | 0.04567381 |
| EC1.7.2.2 | C1C2 | 0.04567381 |
| EC1.8.5.5 | C1C2 | 0.03044921 |
| EC1.8.99.2 | C1C2 | 0.01832274 |
| EC1.9.3.1 | C1C2 | 0.04567381 |
| EC2.1.1.113 | C1C2 | 0.04567381 |
| EC2.1.1.148 | C1C2 | 0.04059894 |
| EC2.1.1.185 | C1C2 | 0.04567381 |
| EC2.1.1.228 | C1C2 | 0.04567381 |
| EC2.1.2.9 | C1C2 | 0.04567381 |
| EC2.3.1.157 | C1C2 | 0.04567381 |
| EC2.3.1.180 | C1C2 | 0.04059894 |
| EC2.4.1.230 | C1C2 | 0.00833529 |
| EC2.4.1.7 | C1C2 | 0.04059894 |
| EC2.4.2.- | C1C2 | 0.04059894 |
| EC2.4.2.17 | C1C2 | 0.04567381 |
| EC2.4.2.28 | C1C2 | 0.04119509 |
| EC2.4.2.29 | C1C2 | 0.04567381 |
| EC2.5.1.3 | C1C2 | 0.04567381 |
| EC2.5.1.31 | C1C2 | 0.04567381 |
| EC2.5.1.55 | C1C2 | 0.04059894 |
| EC2.6.1.106 | C1C2 | 0.03340275 |
| EC2.6.1.52 | C1C2 | 0.04567381 |
| EC2.6.1.89 | C1C2 | 0.03035839 |
| EC2.7.1.168 | C1C2 | 0.03817457 |
| EC2.7.1.23 | C1C2 | 0.03552408 |
| EC2.7.1.83 | C1C2 | 0.04567381 |
| EC2.7.2.11 | C1C2 | 0.04059894 |
| EC2.7.2.8 | C1C2 | 0.04567381 |
| EC2.7.4.22 | C1C2 | 0.04059894 |
| EC2.7.6.1 | C1C2 | 0.03552408 |
| EC2.7.7.- | C1C2 | 0.04059894 |
| EC2.7.7.13 | C1C2 | 0.04567381 |
| EC2.7.7.23 | C1C2 | 0.04567381 |
| EC2.7.7.27 | C1C2 | 0.04059894 |
| EC2.7.7.33 | C1C2 | 0.02886516 |
| EC2.7.7.49 | C1C2 | 0.04059894 |
| EC2.7.7.71 | C1C2 | 0.0429464 |
| EC2.7.7.82 | C1C2 | 0.03817457 |
| EC2.7.7.9 | C1C2 | 0.04059894 |
| EC2.8.1.13 | C1C2 | 0.03552408 |
| EC2.8.1.6 | C1C2 | 0.04567381 |
| EC2.8.1.7 | C1C2 | 0.04567381 |
| EC2.8.3.9 | C1C2 | 0.03552408 |
| EC2.8.4.4 | C1C2 | 0.03552408 |
| EC2.8.4.5 | C1C2 | 0.04059894 |
| EC3.1.26.3 | C1C2 | 0.04567381 |
| EC3.1.3.15 | C1C2 | 0.04059894 |
| EC3.1.3.71 | C1C2 | 0.03035839 |
| EC3.1.4.57 | C1C2 | 0.03298093 |
| EC3.2.1.166 | C1C2 | 0.0429464 |
| EC3.2.1.17 | C1C2 | 0.04059894 |
| EC3.2.2.27 | C1C2 | 0.04567381 |
| EC3.2.2.30 | C1C2 | 0.03817457 |
| EC3.4.21.107 | C1C2 | 0.04567381 |
| EC3.4.23.- | C1C2 | 0.04059894 |
| EC3.4.23.43 | C1C2 | 0.04567381 |
| EC3.4.24.- | C1C2 | 0.03552408 |
| EC3.4.24.3 | C1C2 | 0.02222744 |
| EC3.4.24.84 | C1C2 | 0.0429464 |
| EC3.5.1.1 | C1C2 | 0.04059894 |
| EC3.5.1.44 | C1C2 | 0.02886516 |
| EC3.5.1.59 | C1C2 | 0.02886516 |
| EC3.5.1.88 | C1C2 | 0.04567381 |
| EC3.5.2.9 | C1C2 | 0.00833529 |
| EC3.5.3.26 | C1C2 | 0.04567381 |
| EC3.5.4.16 | C1C2 | 0.04059894 |
| EC3.5.4.25 | C1C2 | 0.04567381 |
| EC3.5.4.9 | C1C2 | 0.03552408 |
| EC3.5.99.7 | C1C2 | 0.0429464 |
| EC3.6.1.66 | C1C2 | 0.04567381 |
| EC3.6.4.12 | C1C2 | 0.04567381 |
| EC3.6.4.6 | C1C2 | 0.04498312 |
| EC3.6.5.- | C1C2 | 0.04059894 |
| EC3.6.5.4 | C1C2 | 0.04059894 |
| EC4.1.1.35 | C1C2 | 0.04292866 |
| EC4.1.1.48 | C1C2 | 0.04059894 |
| EC4.1.1.82 | C1C2 | 0.02886516 |
| EC4.1.1.96 | C1C2 | 0.03498687 |
| EC4.1.3.- | C1C2 | 0.04059894 |
| EC4.1.3.32 | C1C2 | 0.02886516 |
| EC4.1.3.4 | C1C2 | 0.01667058 |
| EC4.1.3.42 | C1C2 | 0.04059894 |
| EC4.1.99.1 | C1C2 | 0.04567381 |
| EC4.2.1.11 | C1C2 | 0.04059894 |
| EC4.2.1.115 | C1C2 | 0.03340275 |
| EC4.2.1.12 | C1C2 | 0.04059894 |
| EC4.2.1.151 | C1C2 | 0.0429464 |
| EC4.2.1.19 | C1C2 | 0.04567381 |
| EC4.2.1.33 | C1C2 | 0.04567381 |
| EC4.2.1.35 | C1C2 | 0.04567381 |
| EC4.2.1.45 | C1C2 | 0.01832274 |
| EC4.2.1.47 | C1C2 | 0.04059894 |
| EC4.2.1.70 | C1C2 | 0.04567381 |
| EC4.2.1.90 | C1C2 | 0.04567381 |
| EC4.2.3.1 | C1C2 | 0.04567381 |
| EC4.3.2.2 | C1C2 | 0.04059894 |
| EC4.6.1.12 | C1C2 | 0.04567381 |
| EC5.1.3.23 | C1C2 | 0.01832274 |
| EC5.3.1.- | C1C2 | 0.04567381 |
| EC5.3.2.4 | C1C2 | 0.03035839 |
| EC5.4.2.10 | C1C2 | 0.04567381 |
| EC5.4.2.8 | C1C2 | 0.04567381 |
| EC6.1.1.1 | C1C2 | 0.04059894 |
| EC6.1.1.10 | C1C2 | 0.04567381 |
| EC6.1.1.17 | C1C2 | 0.04059894 |
| EC6.1.1.20 | C1C2 | 0.04567381 |
| EC6.1.1.23 | C1C2 | 0.02931638 |
| EC6.1.1.3 | C1C2 | 0.04059894 |
| EC6.1.1.4 | C1C2 | 0.04059894 |
| EC6.3.2.13 | C1C2 | 0.04567381 |
| EC6.3.3.1 | C1C2 | 0.04567381 |
| EC6.3.4.20 | C1C2 | 0.04567381 |
| EC6.4.1.2 | C1C2 | 0.04567381 |
| EC1.1.1.137 | C1F | 0.04715163 |
| EC1.1.1.26 | C1F | 0.04395289 |
| EC1.1.1.271 | C1F | 0.03552408 |
| EC1.1.1.300 | C1F | 0.04395289 |
| EC1.1.1.337 | C1F | 0.03845878 |
| EC1.1.1.42 | C1F | 0.0486787 |
| EC1.11.2.4 | C1F | 0.04395289 |
| EC1.2.1.96 | C1F | 0.03890487 |
| EC1.2.7.- | C1F | 0.04056559 |
| EC1.2.7.1 | C1F | 0.04056559 |
| EC1.3.1.74 | C1F | 0.04041568 |
| EC1.3.3.5 | C1F | 0.04453 |
| EC1.3.99.33 | C1F | 0.04395289 |
| EC1.7.99.1 | C1F | 0.0486787 |
| EC1.8.5.5 | C1F | 0.03245247 |
| EC2.1.1.191 | C1F | 0.0486787 |
| EC2.1.1.219 | C1F | 0.03296467 |
| EC2.1.1.220 | C1F | 0.03296467 |
| EC2.1.1.234 | C1F | 0.03845878 |
| EC2.4.1.7 | C1F | 0.0486787 |
| EC2.4.2.28 | C1F | 0.03845878 |
| EC2.4.2.29 | C1F | 0.0486787 |
| EC2.6.1.106 | C1F | 0.04527741 |
| EC2.6.1.33 | C1F | 0.0486787 |
| EC2.6.1.89 | C1F | 0.03367974 |
| EC2.7.1.23 | C1F | 0.0486787 |
| EC2.7.1.8 | C1F | 0.03903222 |
| EC2.7.1.87 | C1F | 0.04527741 |
| EC2.7.7.27 | C1F | 0.0486787 |
| EC2.7.7.40 | C1F | 0.03296467 |
| EC2.7.7.49 | C1F | 0.04059894 |
| EC2.8.1.13 | C1F | 0.04056559 |
| EC2.8.4.4 | C1F | 0.04056559 |
| EC3.1.26.3 | C1F | 0.0486787 |
| EC3.1.3.96 | C1F | 0.03845878 |
| EC3.11.1.2 | C1F | 0.04715163 |
| EC3.2.1.11 | C1F | 0.02747055 |
| EC3.4.24.- | C1F | 0.04056559 |
| EC3.5.1.119 | C1F | 0.02747055 |
| EC3.6.3.6 | C1F | 0.03845878 |
| EC3.6.4.12 | C1F | 0.0486787 |
| EC3.6.5.4 | C1F | 0.0486787 |
| EC4.1.1.4 | C1F | 0.03845878 |
| EC4.1.3.4 | C1F | 0.01667058 |
| EC4.2.1.108 | C1F | 0.02723341 |
| EC4.2.1.12 | C1F | 0.0486787 |
| EC4.2.1.47 | C1F | 0.0486787 |
| EC5.1.1.21 | C1F | 0.03845878 |
| EC5.1.99.1 | C1F | 0.049447 |
| EC5.3.2.4 | C1F | 0.04041568 |
| EC6.1.1.17 | C1F | 0.0486787 |
| EC6.1.1.20 | C1F | 0.0486787 |
| EC6.1.1.22 | C1F | 0.0486787 |
| EC6.1.1.4 | C1F | 0.0486787 |
| EC6.3.1.19 | C1F | 0.03296467 |
| EC6.3.2.29 | C1F | 0.0486787 |
| EC6.3.2.30 | C1F | 0.0486787 |
| EC1.1.1.137 | C1M | 0.02500587 |
| EC1.1.1.26 | C1M | 0.04336913 |
| EC1.1.1.271 | C1M | 0.03552408 |
| EC1.1.1.300 | C1M | 0.03664548 |
| EC1.1.1.337 | C1M | 0.03298093 |
| EC1.1.1.367 | C1M | 0.03552408 |
| EC1.1.1.51 | C1M | 0.02222744 |
| EC1.11.2.4 | C1M | 0.02500587 |
| EC1.13.12.3 | C1M | 0.04336913 |
| EC1.2.1.68 | C1M | 0.03664548 |
| EC1.2.7.- | C1M | 0.03552408 |
| EC1.2.7.1 | C1M | 0.03552408 |
| EC1.21.98.3 | C1M | 0.039985 |
| EC1.3.1.31 | C1M | 0.04567381 |
| EC1.3.1.74 | C1M | 0.03035839 |
| EC1.3.3.5 | C1M | 0.03298093 |
| EC1.3.99.26 | C1M | 0.04336913 |
| EC1.3.99.28 | C1M | 0.04336913 |
| EC1.3.99.29 | C1M | 0.04336913 |
| EC1.3.99.31 | C1M | 0.04336913 |
| EC1.5.1.5 | C1M | 0.04943411 |
| EC1.7.1.6 | C1M | 0.03298093 |
| EC1.7.99.1 | C1M | 0.04059894 |
| EC1.8.5.5 | C1M | 0.03044921 |
| EC2.1.1.185 | C1M | 0.04567381 |
| EC2.1.1.191 | C1M | 0.04059894 |
| EC2.1.1.193 | C1M | 0.04567381 |
| EC2.1.1.219 | C1M | 0.02222744 |
| EC2.1.1.220 | C1M | 0.02222744 |
| EC2.1.1.228 | C1M | 0.04567381 |
| EC2.1.1.234 | C1M | 0.02500587 |
| EC2.1.1.45 | C1M | 0.04567381 |
| EC2.1.3.2 | C1M | 0.04059894 |
| EC2.3.1.234 | C1M | 0.04567381 |
| EC2.3.1.247 | C1M | 0.04498312 |
| EC2.4.1.208 | C1M | 0.04059894 |
| EC2.4.1.315 | C1M | 0.04567381 |
| EC2.4.1.58 | C1M | 0.04943411 |
| EC2.4.1.7 | C1M | 0.0486787 |
| EC2.4.2.29 | C1M | 0.04567381 |
| EC2.5.1.31 | C1M | 0.04567381 |
| EC2.5.1.54 | C1M | 0.04059894 |
| EC2.6.1.21 | C1M | 0.02222744 |
| EC2.6.1.33 | C1M | 0.03552408 |
| EC2.6.1.89 | C1M | 0.03035839 |
| EC2.7.1.40 | C1M | 0.04567381 |
| EC2.7.1.8 | C1M | 0.03903222 |
| EC2.7.1.87 | C1M | 0.03340275 |
| EC2.7.2.1 | C1M | 0.03552408 |
| EC2.7.3.9 | C1M | 0.04567381 |
| EC2.7.4.22 | C1M | 0.04059894 |
| EC2.7.4.3 | C1M | 0.04567381 |
| EC2.7.6.1 | C1M | 0.03552408 |
| EC2.7.7.- | C1M | 0.04059894 |
| EC2.7.7.13 | C1M | 0.04567381 |
| EC2.7.7.27 | C1M | 0.04059894 |
| EC2.7.7.40 | C1M | 0.02500587 |
| EC2.7.7.49 | C1M | 0.04059894 |
| EC2.8.1.13 | C1M | 0.03552408 |
| EC2.8.1.7 | C1M | 0.04567381 |
| EC2.8.3.9 | C1M | 0.04943411 |
| EC2.8.4.4 | C1M | 0.03552408 |
| EC3.1.26.3 | C1M | 0.04567381 |
| EC3.1.3.96 | C1M | 0.03298093 |
| EC3.11.1.2 | C1M | 0.03903222 |
| EC3.2.1.11 | C1M | 0.01944901 |
| EC3.4.19.1 | C1M | 0.03298093 |
| EC3.4.24.- | C1M | 0.03552408 |
| EC3.4.24.78 | C1M | 0.04567381 |
| EC3.5.1.119 | C1M | 0.02222744 |
| EC3.5.1.28 | C1M | 0.04567381 |
| EC3.5.4.9 | C1M | 0.04943411 |
| EC3.6.3.31 | C1M | 0.04567381 |
| EC3.6.3.6 | C1M | 0.03298093 |
| EC3.6.4.12 | C1M | 0.04567381 |
| EC3.6.5.- | C1M | 0.04059894 |
| EC3.6.5.4 | C1M | 0.04059894 |
| EC4.1.1.4 | C1M | 0.03298093 |
| EC4.1.3.4 | C1M | 0.01667058 |
| EC4.2.1.108 | C1M | 0.0481086 |
| EC4.2.1.11 | C1M | 0.04059894 |
| EC4.2.1.47 | C1M | 0.0486787 |
| EC4.2.1.90 | C1M | 0.04567381 |
| EC4.3.1.17 | C1M | 0.04059894 |
| EC4.3.2.2 | C1M | 0.04059894 |
| EC5.1.1.21 | C1M | 0.03298093 |
| EC5.4.2.10 | C1M | 0.04567381 |
| EC5.4.2.8 | C1M | 0.04567381 |
| EC5.4.99.5 | C1M | 0.04567381 |
| EC6.1.1.1 | C1M | 0.04059894 |
| EC6.1.1.10 | C1M | 0.04567381 |
| EC6.1.1.17 | C1M | 0.04059894 |
| EC6.1.1.20 | C1M | 0.04567381 |
| EC6.1.1.22 | C1M | 0.04059894 |
| EC6.1.1.3 | C1M | 0.04059894 |
| EC6.1.1.4 | C1M | 0.04059894 |
| EC6.3.1.19 | C1M | 0.02222744 |
| EC6.3.2.13 | C1M | 0.04567381 |
| EC6.3.2.29 | C1M | 0.03552408 |
| EC6.3.2.30 | C1M | 0.03552408 |
| EC6.3.2.7 | C1M | 0.04059894 |
| EC6.4.1.3 | C1M | 0.03817457 |
| EC1.1.1.137 | C2F | 0.04715163 |
| EC1.1.1.173 | C2F | 0.039985 |
| EC1.1.1.26 | C2F | 0.04395289 |
| EC1.1.1.261 | C2F | 0.04395289 |
| EC1.1.1.271 | C2F | 0.03552408 |
| EC1.1.1.287 | C2F | 0.02747055 |
| EC1.1.1.300 | C2F | 0.04395289 |
| EC1.1.1.325 | C2F | 0.02747055 |
| EC1.1.1.337 | C2F | 0.03845878 |
| EC1.1.1.370 | C2F | 0.04715163 |
| EC1.1.1.377 | C2F | 0.039985 |
| EC1.1.1.378 | C2F | 0.039985 |
| EC1.1.1.39 | C2F | 0.02747055 |
| EC1.1.1.9 | C2F | 0.02747055 |
| EC1.1.3.46 | C2F | 0.02747055 |
| EC1.12.5.1 | C2F | 0.03498687 |
| EC1.14.13.9 | C2F | 0.04056559 |
| EC1.14.19.3 | C2F | 0.04056559 |
| EC1.2.1.58 | C2F | 0.02747055 |
| EC1.2.1.76 | C2F | 0.02747055 |
| EC1.21.4.3 | C2F | 0.03845878 |
| EC1.21.4.4 | C2F | 0.03845878 |
| EC1.21.98.1 | C2F | 0.04056559 |
| EC1.3.3.5 | C2F | 0.04453 |
| EC1.3.99.33 | C2F | 0.04395289 |
| EC1.4.1.16 | C2F | 0.04367027 |
| EC1.5.3.22 | C2F | 0.02747055 |
| EC1.5.98.2 | C2F | 0.02747055 |
| EC1.7.1.6 | C2F | 0.04781648 |
| EC1.7.99.1 | C2F | 0.0486787 |
| EC1.8.99.2 | C2F | 0.02197644 |
| EC2.1.1.148 | C2F | 0.04676606 |
| EC2.1.1.191 | C2F | 0.0486787 |
| EC2.1.1.219 | C2F | 0.03296467 |
| EC2.1.1.220 | C2F | 0.03296467 |
| EC2.1.1.234 | C2F | 0.03845878 |
| EC2.1.1.328 | C2F | 0.02747055 |
| EC2.3.1.180 | C2F | 0.0486787 |
| EC2.3.1.203 | C2F | 0.0486787 |
| EC2.3.1.81 | C2F | 0.04056559 |
| EC2.3.2.17 | C2F | 0.04395289 |
| EC2.4.1.161 | C2F | 0.02747055 |
| EC2.4.1.230 | C2F | 0.00833529 |
| EC2.4.1.292 | C2F | 0.0486787 |
| EC2.4.1.293 | C2F | 0.04056559 |
| EC2.4.1.4 | C2F | 0.03845878 |
| EC2.4.2.28 | C2F | 0.03845878 |
| EC2.4.2.45 | C2F | 0.02747055 |
| EC2.4.99.19 | C2F | 0.04781648 |
| EC2.5.1.97 | C2F | 0.0486787 |
| EC2.6.1.102 | C2F | 0.0486787 |
| EC2.6.1.108 | C2F | 0.02747055 |
| EC2.6.1.21 | C2F | 0.04527741 |
| EC2.6.1.34 | C2F | 0.04781648 |
| EC2.6.1.83 | C2F | 0.03845878 |
| EC2.6.1.92 | C2F | 0.0486787 |
| EC2.6.1.98 | C2F | 0.0486787 |
| EC2.7.1.168 | C2F | 0.04527741 |
| EC2.7.1.204 | C2F | 0.0486787 |
| EC2.7.1.63 | C2F | 0.049447 |
| EC2.7.1.8 | C2F | 0.03903222 |
| EC2.7.14.1 | C2F | 0.049447 |
| EC2.7.7.33 | C2F | 0.02886516 |
| EC2.7.7.40 | C2F | 0.03296467 |
| EC2.7.7.74 | C2F | 0.049447 |
| EC2.7.7.82 | C2F | 0.04781648 |
| EC2.7.8.34 | C2F | 0.049447 |
| EC2.7.8.40 | C2F | 0.04395289 |
| EC2.7.8.41 | C2F | 0.03845878 |
| EC3.1.3.71 | C2F | 0.03296467 |
| EC3.1.3.96 | C2F | 0.03845878 |
| EC3.1.4.57 | C2F | 0.04920489 |
| EC3.11.1.2 | C2F | 0.04715163 |
| EC3.2.1.11 | C2F | 0.02747055 |
| EC3.2.1.132 | C2F | 0.0397828 |
| EC3.2.1.184 | C2F | 0.0486787 |
| EC3.2.1.185 | C2F | 0.02747055 |
| EC3.2.1.46 | C2F | 0.02729392 |
| EC3.2.1.58 | C2F | 0.03845878 |
| EC3.2.2.30 | C2F | 0.04781648 |
| EC3.4.11.19 | C2F | 0.03845878 |
| EC3.4.14.5 | C2F | 0.02729392 |
| EC3.4.24.3 | C2F | 0.04715163 |
| EC3.5.1.106 | C2F | 0.02747055 |
| EC3.5.1.119 | C2F | 0.02747055 |
| EC3.5.1.44 | C2F | 0.02886516 |
| EC3.5.1.59 | C2F | 0.02886516 |
| EC3.5.2.9 | C2F | 0.00833529 |
| EC3.5.3.18 | C2F | 0.04301988 |
| EC3.5.99.7 | C2F | 0.04781648 |
| EC3.6.1.57 | C2F | 0.0486787 |
| EC3.6.3.4 | C2F | 0.0486787 |
| EC3.6.3.55 | C2F | 0.0486787 |
| EC3.6.3.6 | C2F | 0.03845878 |
| EC3.6.4.12 | C2F | 0.0486787 |
| EC3.6.4.6 | C2F | 0.04498312 |
| EC4.1.1.35 | C2F | 0.03845878 |
| EC4.1.1.4 | C2F | 0.03845878 |
| EC4.1.1.82 | C2F | 0.02886516 |
| EC4.1.1.96 | C2F | 0.03498687 |
| EC4.1.2.29 | C2F | 0.02747055 |
| EC4.1.3.32 | C2F | 0.02886516 |
| EC4.2.1.115 | C2F | 0.04056559 |
| EC4.2.1.12 | C2F | 0.0486787 |
| EC4.2.1.151 | C2F | 0.04527741 |
| EC4.2.1.45 | C2F | 0.02197644 |
| EC4.2.1.77 | C2F | 0.02747055 |
| EC4.3.2.2 | C2F | 0.0486787 |
| EC5.1.1.17 | C2F | 0.02747055 |
| EC5.1.1.21 | C2F | 0.03845878 |
| EC5.1.1.4 | C2F | 0.02747055 |
| EC5.1.3.23 | C2F | 0.02197644 |
| EC5.1.99.1 | C2F | 0.049447 |
| EC6.1.1.23 | C2F | 0.03845878 |
| EC6.3.1.19 | C2F | 0.03296467 |
| EC6.4.1.3 | C2F | 0.0486787 |
| EC1.1.1.137 | C2M | 0.02500587 |
| EC1.1.1.173 | C2M | 0.039985 |
| EC1.1.1.2 | C2M | 0.04567381 |
| EC1.1.1.220 | C2M | 0.04498312 |
| EC1.1.1.26 | C2M | 0.04336913 |
| EC1.1.1.261 | C2M | 0.03298093 |
| EC1.1.1.271 | C2M | 0.03552408 |
| EC1.1.1.287 | C2M | 0.01667058 |
| EC1.1.1.300 | C2M | 0.03664548 |
| EC1.1.1.325 | C2M | 0.01667058 |
| EC1.1.1.337 | C2M | 0.03298093 |
| EC1.1.1.370 | C2M | 0.02222744 |
| EC1.1.1.377 | C2M | 0.039985 |
| EC1.1.1.378 | C2M | 0.039985 |
| EC1.1.1.39 | C2M | 0.01667058 |
| EC1.1.1.51 | C2M | 0.02222744 |
| EC1.1.1.9 | C2M | 0.01667058 |
| EC1.1.3.46 | C2M | 0.04607562 |
| EC1.10.2.2 | C2M | 0.04498312 |
| EC1.11.1.1 | C2M | 0.04567381 |
| EC1.12.5.1 | C2M | 0.03498687 |
| EC1.13.12.3 | C2M | 0.04336913 |
| EC1.14.13.9 | C2M | 0.03044921 |
| EC1.14.19.3 | C2M | 0.03552408 |
| EC1.2.1.58 | C2M | 0.02747055 |
| EC1.2.1.68 | C2M | 0.03664548 |
| EC1.2.1.76 | C2M | 0.01667058 |
| EC1.2.7.11 | C2M | 0.04567381 |
| EC1.2.7.3 | C2M | 0.04567381 |
| EC1.21.4.3 | C2M | 0.02222744 |
| EC1.21.4.4 | C2M | 0.02222744 |
| EC1.21.98.1 | C2M | 0.03340275 |
| EC1.3.1.101 | C2M | 0.03044921 |
| EC1.3.1.31 | C2M | 0.04567381 |
| EC1.3.3.5 | C2M | 0.03298093 |
| EC1.3.4.1 | C2M | 0.03044921 |
| EC1.3.5.4 | C2M | 0.04567381 |
| EC1.3.7.11 | C2M | 0.03044921 |
| EC1.3.99.26 | C2M | 0.04336913 |
| EC1.3.99.28 | C2M | 0.04336913 |
| EC1.3.99.29 | C2M | 0.04336913 |
| EC1.3.99.31 | C2M | 0.04336913 |
| EC1.3.99.32 | C2M | 0.03298093 |
| EC1.3.99.33 | C2M | 0.03903222 |
| EC1.4.1.1 | C2M | 0.04498312 |
| EC1.4.7.1 | C2M | 0.03298093 |
| EC1.5.1.7 | C2M | 0.04567381 |
| EC1.5.3.22 | C2M | 0.02747055 |
| EC1.5.98.2 | C2M | 0.01667058 |
| EC1.7.1.6 | C2M | 0.03298093 |
| EC1.7.99.1 | C2M | 0.04059894 |
| EC1.8.99.2 | C2M | 0.01667058 |
| EC1.9.3.1 | C2M | 0.04567381 |
| EC2.1.1.113 | C2M | 0.04567381 |
| EC2.1.1.191 | C2M | 0.04059894 |
| EC2.1.1.219 | C2M | 0.02222744 |
| EC2.1.1.220 | C2M | 0.02222744 |
| EC2.1.1.234 | C2M | 0.02500587 |
| EC2.1.1.328 | C2M | 0.01667058 |
| EC2.10.1.1 | C2M | 0.04567381 |
| EC2.3.1.180 | C2M | 0.04059894 |
| EC2.3.1.203 | C2M | 0.03552408 |
| EC2.3.1.247 | C2M | 0.04498312 |
| EC2.3.1.31 | C2M | 0.0277843 |
| EC2.3.1.81 | C2M | 0.03552408 |
| EC2.3.1.89 | C2M | 0.04567381 |
| EC2.3.2.16 | C2M | 0.03664548 |
| EC2.3.2.17 | C2M | 0.02500587 |
| EC2.3.2.8 | C2M | 0.0429464 |
| EC2.4.1.153 | C2M | 0.0407943 |
| EC2.4.1.161 | C2M | 0.01667058 |
| EC2.4.1.230 | C2M | 0.00833529 |
| EC2.4.1.250 | C2M | 0.03552408 |
| EC2.4.1.293 | C2M | 0.03552408 |
| EC2.4.1.4 | C2M | 0.02222744 |
| EC2.4.1.44 | C2M | 0.04059894 |
| EC2.4.1.5 | C2M | 0.04567381 |
| EC2.4.1.52 | C2M | 0.04567381 |
| EC2.4.2.28 | C2M | 0.0306729 |
| EC2.4.2.29 | C2M | 0.04567381 |
| EC2.4.2.45 | C2M | 0.01667058 |
| EC2.4.99.19 | C2M | 0.04059894 |
| EC2.5.1.101 | C2M | 0.04567381 |
| EC2.5.1.120 | C2M | 0.04498312 |
| EC2.5.1.3 | C2M | 0.04567381 |
| EC2.5.1.97 | C2M | 0.03552408 |
| EC2.6.1.102 | C2M | 0.04059894 |
| EC2.6.1.108 | C2M | 0.01667058 |
| EC2.6.1.21 | C2M | 0.02222744 |
| EC2.6.1.34 | C2M | 0.04059894 |
| EC2.6.1.83 | C2M | 0.02931638 |
| EC2.6.1.9 | C2M | 0.04567381 |
| EC2.6.1.92 | C2M | 0.03552408 |
| EC2.6.1.98 | C2M | 0.03552408 |
| EC2.7.1.163 | C2M | 0.0429464 |
| EC2.7.1.168 | C2M | 0.03817457 |
| EC2.7.1.206 | C2M | 0.04567381 |
| EC2.7.1.8 | C2M | 0.03903222 |
| EC2.7.14.1 | C2M | 0.03664548 |
| EC2.7.7.33 | C2M | 0.02886516 |
| EC2.7.7.39 | C2M | 0.0429464 |
| EC2.7.7.40 | C2M | 0.02500587 |
| EC2.7.7.53 | C2M | 0.04498312 |
| EC2.7.7.71 | C2M | 0.0429464 |
| EC2.7.7.81 | C2M | 0.04498312 |
| EC2.7.7.82 | C2M | 0.03817457 |
| EC2.7.8.36 | C2M | 0.0429464 |
| EC2.7.8.40 | C2M | 0.03298093 |
| EC2.7.8.41 | C2M | 0.02222744 |
| EC2.7.8.44 | C2M | 0.04567381 |
| EC3.1.3.71 | C2M | 0.02931638 |
| EC3.1.3.96 | C2M | 0.03298093 |
| EC3.1.4.37 | C2M | 0.04227928 |
| EC3.1.4.57 | C2M | 0.04920489 |
| EC3.11.1.2 | C2M | 0.03903222 |
| EC3.2.1.10 | C2M | 0.04567381 |
| EC3.2.1.11 | C2M | 0.01944901 |
| EC3.2.1.132 | C2M | 0.02500587 |
| EC3.2.1.184 | C2M | 0.04059894 |
| EC3.2.1.185 | C2M | 0.01667058 |
| EC3.2.1.45 | C2M | 0.04498312 |
| EC3.2.1.46 | C2M | 0.01667058 |
| EC3.2.1.58 | C2M | 0.02222744 |
| EC3.2.2.27 | C2M | 0.04567381 |
| EC3.2.2.30 | C2M | 0.03817457 |
| EC3.4.11.19 | C2M | 0.03469531 |
| EC3.4.11.5 | C2M | 0.04567381 |
| EC3.4.14.5 | C2M | 0.01667058 |
| EC3.4.19.1 | C2M | 0.03298093 |
| EC3.4.22.40 | C2M | 0.04498312 |
| EC3.4.23.43 | C2M | 0.04567381 |
| EC3.4.24.84 | C2M | 0.0429464 |
| EC3.5.1.106 | C2M | 0.01667058 |
| EC3.5.1.119 | C2M | 0.02222744 |
| EC3.5.1.44 | C2M | 0.02886516 |
| EC3.5.1.53 | C2M | 0.04567381 |
| EC3.5.1.59 | C2M | 0.02886516 |
| EC3.5.2.9 | C2M | 0.00833529 |
| EC3.5.3.18 | C2M | 0.03240824 |
| EC3.5.3.26 | C2M | 0.04567381 |
| EC3.5.4.25 | C2M | 0.04567381 |
| EC3.5.4.40 | C2M | 0.04059894 |
| EC3.5.99.7 | C2M | 0.0429464 |
| EC3.6.1.57 | C2M | 0.03552408 |
| EC3.6.3.31 | C2M | 0.04567381 |
| EC3.6.3.4 | C2M | 0.03815969 |
| EC3.6.3.55 | C2M | 0.039985 |
| EC3.6.3.6 | C2M | 0.03298093 |
| EC3.6.4.6 | C2M | 0.04498312 |
| EC4.1.1.35 | C2M | 0.02222744 |
| EC4.1.1.4 | C2M | 0.03298093 |
| EC4.1.1.82 | C2M | 0.02886516 |
| EC4.1.1.96 | C2M | 0.03498687 |
| EC4.1.2.29 | C2M | 0.01667058 |
| EC4.1.2.43 | C2M | 0.04059894 |
| EC4.1.3.- | C2M | 0.04059894 |
| EC4.1.3.32 | C2M | 0.02886516 |
| EC4.2.1.115 | C2M | 0.03340275 |
| EC4.2.1.12 | C2M | 0.04059894 |
| EC4.2.1.135 | C2M | 0.04567381 |
| EC4.2.1.151 | C2M | 0.0429464 |
| EC4.2.1.45 | C2M | 0.01667058 |
| EC4.2.1.77 | C2M | 0.01667058 |
| EC4.2.99.20 | C2M | 0.04567381 |
| EC4.4.1.21 | C2M | 0.04567381 |
| EC5.1.1.17 | C2M | 0.01667058 |
| EC5.1.1.21 | C2M | 0.03298093 |
| EC5.1.1.4 | C2M | 0.01667058 |
| EC5.1.3.23 | C2M | 0.01667058 |
| EC5.1.99.1 | C2M | 0.04771822 |
| EC5.3.1.27 | C2M | 0.04567381 |
| EC6.1.1.22 | C2M | 0.04059894 |
| EC6.1.1.23 | C2M | 0.03845878 |
| EC6.3.1.19 | C2M | 0.02222744 |
| EC6.4.1.1 | C2M | 0.04567381 |
| EC6.4.1.3 | C2M | 0.03817457 |
| EC1.3.1.101 | MF | 0.04056559 |
| EC1.3.7.11 | MF | 0.04056559 |
